# CircRNA MBOAT2 promotes intrahepatic cholangiocarcinoma progression and lipid metabolism reprogramming by stabilizing PTBP1 to facilitate FASN mRNA cytoplasmic export

**DOI:** 10.1038/s41419-022-05540-y

**Published:** 2023-01-12

**Authors:** Xiaopeng Yu, Huanjun Tong, Jialu Chen, Chenwei Tang, Shuqing Wang, Yu Si, Shouhua Wang, Zhaohui Tang

**Affiliations:** 1 Department of General Surgery, Xinhua Hospital, Shanghai Jiao Tong University School of Medicine, Shanghai, 200092 China; 2 Shanghai Key Laboratory of Biliary Tract Disease Research, Xinhua Hospital, Shanghai Jiao Tong University School of Medicine, Shanghai, 200092 China; 3 Department of Blood Transfusion, Xinhua Hospital, Shanghai Jiao Tong University School of Medicine, Shanghai, 200092 China

**Keywords:** Cancer metabolism, Bile duct cancer, Non-coding RNAs

## Abstract

The carcinogenic role of FASN by regulating lipid metabolism reprogramming has been well-established in multiple tumors. However, whether mechanisms during intrahepatic cholangiocarcinoma (ICC) progression, such as circRNAs, regulate FASN expression remains unknown. Here we demonstrate a lipid metabolism-related circRNA, circMBOAT2 (hsa_circ_0007334 in circBase), frequently upregulated in ICC tissues, and positively correlated with ICC malignant features. CircMBOAT2 knockdown inhibits the growth and metastasis of ICC cells. Mechanistically, circMBOAT2 combines with PTBP1 and protects PTBP1 from ubiquitin/proteasome-dependent degradation, impairing the function of PTBP1 to transfer FASN mRNA from the nucleus to the cytoplasm. Moreover, circMBOAT2 and FASN have the same effect on fatty acid profile, unsaturated fatty acids instead of saturated fatty acids are primarily regulated and associated with malignant behaviors of ICC cells. The levels of lipid peroxidation and ROS were significantly higher when FASN was knocked down and recovered when circMBOAT2 was overexpressed. Our results identified that circMBOAT2 was upregulated in ICC and promoted progression by stabilizing PTBP1 to facilitate FASN mRNA cytoplasmic export, which altered lipid metabolic profile and regulated redox homeostasis in ICC, suggesting that circMBOAT2 may serve as an available therapeutic target for ICC with active lipid metabolism.

## Background

Intrahepatic cholangiocarcinoma (ICC) accounts for 8–10% of biliary tract cancers (BTC) and 10–15% of primary liver cancer [1], including mass-forming (MF), periductal infiltrating (PI), and intraductal growth (IG) type [2]. The incidence of ICC has increased globally over the past two decades [3, 4]. Due to its highly aggressive and malignant biological behavior, as well as the lack of effective treatments, ICC has an extremely poor prognosis, especially for advanced-stage patients [4]. This poor long-term survival outcome highlights further improvements in disease control by understanding the mechanisms of ICC. Although many previous reports have documented that abundant molecular anomalies, in either coding or non-coding RNAs [5, 6], are involved and play important roles in the pathogenic process of ICC, the precise molecular mechanisms remain largely unclear.

Circular RNAs (circRNAs) compromise a class of regulatory RNAs with covalently closed single-stranded loop conformations produced by direct backsplicing or exon skipping of precursor mRNA [7]. Dysregulation of circRNA expression has been found in different pathological processes, including the pathogenesis of breast [8], liver [9], lung [10], and esophagus [11] cancers. In the past, circRNAs were considered by-products of splicing errors with little function [12]. Several circRNA functions, such as miRNA sponges [8, 13], protein scaffold [10, 14], and protein translation templates [5, 15], have been identified over the years. These findings suggest that circRNAs play a functional role in biological processes and act as potential clinical molecular markers. As a consequence, this study provides new insights into the treatment of cancer and other human diseases.

Tumor cells live in an environment of hypoxia and a relative lack of nutrients. In order to meet the energy needs of cells and their biosynthetic materials during rapid tumor growth, their metabolic pattern changes, which is called metabolic reprogramming [16]. Metabolic reprogramming is one of the hallmarks of malignant tumors [17], with no exception for ICC. ICC is a characteristic lactic dehydrogenase (IDH) mutation, which was found in nearly 20% of ICC patients by whole-exome sequencing [18]. Hexokinase (HK), a key enzyme that catalyzes the conversion of glucose to glucose-6-phosphate, is abnormally elevated in ICC associated with liver flukes and is associated with poor prognosis [19]. However, the mechanisms of metabolic reprogramming in these studies have mainly focused on glycometabolism. Further insight into the role and mechanism of lipid metabolism reprogramming in ICC may be helpful in understanding the development and progression of ICC. Fatty acid synthase (FASN) is a key enzyme involved in the fatty acid synthesis. It has been reported that FASN plays a dominant role in tumors such as breast [20], ovary [21], liver [22], and colorectum [23] cancers. High expression of FASN is significantly correlated with advanced stages in cholangiocarcinoma patients [24]. Knockdown of FASN inhibits ICC cell proliferation and invasion [24]; however, the mechanism regulating FASN expression and function in ICC and the changes in the metabolic profile regulated by FASN are still unclear.

In the present study, we demonstrated that a specific circRNA associated with lipid metabolic reprogramming, circMBOAT2 (hsa_circ_0007334 in circBase), mapped to the chromosome 2p25 amplicon in ICC, is frequently upregulated in patients with ICC and predicts poor survival. We further revealed that circMBOAT2 could bind to polypyrimidine tract binding protein 1 (PTBP1), a ribosomal protein that has never been reported in ICC. This protects PTBP1 from degradation in a ubiquitin-dependent manner. PTBP1, binds and exports FASN mRNA to the cytoplasm sequentially, resulting in high translation of FASN. Lipidomics indicated that circMBOAT2 and FASN have the same effect on fatty acid levels; unsaturated fatty acids instead of saturated fatty acids are primarily regulated by them. The levels of lipid peroxidation and ROS were significantly higher when FASN was knocked down and recovered when circMBOAT2 was overexpressed. Our data suggest that circMBOAT2 may exert as a potential therapeutic target against ICC.

## Results

### CircMBOAT2 is an upregulated circRNA associated with lipid metabolism in ICC

**Fig. 1:**
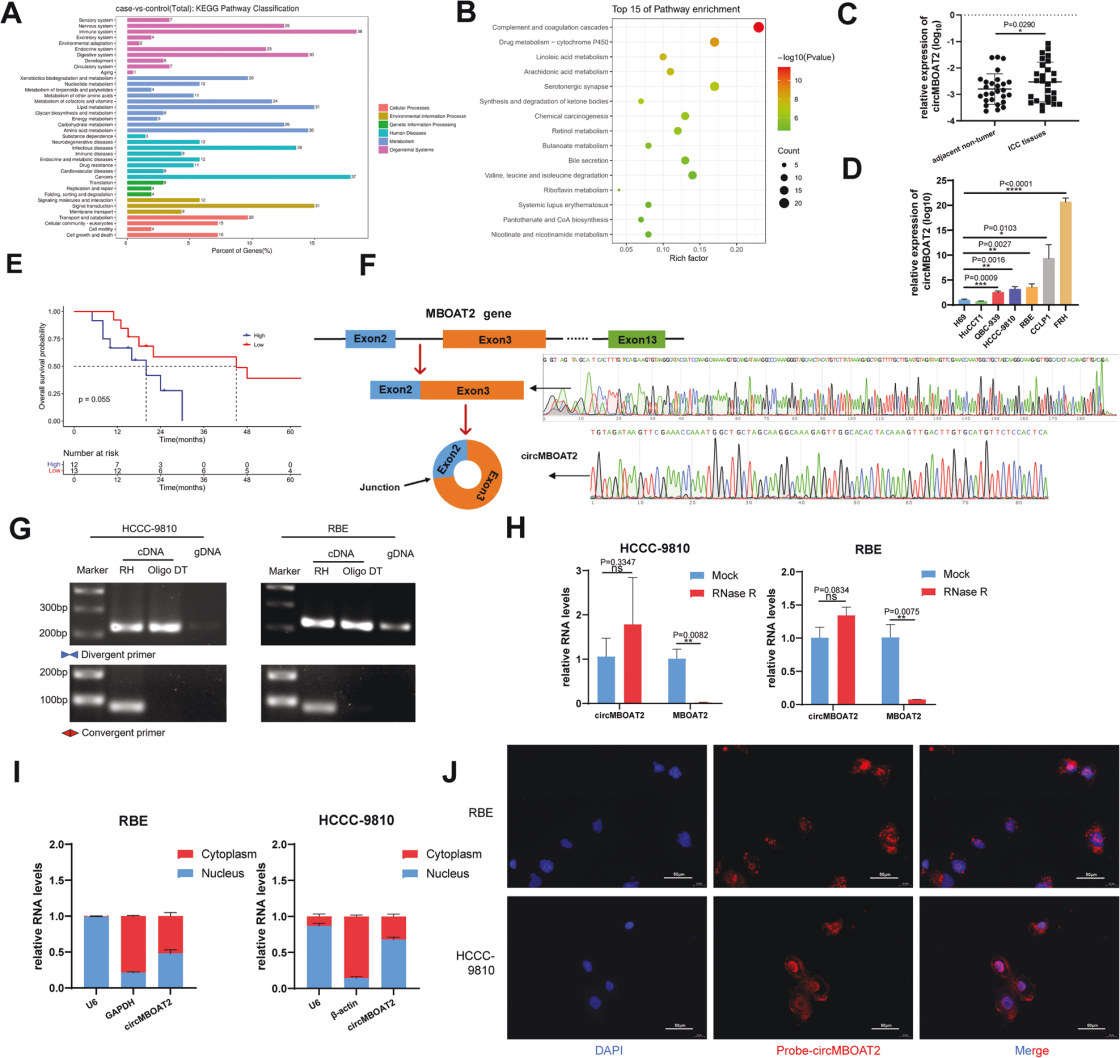
CircMBOAT2 is an upregulated circRNA associated with lipid metabolism in ICC. **A**, **B** KEGG pathway classification analysis and KEGG enrichment analysis for top 15 pathways in ICC tissue compared to adjacent non-tumor tissue by RNA sequencing. **C** The qRT-PCR method was applied to detect the expression levels of circMBOAT2 in 27 ICC patients and normal tissues adjacent to cancer. The expression of circMBOAT2 was normalized to β-actin. Significant differences between groups were analyzed by paired sample *t*-test. **D** The relative expression of circMBOAT2 in six human ICC cell lines and one human normal bile duct epithelial cell line (H69) by qRT-PCR. **E** *K*–*M* curve showed the prognosis of ICC patients with different expressions of circMBOAT2. **F** Explanation of the illustrated genomic loci of MBOAT2 and validation strategy for circular exons 2–3 (circMBOAT2). Sanger sequencing after PCR revealed MBOAT2 exons 2–3 and “head and tail” splicing of circMBOAT2. **G** PCR analysis of circMBOAT2 by divergent primers and convergent primers in cDNA and genomic DNA. RH random hexamers, OdT oligo(dT)18 primers, gDNA genomic DNA. **H** Relative RNA levels of RNase R-treated circMBOAT2 and linear MBOAT2 by qRT-PCR. **I** The nuclear and cytoplasmic fractions was obtained by isolation. circMBOAT2 is localized in both the nucleus and cytoplasm. U6 is abundantly expressed in the nucleus and GAPDH or β-actin is mainly present in the cytoplasm and served as a nuclear and cytoplasmic RNA marker. **J** Fluorescence in situ hybridization (FISH) of RNA was performed on circMBOAT2. Nuclei were stained with 4,6-diamino-2-phenylindole (DAPI). Scale bar = 50 μm. Significant differences between groups were analyzed by *t*-test. Error bars represent the means ± SEM of three independent experiments.

Metabolism reprogramming is a hallmark of cancer, with no exception of ICC [25]. We analyzed the expression profiles of circRNAs in seven paired ICC samples using RNA-seq. A total of 420 dysregulated circRNAs meeting the following requirements were identified in ICC tissues: (1) |average normalized fold change| ≥ 1; (2) *P* value < 0.05, of which 82 were upregulated and 338 were downregulated (Fig. S1A–C). Next, gene functions of the selected circRNAs were interpreted by Gene Ontology (GO) terms and Kyoto Encyclopedia of Genes and Genomes (KEGG) pathway analysis. In the three main categories of GO analysis, the assignments to “biological process” occupied most of the genes, followed by “cellular component” and “molecular function”, “single-organism process”, “cellular process”, and “metabolic process” were superior to the “biological process” category (Fig. S1D). In the KEGG pathway classification analysis, “Organismal Systems” (154 genes) and “metabolism” (169 genes) categories were predominant. Among the “metabolism” category, “lipid metabolism” included 31 genes (Fig. 1A). Moreover, KEGG enrichment analysis indicated that the selected circRNAs were mainly related to metabolism, including “Linoleic acid metabolism” and “Arachidonic acid metabolism” (Fig. 1B). These results suggested that the significantly differential circRNAs in ICC were related to lipid metabolism. Among the circRNAs related to lipid metabolism, circMBOAT2 has the most obvious difference between ICC tumors and adjacent normal tissues. We then confirmed differential expression between ICC and normal biliary tissues/cells **(**Fig. 1C, D). We next analyzed the correlation between circMBOAT2 expression and clinicopathological features in patients with ICC and found that high expression of circMBOAT2 was positively associated with tumor size in ICC patients (Table 1). Kaplan–Meier analysis indicated that higher circMBOAT2 expression levels were slightly correlated with a shorter OS in patients (*P* = 0.055) (Fig. 1E).

**Table 1:** Correlation between circMBOAT2 expression and clinical features in ICC.

Characteristics	The number of patients	gene expression		*P*-value
		Lower	Higher	
		(*n* = 13)	(*n* = 14)	
*Age*				0.436
≤60	16	7	9	
>60	11	6	5	
*Gender*				0.28
Male	14	8	6	
Female	13	5	8	
*Tumor size*				0.016
<5 cm	5	5	0	
≥5 cm	22	8	14	
*Biliary calculi*				0.5
No	23	12	11	
Yes	3	1	2	
*Hepatitis*				0.56
No	23	11	12	
Yes	3	1	2	
*Parasitic infection*				0.11
No	23	13	10	
Yes	3	0	3	
*Degree of pathological differentiation*				0.481
Moderate and high differentiation	26	12	14	
Poor differentiation	1	1	0	
*Lymph node metastasis*				0.529
No	25	12	12	
Yes	3	1	2	
*TNM stage*				0.546
I–II	20	10	10	
III–IV	7	3	4	
*Adjacent organ invasion*				0.538
No	22	11	11	
Yes	5	2	3	

CircMBOAT2 is generated from exons 2 to 3 of MBOAT2 with a length of 224nt. The backsplice junction site and full-length circMBOAT2 were amplified using divergent and convergent primers, then confirmed by Sanger sequencing (Fig. 1F). The sequence was maintained with the circBase database annotation (http://www.circbase.org/). Polymerase chain reaction (PCR) analysis showed that circMBOAT2 could be amplified using divergent primers in gDNA, cDNA reverse transcribed from random hexamers, and oligo(dT) primers. However, circMBOAT2 could only be amplified by convergent primers in cDNA reverse-transcribed from random hexamers instead of gDNA or cDNA reverse-transcribed from oligo(dT) primers (Fig. 1G). Resistance to digestion with RNase R exonuclease demonstrated that circMBOAT2 harbors a closed-loop structure (Fig. 1H). Nuclear and cytoplasmic fractionation and fluorescence in situ hybridization (FISH) revealed that circMBOAT2 was localized in both the cytoplasm and nucleus (Fig. 1I, J). A negative control probe was used to exclude non-specific staining during FISH (Fig. S2A). These results demonstrated that circMBOAT2 is a bona fide circRNA that is abundantly distributed in ICC.

### CircMBOAT2 promotes ICC progression in vivo and in vitro

To study the functional role of circMBOAT2 in ICC progression, we measured the endogenous expression of circMBOAT2 in seven cell lines. The results showed that the expression of circMBOAT2 is higher in QBC-939, HCCC-9810, RBE, and CCLP1, as well as FRH cells, than in H69 cells, a type of normal biliary epithelial cell (Fig. 1D). RBE and HCCC-9810 cells were used in the following experiments because they are universally acknowledged and the only two cell lines reserved in the ATCC cell repository. Furthermore, we used double sets of small interfering RNAs (siRNA) si-circMBOAT2#1 and si-circMBOAT2#2, specifically targeting the junction site of circMBOAT2, which significantly reduced the expression of circMBOAT2 instead of MBOAT2 mRNA in RBE and HCCC-9810 cells (Fig. S2B, C). Therefore, si-circMBOAT2#1 and si-circMBOAT2#2 were used for loss-of-function assays of circMBOAT2. To overexpress circMBOAT2, we constructed a circMBOAT2 overexpression plasmid and confirmed that circMBOAT2 was overexpressed accurately and efficiently in ICC cells (Fig. S2D, E).

**Fig. 2:**
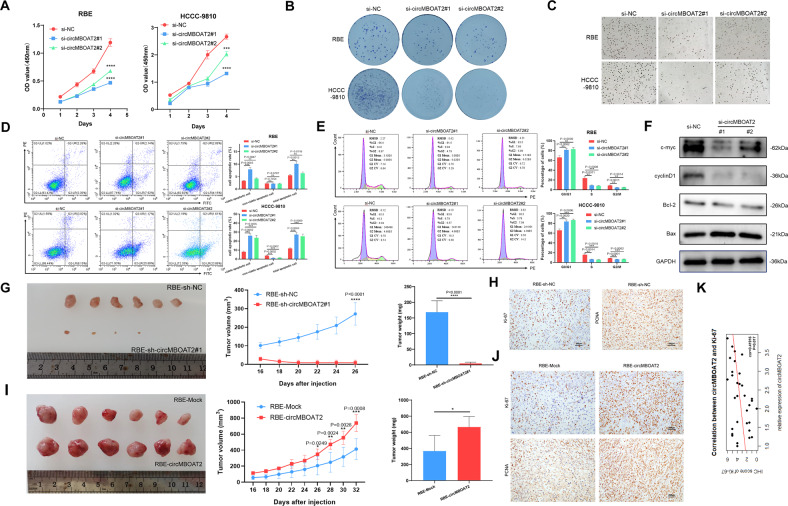
CircMBOAT2 promotes ICC progression in vitro and in vivo. **A** CCK8 assay for cell proliferation capacity. The results showed that the downregulation of circMBOAT2 inhibited the viability of RBE and HCCC-9810 cells. **B** Clone formation assay. The results showed that silencing circMBOAT2 inhibited the proliferative capacity of RBE and HCCC-9810 cells. **C** 5-Ethynyl-2’-deoxyuridine (EdU) proliferation assay. Knockdown of circMBOAT2 inhibits DNA synthesis in RBE and HCCC-9810 cells. The original magnification was 200×. **D** Data are expressed as the early and late stages of apoptosis rate after si-NC, si-circMBOAT2#1, or si-MBOAT2#2 transfection of RBE and HCCC-9810 cells. **E** The percentage cell phase distribution including G0/G1, S, and G2/M phases after transfection of RBE and HCCC-9810 cells with si-NC, si-circMBOAT2#1 or si-MBOAT2#2. **F** The expression of ICC progression-related proteins in circMBOAT2-knockdown ICC cells was detected by western blot. **G**, **I** The volume and weight of subcutaneous xenograft tumors (*n* = 6 mice per group). **H**, **J** The expression of Ki67 and PCNA by IHC staining. **K** Correlation between the expression of circMBOAT2 and Ki67 by Pearson correlation analysis. The original magnification was 200×. Significant differences between groups were analyzed by *t*-test. Error bars represent the means ± SEM of three independent experiments.

The biological functions of the knockdown and overexpression lines were further measured using the Cell Counting Kit-8 (CCK-8), colony formation, EdU cell proliferation assay, and flow cytometry. These results revealed that circMBOAT2 knockdown inhibited cell proliferation and colony formation but promoted both the early and late stages of apoptosis and G0/G1 cell cycle arrest in RBE and HCCC-9810 cells (Fig. 2A–E). However, these activities were contrary to those of circMBOAT2 knockdown when circMBOAT2 was overexpressed in RBE cells (Fig. S3A–D). To further characterize the mechanism of inhibiting cell proliferation, apoptosis, and G0/G1 cell cycle arrest by circMBOAT2 knockdown, we examined the expression levels of the key regulators by western blot. CircMBOAT2 knockdown markedly reduced the protein levels of c-myc, Bcl-2, and cyclin D1 and increased the level of Bax (Fig. 2F).

To evaluate the biological function of circMBOAT2 in vivo, a xenograft tumor model was constructed by inoculating different clones of RBE cells subcutaneously into nude mice. The results confirmed that tumor growth was significantly inhibited after interfering with the expression of circMBOAT2 (Fig. 2G). On the contrary, the mean tumor volumes and weights were larger, and tumor growth was rapid in the upregulated expression of the circMBOAT2 group, compared with the control group (Fig. 2I). Owing to the small size of tumor tissue in the sh-circMBOAT2 group, we took material from the other 3 groups and performed IHC staining, which revealed an increased proportion of proliferating cells (Ki67+ and PCNA+) in the upregulated expression of circMBOAT2 group compared with the control group (Fig. 2H, J). Moreover, we found a significant correlation between the expression of circMBOAT2 and Ki-67 in 32 tissues from ICC patients (Fig. 2K). Taken together, these findings indicated that circMBOAT2 promotes ICC growth.

### CircMBOAT2 regulates lipid metabolism reprogramming, especially unsaturated lipid

**Fig. 3:**
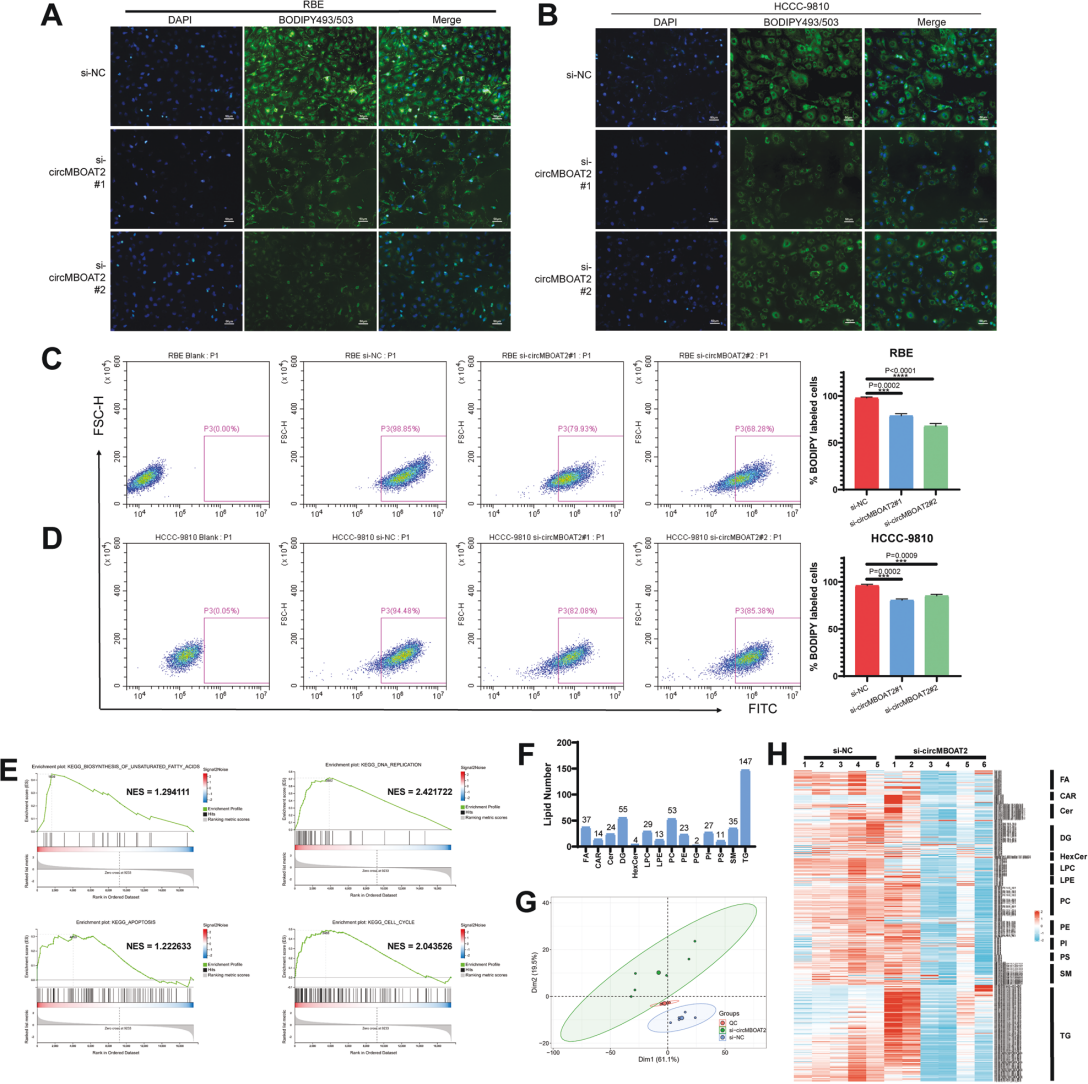
CircMBOAT2 regulates lipid metabolism reprogramming. **A**, **B** The neutral lipid droplets were detected by staining with BODIPY 493/503 in RBE and HCCC-9810 cells transfected by si-NC, si-circMBOAT2#1 and si-MBOAT2#2. Nuclei were stained with DAPI. Scale bar = 50 μm. **C**, **D** Flow cytometry to detect the neutral lipid droplets stained with BODIPY 493/503 in RBE and HCCC-9810 cells. FITC-A was used to detect BODIPY 493/503. **E** The circMBOAT2 knockdown group was compared with the control group for GSEA analysis. NES normalized enrichment score. **F** Lipid species identified in lipidomics analysis, HCCC-9810 cells transfected with si-NC or si-circMBOAT2#1. FA fatty acids; Car carnitines, Cer ceramides, DG diradylglycerolipids, HexCer hexceramides, LPC lysophosphatidylcholines, LPE lysophosphatidylethanolamines, PC phosphatidylcholines, PE phosphatidylethanolamines, PG phosphatidylglycerols, PI phosphatidylinositols, PS phosphatidylserines, SM sphingomyelins, TG triradylglycerolipids. **G** HCCC-9810 cells were transfected with si-NC or si-circMBOAT2#1 for lipid principal component analysis (PCA). **H** Heat map of changes in lipid species.

Cause circMBOAT2 expression is related to lipid metabolism from the analysis results shown in RNA-seq, we used the BODIPY493/503 probe to detect the neutral lipid droplet content in ICC cells. The results indicated that the neutral lipid droplet content was positively correlated with the expression of circMBOAT2 (Figs. 3A, B, S3E). This was further confirmed by flow cytometry (Figs. 3C, D, S3F). We then performed RNA-seq and untargeted lipid metabolomics (lipidomics) analysis of circMBOAT2-knockdown cells to validate our findings that circMBOAT2 modulates lipid metabolism reprogramming in ICC. Gene set enrichment analysis (GSEA) showed that the biosynthesis of unsaturated fatty acid pathway-related genes was affected in HCCC-9810 cells with circMBOAT2 KD (Fig. 3E). In the GSEA analysis, we also found that genes related to DNA replication, apoptosis, and cell cycle were also associated with circMBOAT2, which further proofed that circMBOAT2 promotes ICC growth (Fig. 3E). Untargeted lipidomics identified 474 lipids belonging to 14 classes (Supplementary Table 1, Fig. 3F). Principal component analysis (PCA) results showed that the lipidomics of HCCC-9810 cells with circMBOAT2 KD was quite different from that of negative control-transfected cells (Fig. 3G). The altered lipid species in circMBOAT2 knockdown HCCC-9810 cells are shown (Fig. 3H), and unsaturated lipids showed an obvious change compared to saturated lipids. Therefore, our data suggested that circMBOAT2 promotes ICC lipid metabolism reprogramming, especially unsaturated lipid metabolism reprogramming, which could be a characteristic of ICC progression.

### CircMBOAT2 interacts with PTBP1 in ICC cells

**Fig. 4:**
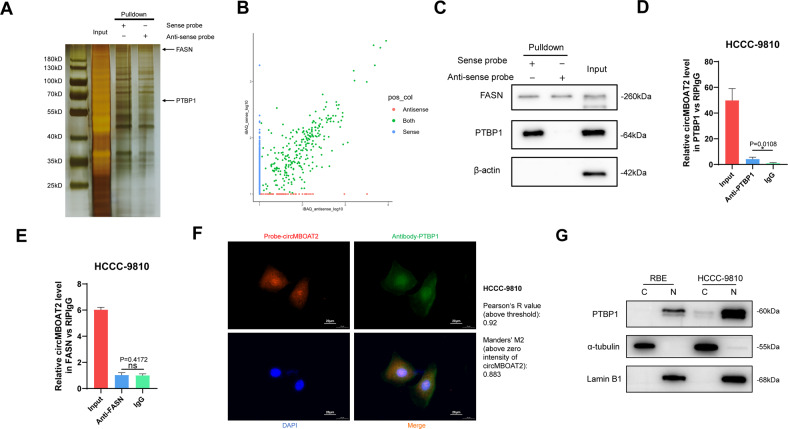
CircMBOAT2 interacts with PTBP1 in ICC cells. **A** RNA pull-down assay was performed; SDS-polyacrylamide gel electrophoresis (SDS/PAGE) and silver staining were used to detect RNA-related proteins. **B** Label-free intensity-based absolute quantification (iBAQ) method to quantify conjugated protein abundance with sense probe and anti-sense probe. **C** Western blot analysis was performed to detect the specific association of circMBOAT2 and PTBP1/FASN. β-actin was used as a negative control. **D, E** RNA immunoprecipitation (RIP) assay. CircMBOAT2 was precipitated by an anti-PTBP1 antibody specifically but an anti-FASN antibody nonspecifically was then detected by qRT-PCR in HCCC-9810 cells. IgG was used as a negative control. Significant differences between groups were analyzed by unpaired t-test. Error bars represent the means ± SEM of three independent experiments. **F** Immunofluorescence detected the co-localization of PTBP1 (green) with circMBOAT2 (red) in HCCC-9810 cells. **G** Subcellular localization of PTBP1. N nucleus, C cytoplasma. Scale bar = 20 μm. Analysis of colocalization was performed by ImageJ.

To test whether circMBOAT2 regulates downstream functions as a miRNA sponge in ICC, we conducted an RNA immunoprecipitation (RIP) assay. The results showed that circMBOAT2 was not significantly enriched by the AGO2 antibody (Fig. S4A, B), suggesting that circMBOAT2 may not act as a miRNA sponge during ICC progression. We then examined whether circMBOAT2 could be translated into a protein via an online database (http://lilab.research.bcm.edu/). The results showed that circMBOAT2 had an extremely low potential for coding (Fig. S4C). To explore whether circMBOAT2 fulfilled its function by interacting with proteins, we conducted an RNA pull-down assay to explore the proteins associated with it. The precipitated proteins in the RNA pull-down assay were separated using 10% sodium dodecyl sulfate-polyacrylamide gel electrophoresis (SDS-PAGE) and detected by silver staining (Fig. 4A). Liquid chromatography–mass spectrometry (LC-MS/MS) was used to identify the proteins pulled down by the circMBOAT2 probe, and the results are presented in Supplementary Table 2 and Fig. 4B. Among the proteins, FASN was found to be a lipid metabolism-associated protein that binds to both sense and antisense probes. Another RNA-binding protein, PTBP1, has been reported to regulate glycometabolism reprogramming in many sorts of cancers [26] and specifically interact with sense probes. Notably, AGO2 was not found in the precipitates, further confirming that circMBOAT2 does not function through the ceRNA mechanism. We performed western blot analysis, which was consistent with the LC-MS/MS results, PTBP1 and FASN were both bind with circMBOAT2 (Fig. 4C). Using the RIP assay, we confirmed that PTBP1 specifically binds to circMBOAT2 instead of FASN (Figs. 4D, E, S4D, E). In addition, we performed an RNA FISH immunofluorescence assay in the HCCC-9810 cell line and found that circMBOAT2 colocalized with PTBP1 in both the cytoplasm and nucleus (Fig. 4F). By co-localization analysis of ImageJ, it is calculated that the Pearson’s *R*-value (above threshold) is 0.92 and the Manders’ M2 (above zero intensity of circMBOAT2) is 0.883. Consistent with the subcellular localization of circMBOAT2, PTBP1 is also predominantly distributed in the nucleus (Fig. 4G). Taken together, these results indicated that circMBOAT2 functions by binding with PTBP1.

### CircMBOAT2 protects PTBP1 from ubiquitin/proteasome-dependent degradation

**Fig. 5:**
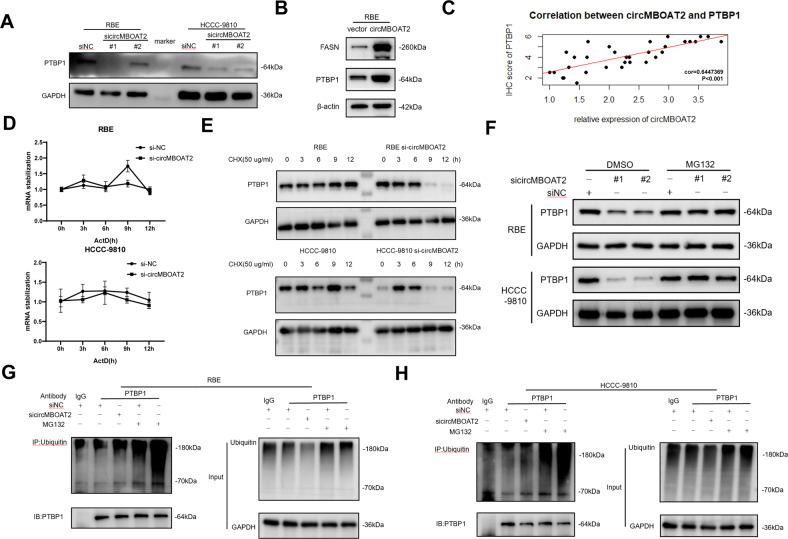
CircMBOAT2 protects PTBP1 from ubiquitin/proteasome-dependent degradation. **A**, **B** Protein levels of PTBP1 in RBE and HCCC-9810 cells with circMBOAT2 knockdown and overexpression. GAPDH or β-actin was used as a negative control. **C** Correlation between the expression of circMBOAT2 and PTBP1 protein by Pearson correlation analysis. **D** Analysis for RNA abundance of PTBP1 treated with Actinomycin D (2 μg/ml) at the indicated time point by qRT-PCR. **E** Analysis for protein abundance of PTBP1 treated with cycloheximide (CHX) (50 μg/ml) at the indicated time point by western blot. **F** CircMBOAT2 knockdown and control RBE and HCCC-9810 cells were incubated with MG132 (10 μM) for 8 h. Protein levels of PTBP1 were measured by western blot. **G**, **H** Immunoprecipitation detected ubiquitination modification of PTBP1 with or without MG132 treatment in RBE and HCCC-9810 cells. IgG was used as a negative control. Ub ubiquitin.

Western blot analysis of RBE and HCCC-9810 cells was performed to further examine the relationship between circMBOAT2 and PTBP1. Results showed that circMBOAT2 knockdown reduces PTBP1 protein level, and on the contrary, circMBOAT2 overexpression increases PTBP1 protein level (Fig. 5A, B). Through IHC, we found a significant correlation between the expression of circMBOAT2 and PTBP1 in 32 tissues from ICC patients (Fig. 5C). Notably, FASN protein levels increased under circMBOAT2 overexpression, indicating that FASN may be regulated in an indirect manner (Fig. 5B). We then performed PCR and western blot analysis in ICC cells to further examine the relationship between circMBOAT2 and PTBP1. To confirm that circMBOAT2 does not affect PTBP1 RNA stability, we performed RNA polymerase II Inhibitor Actinomycin (ActD) chase assays. Results showed that PTBP1 mRNA levels are not significantly altered in circMBOAT2-knockdown cells (Fig. 5D). In contrast, following treatment with the protein synthesis inhibitor cycloheximide (CHX), we found that circMBOAT2 knockdown increases the half-life of PTBP1 protein (Fig. 5E). Therefore, we speculated that circMBOAT2 may stabilize the PTBP1 protein through interaction.

Ubiquitin/proteasome-dependent degradation is the most common degradation pathway in eukaryotes [27]. Hence, we investigated whether circMBOAT2 knockdown significantly reduced PTBP1 protein levels, which could be restored by MG132, a specific proteasome inhibitor (Fig. 5F). Furthermore, an immunoprecipitation assay revealed that circMBOAT2 knockdown significantly increased the ubiquitination levels of PTBP1 (Fig. 5G, H). Collectively, these results demonstrated that circMBOAT2 increases the stability of PTBP1 by protecting it from ubiquitin/proteasome-dependent degradation.

### CircMBOAT2 promotes PTBP1-mediated cytoplasmic export of FASN mRNA

**Fig. 6:**
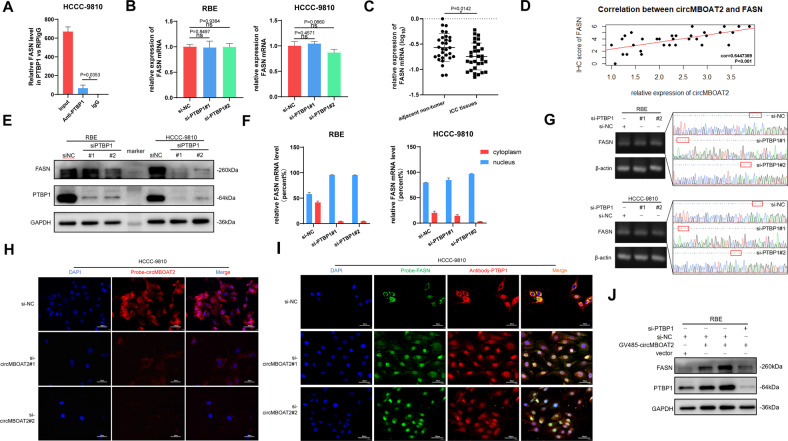
CircMBOAT2 promotes PTBP1-mediated cytoplasmic export of FASN mRNA. **A** RIP assay. FASN mRNA was precipitated by an anti-PTBP1 antibody specifically and detected by qRT-PCR in HCCC-9810 cells. IgG was used as a negative control. **B** The mRNA expression of FASN was determined with qRT-PCR, which was normalized to β-actin after PTBP1-knockdown in RBE and HCCC-9810 cells. **C** The qRT-PCR method was applied to detect the mRNA levels of FASN in 27 ICC patients and normal tissues adjacent to cancer. The expression of FASN was normalized to β-actin. Significant differences between groups were analyzed by paired sample *t*-test. **D** Correlation between the expression of circMBOAT2 and FASN protein by Pearson correlation analysis. **E** Western blot showed the protein levels of PTBP1 after PTBP1 knockdown in RBE and HCCC-9810 cells. GAPDH was used as a negative control. **F** The nuclear and cytoplasmic fractions were obtained by isolation. The qRT-PCR method was used to detect the mRNA levels of FASN in nuclear and cytoplasmic fractions of RBE and HCCC-9810 cells transfected with si-NC, si-PTBP1#1 or si-PTBP1#2. **G** PCR and Sanger sequencing for RBE and HCCC-9810 cells after PTBP1 knockdown. **H**, **I** The relative protein levels of PTBP1 and mRNA levels of FASN and the relative levels of circMBOAT2 after PTBP1 knockdown in HCCC-9810 cells were detected by immunofluorescence or FISH assays. Scale bar = 50 μm. **J** The protein levels of PTBP1 and FASN were determined by western blot analysis after PTBP1 knockdown and circMBOAT2 overexpression in RBE cells. Error bars represent the means ± SEM of three independent experiments.

As a sort of RBP, previous studies have shown that PTBP1 could mediate RNA splicing, and act as a regulator of glycolysis and tumorigenesis [28]. The above results showed that the FASN protein level was increased under circMBOAT2 overexpression (Fig. 5B), as a consequence of which, we inspected that PTBP1 could bind with FASN mRNA and then regulate its expression. By performing the RIP assay, we confirmed that PTBP1 could bind to FASN mRNA (Fig. 6A). To investigate the function of PTBP1 after binding with FASN mRNA, we detected the level of FASN mRNA in the presence of PTBP1 knockdown using two sets of siRNAs (si-PTBP1#1 and si-PTBP1#2) and found that there was no significant difference after PTBP1 knockdown (Fig. 6B). This indicated that PTBP1 does not affect the stability of FASN mRNA. Coincident with the performance in ICC cell lines, compared to adjacent non-tumor tissues, the RNA level of FASN was not significantly different between ICC tissues and normal tissues adjacent to ICC tissues (Fig. 6C). However, the expression level of FASN protein was significantly correlated with circMBOAT2 (Fig. 6D). The protein level of FASN was decreased after PTBP1 knockdown in RBE and HCCC-9810 cells (Fig. 6E). In order to investigate whether PTBP1 mediates the alternative splicing of FASN mRNA, we searched for the splicing form of FASN from NCBI and designed primers containing the splice region. Following Sanger sequencing, we found that the splice form of FASN was not changed after knocking down PTBP1 (Fig. 6G). Therefore, we speculated that PTBP1 could promote cytoplasmic export of FASN mRNA. To test this hypothesis, nuclear and cytoplasmic fractionation and FISH were performed. Interestingly, we observed a clear reduction in cytoplasmic FASN mRNA levels in RBE and HCCC-9810 cells upon PTBP1 knockdown (Fig. 6F, H, I). We further investigated the effect of PTBP1 knockdown on FASN expression. PTBP1 knockdown abrogated the effect of circMBOAT2 overexpression on FASN expression in RBE (Fig. 6J). These results revealed that circMBOAT2 promotes cytoplasmic export and expression of FASN mRNA by interacting with PTBP1 in ICC cells.

### Arachidonic acid (C20:4) and adrenic acid (C22:4) intervene ICC progression in circMBOAT2/PTBP1/FASN axis

**Fig. 7:**
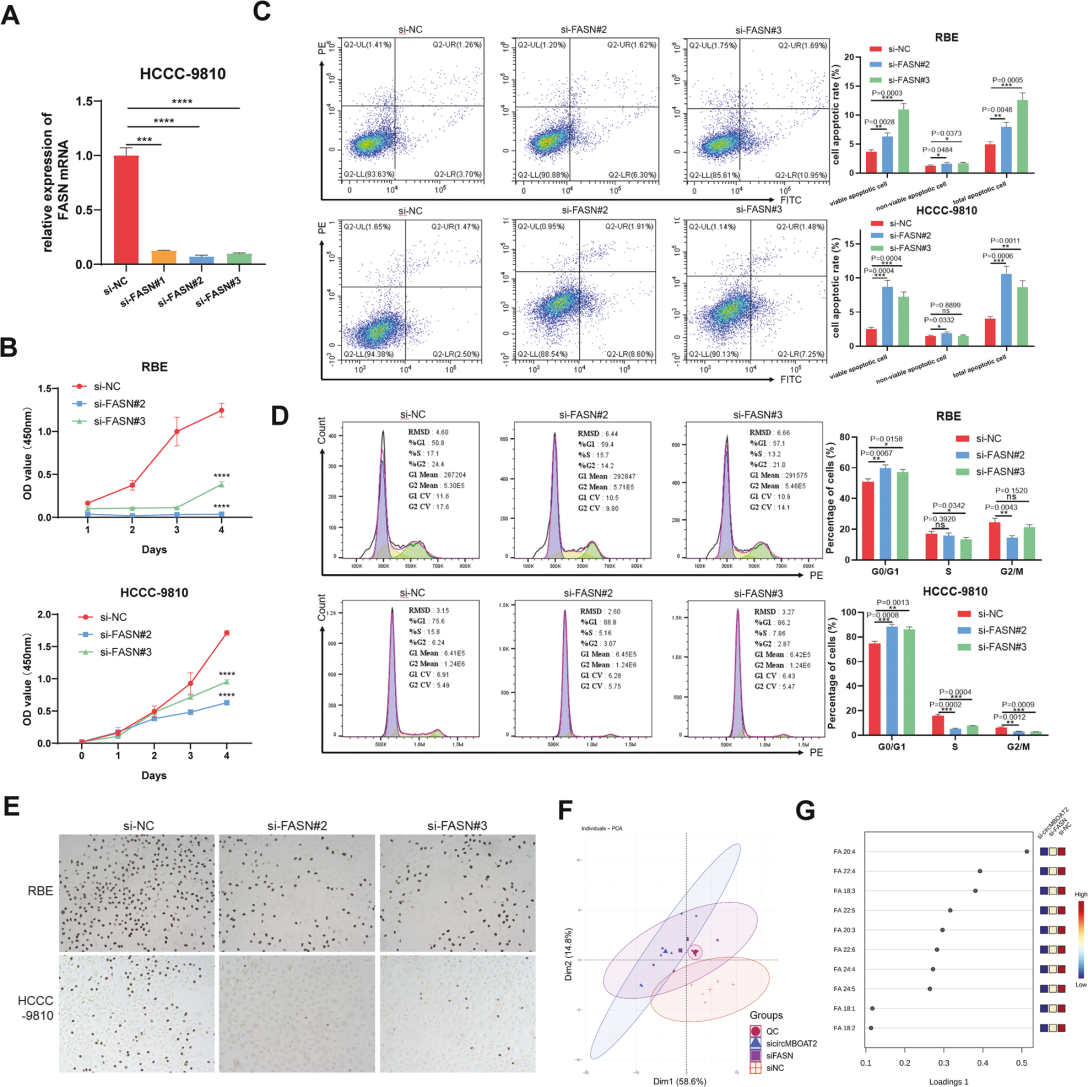
Arachidonic acid (C20:4) and adrenic acid (C22:4) intervene in ICC progression in circMBOAT2/PTBP1/FASN axis. **A** The qRT-PCR method was applied to detect the mRNA levels of FASN after transfected with si-NC, si-FASN#1, si-FASN#2, and si-FASN#3. The expression of FASN was normalized to β-actin. **B** CCK8 assay for cell proliferation capacity after FASN-knockdown in RBE and HCCC-9810 cells. **C** Flow Cytometry was applied to determine the early and late stages of apoptosis rate after FASN-knockdown in RBE and HCCC-9810 cells. **D** Flow cytometry was applied to determine the percentage cell phase distribution, including G0/G1, S, and G2/M phases after FASN-knockdown in RBE and HCCC-9810 cells. **E** EdU proliferation assay was used to detect the levels of DNA synthesis in RBE and HCCC-9810 cells after FASN knockdown. The original magnification was 200×. **F** HCCC-9810 cells were transfected with si-NC, si-circMBOAT2#1, or si-FASN#2 for lipid principal component analysis (PCA). **G** VIP scores plot in loadings 1 of PLS-DA. Significant differences between groups were analyzed by *t*-test. Error bars represent the means ± SEM of three independent experiments.

We then investigated whether the role of circMBOAT2 in ICC progression is dependent on the FASN pathway. The efficiency of three sets of siRNAs, si-FASN#1, si-FASN#2, and si-FASN#3, was tested, all of which could reduce the expression of FASN **(**Fig. 7A). Si-FASN#2 and si-FASN#3, which were more efficient, were used in subsequent assays. In vitro assays using the CCK-8, EdU cell proliferation assays, and flow cytometry demonstrated that reduced expression of FASN functionally inhibited cell proliferation, promoted both the early and late stages of apoptosis and G0/G1 cell cycle arrest in RBE and HCCC-9810 cells (Fig. 7B–E). To further prove the role of FASN in ICC progression, a FASN-suppressive pharmaceutical, TVB-2640(Denifanstat), was used. Consistent with the results of functional assays using siRNA, TVB-2640 inhibited cell proliferation and promoted both early and late stages of apoptosis and G0/G1 cell cycle arrest in HCCC-9810 cells (Fig. S5A–C).

We next compared the lipid levels among control, circMBOAT2-silenced, or FASN-silenced ICC cells by lipidomics. PCA analysis indicated that the downregulation of FASN in ICC cells was parallel to circMBOAT2 silencing (Fig. 7F). The altered lipid species in circMBOAT2-silenced and FASN-silenced ICC cells are presented (Fig. S6A). In terms of fatty acids, semblable changes occurred in circMBOAT2-silenced or FASN-silenced ICC cells (Fig. S6B). Except for C16:0, no significant differences in saturated fatty acids were found, while unsaturated fatty acids were different, and the trend was more obvious in unsaturated fatty acids with more unsaturated bonds or fewer carbons (Fig. S6C, D). These data suggested that the lipid metabolism reprogramming functions of circMBOAT2 in promoting ICC progression rely on the FASN pathway. The level of unsaturated fatty acids, especially polyunsaturated fatty acids, is thus significantly altered in ICC cells.

**Fig. 8:**
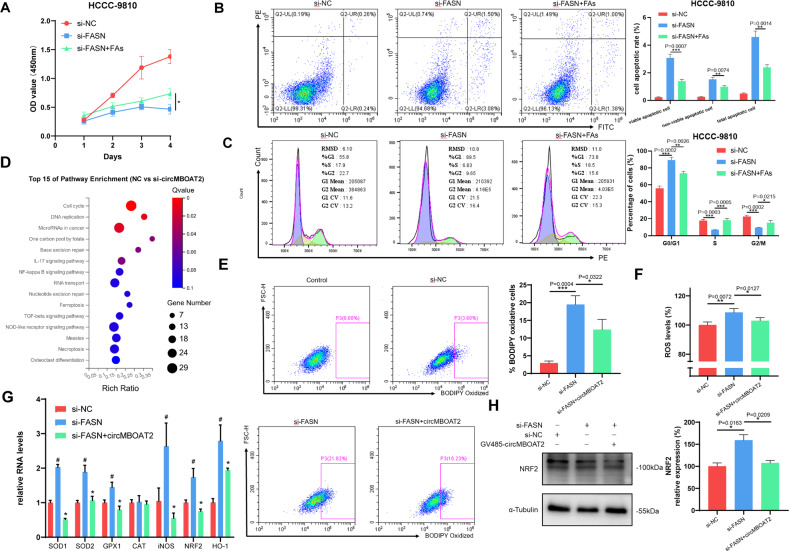
CircMBOAT2 reduces oxidative stress in ICC cells through FASN. **A** CCK8 assay for cell proliferation capacity after transfected with si-FASN and additional arachidonic acid (20 μM) and adrenic acid (10 μM) supplements in HCCC-9810 cells. **B** Flow Cytometry was applied to determine the early and late stages of apoptosis rate after transfected with si-FASN and additional arachidonic acid (20 μM) and adrenic acid (10 μM) supplements in HCCC-9810 cells. **C** Flow Cytometry was applied to determine the percentage cell phase distribution, including G0/G1, S, and G2/M phases after transfection of si-FASN and additional arachidonic (20 μM) acid and adrenic acid (10 μM) supplements in HCCC-9810 cells. **D** KEGG enrichment analysis for the top 15 pathways in the circMBOAT2 knockdown group compared with the control group by RNA sequencing. **E** Lipid peroxidation level present in HCCC-9810 cells. Cells were incubated with a BODIPY C11 probe for 30 min. The fluorescence inside the cells was analyzed using flow cytometry. **F** Intracellular ROS levels in HCCC-9810 cells by flow cytometry. Data were normalized to the controls, which were set as 100%. **G** The qRT-PCR method was applied to detect the mRNA levels of SOD1, SOD2, GPX1, CAT, iNOS, NRF2, and HO-1, which were normalized to β-actin. ∗ indicates the significant difference compared with the control group, and # indicates the significant difference compared with the si-FASN group. **H** NRF2 protein levels after FASN knockdown in HCCC-9810 cells. Significant differences between groups were analyzed by *t*-test. Error bars represent the means ± SEM of three independent experiments.

Polyunsaturated fatty acids (PUFAs) are essential for the formation of cell membrane phospholipids, which are required for the rapid proliferation of cancer cells [29]. It has been reported that intake of n-6 PUFAs is associated with an elevated proportion of eicosanoids with carcinogenic effects [30]. Based on partial least squares discriminant analysis (PLS-DA), we found that the proportion of two sorts of n-6 PUFAs, arachidonic acid (C20:4) and adrenic acid (C22:4), were significantly reduced in the circMBOAT2-silenced and FASN-silenced group (Fig. 7G), as a consequence of which, we explored the function of these two n-6 PUFAs in FASN-mediated ICC progression. In vitro, CCK-8, EdU cell proliferation assays, and flow cytometry demonstrated that reduced expression of FASN functionally inhibited cell proliferation and promoted both the early and late stages of apoptosis and G0/G1 cell cycle arrest in HCCC-9810 cells, which could be reversed by additional arachidonic acid and adrenic acid supplements (Fig. 8A–C). This tentatively indicates a possible role for arachidonic acid and adrenic acid in the carcinogenicity of FASN in ICC.

### CircMBOAT2 reduces oxidative stress in ICC cells through FASN

In order to further investigate signaling pathways involved in circMBOAT2, we checked the transcriptome profiling of RNA-seq in tissues and cells performed above. KEGG pathways enrichment analysis showed that circRNAs were related to the cytochrome P450 pathway (Fig. 1B). Consistently, circMBOAT2-KD triggered ferroptosis and NF-kappa B signaling pathway (Fig. 8D). These results indicated that circMBOAT2 is involved in regulating the redox homeostasis of ICC. Thus far, we examined the levels of lipid peroxidation and reactive oxygen species (ROS) in HCCC-9810 cells. Flow cytometry demonstrated that overexpression of circMBOAT2 inhibited FASN-KD-induced lipid peroxidation and ROS production (Fig. 8E, F), which explained the previous knockdown of FASN and circMBOAT2-induced apoptosis. The mRNA levels of superoxide dismutase (SOD1 and SOD2), glutathione peroxidase 1 (GPX1), inducible NO synthase (iNOS), nuclear factor erythroid 2-related factor 2 (NRF2), and hemeoxygenase-1 (HO-1) were significantly higher in FASN-KD cells and recovered when circMBOAT2 was overexpressed (Fig. 8G). Furthermore, overexpression of circMBOAT2 suppressed the FASN-KD-promoted expression of NRF2 in ICC cells (Fig. 8H).

**Fig. 9:**
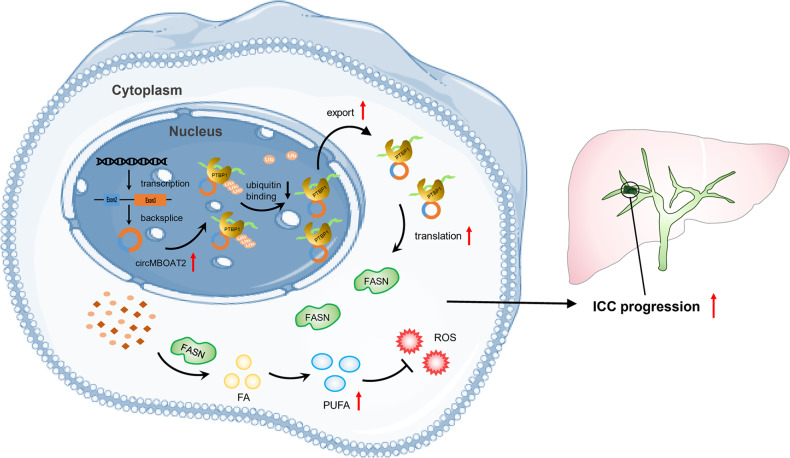
Proposed working model for promotional effects of circMBOAT2 on ICC progression. CircMBOAT2 derives from the exons 2–3 of the MBOAT2 gene by backsplicing in the nucleus and exporting to the cytoplasm. CircMBOAT2 stabilized PTBP1 to facilitate FASN mRNA cytoplasmic export, which altered the lipid metabolic profile and regulated redox homeostasis in ICC, thus promoting tumor growth.

Taken together, these results demonstrated that circMBOAT2 promotes lipid metabolism reprogramming of ICC, which is regulated by the circMBOAT2/PTBP1/FASN axis. The altered lipid metabolic profile affects cell membrane composition and energy metabolism along with redox homeostasis, all of which lead to the progression of ICC together (Fig. 9).

## Discussion

CircRNAs have been discovered for more than 45 years, however, they were considered accessory substances generated by abnormal splicing with little functional potential until 2013 [31, 32]. Increasing evidence suggests that circRNAs are dysregulated in multiple cancers, and their atypical function in regulating cancer cell proliferation, migration, invasion, and metastasis renders them potential biomarkers and therapeutic targets [31]. Aberrant regulation of circRNAs has also been proposed to be involved in clinical cancer treatment resistance, which includes standard chemotherapy, targeted therapy, and immunotherapy [33]. Nonetheless, the molecular mechanisms of circRNAs in ICC remain unclear. An earlier study showed that hsa_circ_0021205 suppresses ICC progression by sponging miR-204-5p [34]. Even more, interestingly, circGGNBP2 is able to encode a small non-featured peptide, cGGNBP2-184aa, and it is cGGNBP2-184aa rather than circGGNBP2 that promotes cell growth and metastasis in ICC [5]. In the present study, we performed whole-transcriptome sequencing and found that circMBOAT2 was frequently downregulated in ICC tissues. CircMBOAT2 not only directly binds to PTBP1 to prevent it from ubiquitinated degradation but also elicits ICC lipid metabolism reprogramming by facilitating FASN translation. We report for the first time that circMBOAT2 promotes ICC progression by stabilizing PTBP1 and activating the reprogramming of lipid metabolism.

Increasing evidence suggests that circRNA expression levels are related to the clinicopathological characteristics of tumor patients [33]. We found that a decrease in circMBOAT2 expression was significantly associated with tumor size in patients with ICC (Table 1). However, at the time of our study, we noted that the majority of patients (23/27) were hepatitis-free. The prevalence of hepatitis in our cohort differs from the typical circumstances of ICC: 27% of ICC patients in the U.S. have a hepatitis virus infection, and 22.5% have an HBV infection in China [1, 35]. Hence, whether circMBOAT2 expression is associated with hepatitis virus infection in ICC patients requires further investigation by expanding the sample size of the cohort. In addition, oncogenic driver mutations and gene rearrangements in ICC have gradually gained attention. IDH1/2 and FGFR mutations have been reported to occur at frequencies of 10–20% and 10–15% in ICC, respectively, and the corresponding targeted therapeutics, ivosidenib and pemigatinib, have been shown to be effective in clinical trials [36, 37]. However, the correlation between IDH1/2 and FGFR mutation status and circMBOAT2 is unclear and requires further investigation.

ICC is the second most common primary liver tumor, with limited therapeutic options. Despite advances in the comprehensive therapy of patients, the prognosis has not improved significantly over the past decades, with a 5-year survival rate of approximately 22–44% after resection [4]. Therefore, new strategies are urgently needed to improve the prognosis of ICC patients. Dysregulating cellular metabolism was segregated as “emerging hallmarks” in the Hallmarks of Cancer [17], which could be a new strategy for curing ICC. In our study, we successfully identified the metabolism-related circRNA circMBOAT2 from RNA-seq data of ICC tissues, which is one of the overexpressed circRNAs and has also been shown to correlate with tumor size in clinical specimens. In the following functional assays, circMBOAT2 significantly facilitated proliferation and colony formation in ICC cells but prevented both the early and late stages of apoptosis and G0/G1 cell cycle arrest. Based on the above findings, the oncogenic role of circMBOAT2 in ICC, especially the proliferative phenotype, was robustly confirmed.

Previous studies have focused on the role of circMBOAT2 as a miRNA sponge [38, 39]. In the present study, by performing an RNA pull-down assay and LC-MS/MS analysis, we demonstrated that circMBOAT2 does not exert its function through a ceRNA mechanism, but enhances the stability of PTBP1, a ribosomal protein, by binding to it. We subsequently investigated the relationship between circMBOAT2 and PTBP1 and found for the first time that cirMBOAT2 protects PTBP1 from degradation by reducing ubiquitin-mediated proteolysis, thereby enhancing its activity. However, it is unclear whether circMBOAT2 directly inhibits the ubiquitination of PTBP1 by competitively binding to a ubiquitin ligase recognition domain or acts as a scaffold that provides a platform for the interaction between PTBP1 and specific deubiquitination-associated proteins. Therefore, this question requires further investigation.

As a ribosomal protein, PTBP1 promotes the development of various types of cancer by enhancing the stability of mRNA or the translation of oncogenic factors [28]. However, little is known about the function of PTBP1 in the regulation of cytoplasmic export of RNA in cancer cells. Meanwhile, the role of PTBP1 in ICC has not been investigated. In the present study, we found that PTBP1 could bind to FASN mRNA, transport it to the cytoplasm, and then translate. The relationship between PTBP1 and tumor metabolism is mainly the ability to regulate the expression of PKM2 and promote glycolysis [26, 40]. However, the function that PTBP1 regulates lipid metabolism has not been reported yet. We first identified the critical role of PTBP1 in the regulation of cytoplasmic export of FASN mRNA and in the progression of ICC. This discovery enriches the literature that PTBP1 regulates RNA subcellular localization and fills a gap in the study of PTBP1 in ICC and the regulation of lipid metabolism.

It is widely perceived that fatty acids (FAs) are critical for cancer cells as they maintain membrane biosynthesis during rapid cell proliferation and supply an important source of energy under conditions of metabolic stress. In addition, FAs and their by-products have been found to act as secondary messengers for signal transduction or directly regulate intracellular homeostasis by modulating the surrounding microenvironment to create conditions favorable for tumor progression [41]. Therefore, suppression of FAs metabolic pathways is a reasonable option for tumor therapy. Notably, FASN plays an essential role in the de novo synthesis of FAs, of which palmitate (C16:0) is the major product and can generate additional FAs species through the prolongation and desaturation of SCDs, ELOVLs, and FADs. The conversion of the lipid synthesis transcriptional regulator SREBP1 to its mature active form is strongly affected by a PI3K-AKT-mTORC1-dependent mechanism; thus, the expression of pivotal lipogenic enzymes, such as FASN, is inhibited when mTORC1 is blocked by rapamycin or Raptor knockdown [42]. Nevertheless, the mechanism of the post-transcriptional regulation of FASN remains unknown. In the present study, we inhibited FASN by downregulating circMBOAT2 levels and observed similar effects to the direct knockdown of FASN in ICC proliferation and lipid metabolism. Hence, our findings support the hypothesis that circMBOAT2 is a promising target for cancer therapies. Remarkably, we found that the FASN-block effectively inhibited ICC progression in vitro, suggesting that it may have beneficial effects in patients with circMBOAT2-associated ICC (i.e., patients with elevated levels of circMBOAT2 expression). Importantly, although FASN-block-mediated inhibition of metabolic activity is consistent with that in circMBOAT2 knockdown ICC cells, its specific mechanism may be complicated and requires further study.

In our study, we also found that the FASN-block mostly affected the levels of unsaturated FAs (UFAs), especially polyunsaturated FAs (PUFAs) in ICC cells, while the levels of saturated FAs (SFAs) were less affected. PUFAs were previously reported to be preferentially incorporated and predominantly metabolized in colorectal cancer cells, with more PUFAs than in the normal intestinal mucosa [43], which is consistent with our findings. One of the probable reasons for this is that PUFAs are essential for the formation of cell membrane phospholipids during the rapid proliferation of cancer cells. Conversely, results from in vitro and in vivo studies concluded that PUFAs may have anti-cancer properties. The consumption of n-3 UFAs reduces the risk of various cancers, including breast [44], colon [45], prostate [46], leukemia [47], and melanoma [48]. The addition of n-3 PUFAs to the diet inhibits inflammatory processes, stimulates apoptosis, suppresses metastasis and tumor proliferation, and upregulates the expression of antioxidant enzymes. In contrast, the intake of n-6 PUFAs was associated with an increased proportion of eicosanoids, which were carcinogenic [29]. The explanation may be that n-3 and n-6 PUFAs have contrasting effects on the progression of cancer; however, their roles in ICC progression remain to be further investigated.

PUFAs are metabolized by oxygenation to produce a variety of lipid metabolites, collectively referred to as oxidized lipids or oxylipids. Oxylipids have an important regulatory role in the initiation, development, and regression of inflammation. Oxylipids also have a role in regulating intracellular redox state, in which some oxylipins promote the production of oxidants or are highly active oxidants themselves, while others have the ability to inhibit the production of pro-oxidants or promote the production of antioxidants [49]. The enzyme-mediated oxylipids synthesis pathway mainly includes cyclooxygenase (COX), lipoxygenase (LOX), and cytochrome P450 (CYP) epoxygenases [50]. Our results showed that circRNAs were related to the cytochrome P450 pathway. So we hypothesized that PUFAs were mainly catalyzed via the CYP pathway. Epoxyeicosatrienoic acid, produced with arachidonic acid as a substrate through the CYP pathway, has been reported to have anti-oxidative and antiapoptotic effects on endothelial cells and other cells [51]. Our results showed that FASN-KD upregulated lipid peroxidative and ROS in ICC, which was recovered by circMBOAT2 overexpression. This may be due to a reduction in EEA. In addition, nutritional stress due to reduced FAs after FASN-KD is a possible cause of enhanced oxidative stress.

In conclusion, our study is the first to suggest that lipid metabolic reprogramming in ICC is regulated by circMBOAT2, which is associated with its progression. Mechanistically, we found that circMBOAT2 bound to and stabilized PTBP1, thereby contributing to the cytoplasmic export of FASN mRNA. Upregulation of FASN altered lipid metabolic profile and regulated redox homeostasis in ICC. Importantly, our findings suggest that silencing circMBOAT2 offers a new therapeutic strategy for ICC treatment, especially in patients with active lipid metabolism.

## Materials and methods

### Patients and tissues

This study was approved by the Ethics Committee of Xinhua Hospital (Shanghai, China) and was conducted in accordance with the Declaration of Helsinki. Written informed consent was obtained from all patients before the study began.

Tumors and adjacent normal tissues were obtained from 27 patients who underwent radical resection between 2015 and 2017 at Xinhua Hospital. Each tissue sample was snap-frozen in liquid nitrogen for further analysis. All the patients in this study belonged to the same ethnic group. The patients were selected according to the following criteria: (1) All clinicopathological diagnoses were confirmed by two pathologists. (2) None of the patients had received any treatment before surgery. (4) Availability of complete follow-up data. (5) No death occurred during the perioperative period. (6) No history of other synchronous malignancies.

### RNA sequencing

Total RNA from tissues and cells was isolated using the Hipure Total RNA Mini Kit (Magen), according to the manufacturer’s protocol. RNAs were eluted with 50 μl of RNase-free water, and the concentration and integrity of the extracted total RNA were estimated with a Qubit 3.0 Fluorometer (Invitrogen, Carlsbad, California) and Agilent 2100 Bioanalyzer (Applied Biosystems, Carlsbad, CA), respectively. RNA samples with a RIN value of at least 7.0 or higher were used for further processing. The RNA-seq library was prepared with approximately 1 μg of total RNA using the KAPA Stranded RNA-Seq Kit with RiboErase (HMR) for Illumina Platforms (Kapa Biosystems, Inc., Woburn, MA, USA). Briefly, ribosomal RNA was removed from the total RNA. Next, first-strand and directional second-strand syntheses were performed. Then, tailing and adapter ligation were performed using the purified cDNA. Finally, the purified adapter-ligated DNA was amplified. Library quality and concentration were assessed using a DNA 1000 chip on an Agilent 2100 bioanalyzer. Accurate quantification for sequencing applications was performed using a qPCR-based KAPA Biosystems Library Quantification Kit (Kapa Biosystems, Inc., Woburn, MA, USA). Each library was diluted to a final concentration of 10 nM and pooled to equimolar concentrations prior to clustering. Paired-end (PE) sequencing was performed on all samples. For circRNA expression analysis, the read was mapped to the genome using STAR, and DCC was used to identify the circRNAs and estimate circRNA expression. The trimmed mean of *M*-values (TMM) was used to normalize gene expression. Differentially expressed genes (DEGs) were identified using EdgeR.

### Cell cultures

Human cholangiocarcinoma cell lines (RBE, HCCC-9810, HuCCT1, CCLP1, and QBC-939) and a human normal bile duct epithelial cell line (H69) were obtained from the Cell Bank of the Shanghai Cell Bank of the Chinese Academy of Sciences (Shanghai, China). Cells were maintained at 37 °C in a 5% CO_2_ humidified incubator and cultured in RPMI-1640 (Gibco) (H69, RBE, HuCCT1, CCLP1, and QBC-939) or DMEM (Gibco) (HCCC-9810) supplemented with 10% fetal bovine serum (Gibco) and 1% antibiotics(Gibco). The cells were not cultured for longer than 2 months.

### RNAi and plasmid construction

siRNA duplexes were synthesized by GenePharma (Shanghai, China) and transfected into cells using Lipofectamine 3000 (Invitrogen), according to the manufacturer’s protocol. Lentiviruses for the knockdown of circMBOAT2 and a plasmid for circMBOAT2 overexpression were obtained from GeneChem (Shanghai, China). The target sequences for constructing the lentiviral shRNAs and siRNAs are listed in Additional File 2.

### RNA extraction and qRT-PCR analysis

Total RNA derived from ICC tissues and cells was extracted using TRIzol reagent (TaKaRa, Dalian, China), according to the manufacturer’s instructions. RNA was reverse-transcribed into cDNA using a Primer-Script one-step RT-PCR kit (TaKaRa, Dalian, China). The Hieff UNICON^®^ qPCR SYBR^®^ Green Master Mix (Yeasen, Shanghai, China) was used for qRT-PCR. The circRNA and mRNA levels were normalized by GAPDH, β-actin, or U3. The fold change in the relative expression of RNAs was calculated using the 2^−ΔΔCt^ method. The oligonucleotide sequences are listed in Additional File 2.

### RNase R treatment

Two micrograms of total RNA were incubated for 30 min at 37 °C in the absence or presence of 5 U/μg RNase R (Geneseed, Guangzhou, China), and the resulting RNA was subsequently purified the RNeasy Mini Kit (Qiagen, Germany) and then analyzed by qRT-PCR.

### RNA fluorescence in situ hybridization (FISH)

Oligonucleotide-modified probe sequences for circMBOAT2 and FASN were synthesized by Sangon Biotech (Shanghai, China). The fixed cells were then washed with PBS. The cell suspension was pipetted onto autoclaved glass slides, followed by dehydration with 70, 80%, and 100% ethanol. Then hybridization was performed at 37 °C overnight in a dark moist chamber. After washing twice in 50% formamide/2× SSC for 5 min, the slices were incubated with the reagents in Alexa FluorTM 488 Tyramide SuperBoost™ Kits (Thermo Fisher Scientific, Waltham, USA) for 30 min and sealed with parafilm containing DAPI. Images were acquired using a fluorescence microscope (OLYMPUS, Tokyo, Japan). The probe sequences were as follows:

circMBOAT2: 5′-Cy3-CACTACAAAGTTGACTTGTGCATGTTCTCCACT-3′

FASN: 5′-digoxin-GCGTAGGATGGAATCTCGGAAGCGGTC-3′

### In vitro cell phenotypic assays

For the CCK-8 proliferation assay, 2 × 10^3^ cells were seeded in 100 μl complete culture media in 96-well plates for various time periods. The Cell Counting Kit-8 assay was performed to measure cell viability, according to the manufacturer’s instructions. For the 5-Ethynyl-2′-deoxyuridine (EdU) proliferation assay, BeyoClick^TM^ EdU proliferation assay (Beyotime, Shanghai, China) was used according to the manufacturer’s protocol. The cells were incubated with 10 μM EdU for 2 h, stained with DAB, and visualized under a light microscope.

For cell cycle analysis, after 48 h of incubation, transfected ICC cells were washed with cold phosphate-buffered saline (PBS) and incubated in ice-cold 70% ethanol at 4 °C overnight. Cells were then incubated with propidium iodide and Rnase A for 30 min, and the cell cycle distribution was analyzed using a flow cytometer (FACS Calibur, BD Biosciences, USA). Data were analyzed using FlowJo 10.6.2 software and presented as the percentage of cell phase distribution, including the G0/G1, S, and G2/M phases. For cell apoptosis analysis, cultured cells were digested by trypsin and washed with PBS, then stained with annexin V-fluorescein isothiocyanate and propidium iodide for flow cytometric analysis (BD Biosciences, USA).

### Tumor xenograft models

The animal experiments were approved by the Ethics Committee of Xinhua Hospital, Affiliated with Shanghai Jiao Tong University School of Medicine, and carried out in accordance with its guidelines. Six mice were randomly assigned to each group, as reported before [52]. Male BALB/c nude mice (4–6 weeks old) were housed in a specific pathogen-free animal room under 12-h light-dark cycles, with water and food available. To establish a subcutaneous ICC model, 1 × 10^6^ RBE cells in 0.1 ml of PBS were subcutaneously injected into the flanks. After 4–5 weeks of feeding, the animals were euthanized, and the subcutaneous tumors were removed for further research.

### Immunohistochemistry analysis

Immunohistochemistry (IHC) staining was performed following a standard procedure. Tumor tissues were fixed in 4% paraformaldehyde, embedded in paraffin, and sectioned. Slides were dewaxed in xylene and rehydrated in reduced concentrations of ethanol. 3% hydrogen peroxide blocked endogenous peroxidase activity. The slides were then incubated with primary antibodies against ki67 (27309-1-AP, Proteintech Group, USA), PCNA (10205-2-AP, Proteintech Group, USA), PTBP1 (12582-1-AP, Proteintech Group, USA) and FASN (10624-2-AP, Proteintech Group, USA) at 4 °C. The following day, they were incubated with enzyme-labeled secondary antibodies. Finally, the slides were developed with diaminobenzidine and counterstained with hematoxylin.

Sections were semiquantitatively scored [53]. The percentage of positive staining cells was scored as follows: 0 for no staining, 1 for <5% immunoreactive cells; 2 for 5–50% immunoreactive cells; and 3 for >50% immunoreactive cells. In addition, the staining intensity was graded as 0 for no staining, 1 for weak immunoreactivity, 2 for intermediate immunoreactivity, and 3 for strong immunoreactivity. The samples were grouped based on the sum of both extension and intensity parameters: negative (0), weak (1–2), moderate (3), and strong (4–6) staining.

### Immunofluorescence assays

The cultured cells were fixed using 4% paraformaldehyde for 15 min at least. The samples were then incubated with primary antibody against PTBP1 (12582-1-AP, Proteintech Group, USA) at 4 °C overnight and then incubated with goat anti-rabbit IgG with a red or green fluorescent label (Invitrogen, Carlsbad, CA). After washing twice in PBS, the slices were sealed with parafilm containing DAPI. Images were acquired using a fluorescence microscope (OLYMPUS, Tokyo, Japan).

### Western blot

Proteins were extracted from transfected cells using RIPA lysis buffer. Equal amounts of protein samples were loaded and separated by SDS-PAGE and transferred onto PVDF membranes (Merck Millipore, Germany). Membranes were blocked with 5% skim milk in TBST for 1 h at room temperature. Then, Membranes were incubated with diluted primary antibodies anti-PTBP1 for western blot (1:1000 dilution, 12582-1-AP, Proteintech Group, USA), anti-FASN for western blot (1:1000 dilution, 10624-2-AP, Proteintech Group, USA), anti-c-myc for western blot (1:1000 dilution, ab32072, Abcam, UK), anti-Bcl-2 for western blot (1:1000 dilution, 4223, Cell Signaling Technology, USA), anti-cyclin D1 for western blot (1:1000 dilution, 55506, Cell Signaling Technology, USA), anti-Bax for western blot (1:1000 dilution, 5023, Cell Signaling Technology, USA), anti-NRF2 for western blot (1:1000 dilution, 16396-1-AP, Proteintech Group, USA), anti-laminB1 (1:1000 dilution, 66095-1-Ig, Proteintech Group, USA) for western blot at 4 °C overnight. Then, membranes were washed with TBST three times for 15 min/wash, followed by incubation with a secondary antibody (Beyotime, Shanghai, China) for an hour, and washed again with TBST. Finally, the protein bands were visualized using Gel Doc 2000 (Bio-Rad, USA), and the gray values were measured using Image J software. Full and uncropped western blots are shown in the Supplemental Material raw data.

### Transcription in vitro and RNA pull-down assay

For in vitro transcription, plasmids containing two T7 promoters were digested using a single restriction endonuclease. The T7 High Yield RNA Transcription Kit (Vazyme, Nanjing, China) was used to transcribe forward and reverse linear DNA templates to RNA (sense and antisense probe), which was subsequently purified using the RNeasy Mini Kit (Qiagen, Germany). The Pierce™ RNA 3′ End Desthiobiotinylation Kit (Thermo Fisher Scientific, Rockford, USA) was used to label biotin to the 3′ end of RNA. For the RNA pull-down assay, 1 × 10^7^ cells were washed in ice-cold PBS, lysed in 500 μl co-IP buffer (Thermo Scientific) supplemented with a cocktail of proteinase inhibitors, phosphatase inhibitors, and RNase inhibitors (Invitrogen), and then incubated with 3 μg biotinylated DNA oligo probes against sense or antisense for 2 h at room temperature. A total of 50 μl washed streptavidin magnetic beads (Thermo Fisher Scientific, Rockford, USA) were added to each binding reaction and further incubated for another hour at room temperature. The beads were briefly washed five times with elution buffer. Finally, the retrieved proteins were subjected to mass spectrometry or western blot analysis.

### Silver staining and mass spectrometry analysis

Silver staining was performed using the Fast Silver Stain Kit (Beyotime, Shanghai, China), as described previously, while MS was performed by BGI Genomics (Shenzhen, China). Protein identification uses experimental MS/MS data and aligns them with theoretical MS/MS data from the database to obtain results. The entire process starts by converting raw MS data into a peak list and then searching for matches in the database. The search results were subjected to strict filtering and quality control, and possible protein identification was performed. Finally, from the final protein identification list, functional annotation analyses, such as GO, COG/KOG, and pathway analysis, were performed.

### RNA immunoprecipitation

RIP experiments were performed using an RNA Immunoprecipitation kit (Geneseed, Guangzhou, China), according to the manufacturer’s instructions. Co-precipitated RNA was detected using qRT-PCR.

### Lipidomics

Lipid extraction and mass spectrometry-based lipid detection of cell pellets were performed as described by the experimental operation of Huang et al [54]. A quality control sample was prepared by mixing equal parts of the samples. During the experiments, we processed the analytical sample using the same parameters to evaluate the stability of the analytical performance and reliability of the data. Ultra-high-performance liquid chromatography-mass spectrometry analysis was performed using a Q Exactive Plus high-resolution mass spectrometer (Thermo Scientific, USA) equipped with an Ultimate 3000 UHPLC system (Thermo Scientific, USA). Lipid identification (structural identification) and a peak table containing the retention time, m/z, and peak area (peak matching) were assessed using MS-DIAL software. The data combination of the positive and negative ion modes was defined as the relative lipid abundance for subsequent statistical analysis.

### Actinomycin D assays

RBE and HCCC-9810 cells were seeded in six-well plates (1 × 10^6^ cells per well). Twenty-four hours later, cells were exposed to 2 μg/ml Actinomycin D (Sigma) and collected at indicated time points. The RNA stability was analyzed using qRT-PCR and normalized to the values measured in the mock treatment group (the 0 h group).

### Co-Immunoprecipitation

To detect protein–protein interactions, cells were lysed in 500 μl co-IP buffer supplemented with a cocktail of proteinase inhibitors, phosphatase inhibitors, and RNase inhibitors. The lysates were added to the same IgG species used for immunoprecipitation as the normal IgG and agarose beads (Beyotime, Shanghai, China) and shaken slowly at 4 °C for 30 min to 2 h. The supernatant is centrifuged at 1000 g for 5 min and used for immunoprecipitation with agarose beads, which were pre-incubated with the corresponding antibodies. After incubation at 4 °C overnight, beads were washed 5 times with PBS. SDS sample buffer was added to the agarose beads, and immunoprecipitates were used for western blot analysis.

### Lipid peroxidation labeling

4,4-difluoro-5-(4-phenyl-1,3-butadienyl)-4-bora-3a,4a-diaza-s-indacene-3-undecanoic acid (BODIPY® 581/591 C11) (Invitrogen, Carlsbad, CA, USA), a fluorescent FA analog, was used. This dye shifts from red to green upon oxidation. Freshly cells were incubated with BODIPY 581/591 as a free radical sensor for 30 min at 37 °C. Then, cells were rinsed twice with PBS. The fluorescence inside the cells was analyzed using flow cytometry. Cells were selected by FSC/SSC gate. Subsequently, BODIPY C11 non-oxidized positive hepatocytes (red cells, PE filter) were gated, then positive green cells were selected (BODIPY oxidized positive cells) (FITC filter). The limits of the markers were established using unstained cells (negative control). Then, they were rinsed twice with PBS and measured on a FACSCanto II flow cytometer (FACS Calibur, BD Biosciences, USA). Data analysis was performed using the FlowJo 10.6.2 software.

### Statistical analysis

Results are shown as the mean ± SD. SPSS 22.0 (IBM Corp., Armonk, NY, USA), R 4.2.2 (http://www.r-project.org), and GraphPad Prism 8.0 were used for comparison analysis. ImageJ was used to analyze the colocalization between circMBOAT2 and PTBP1 by the function of coloc-2. Unpaired Student’s *t*-test was used for comparisons between the two groups. Fisher’s exact test was used to determine the association between the expression of circMBOAT2 with patients’ clinic pathological parameters. Correlation between the expression of circMBOAT2 and Ki67, PTBP1, and FASN was evaluated by Pearson correlation analysis. Partial least squares discriminant analysis (PLS-DA), including loading plot and variable importance in projection value, was performed by MetaboAnalyst 5.0 using the online server. Statistical significance was set at *P* < 0.05.

## Supplementary information

Supplementary Materials

Supplementary Figures

Supplementary Table 1. Untargeted lipidomics identified 474 lipids belonging to 14 classes.

Supplementary Table 2. The proteins pulled down by the circMBOAT2 probe.

Reproducibility checklist

raw data

## Data Availability

The datasets generated and/or analyzed during the current study are available from the corresponding author upon reasonable request.
