# Supplementary material for: CircRNA MBOAT2 promotes intrahepatic cholangiocarcinoma progression and lipid metabolism reprogramming by stabilizing PTBP1 to facilitate FASN mRNA cytoplasmic export
Source: Cell Death Dis. 2023 Jan 12;14(1):20. doi: 10.1038/s41419-022-05540-y (PMC9837196; doi:10.1038/s41419-022-05540-y)
Supplement: Supplementary file 1 — Supplementary Materials [file 41419_2022_5540_MOESM1_ESM.doc]

**Supplementary Materials**

The sequences of all primers and oligonucleotide used in the study.

| Gene | Forward primer (5’ to 3’) | Reverse primer (5’ to 3’) |
| --- | --- | --- |
| β-actin  GAPDH | CATGTACGTTGCTATCCAGGC  CGGAGTCAACGGATTTGGTCGTAT | CTCCTTAATGTCACGCACGAT  AGCCTTCTCCATGGTGGTGAAGAC |
| U6 | CTCGCTTCGGCAGCACA | AACGCTTCACGAATTTGCGT |
| circMBOAT2 (divergent primer)  circMBOAT2  (convergent primer) | GTCAACTTTGTAGTGTGCCAACT  GAGTGGAGAACATGCACAAGTCA | TTGTGCATGTTCTCCACTCC  GTAGATAAGTTCGAAACCAAATGGC |
| MBOAT2  PTBP1 | CTCGCTGGGACTTAATTTCCAA  TCTACTTGTGTCACTAACGGAC | GGTTCGTTCATAACACACCCTT  TGAACTTCTTGCTGTCATTTCC |
| FASN  FASN splice | GTGGTGGGCTTGGTGAACTGTC  TCCAGCCTCGCTCTCC | AGGTGCTGCTGAGGTTGGAGAG  GCCGATGAGGTTGTCCCAGAACTC |
| Oligonucleotide targets | Sense (5’ to 3’) | Antisense (5’ to 3’) |
| si-circMBOAT2#1 | GAGAACAUGCACAAGUCAA | CUCUUGUACGUGUUCAGUU |
| si-circMBOAT2#2 | CAUGCACAAGUCAACUUUG | GUACGUGUUCAGUUGAAAC |
| si-NC | UUCUCCGAACGUGUCACGU | ACGUGACACGUUCGGAGAA |
| si-PTBP1#1 | GCCUCAACGUCAAGUACAA | UUGUACUUGACGUUGAGGC |
| si-PTBP1#2 | GCGUCGUCAAAGGAUUCAA | UUGAAUCCUUUGACGACGC |
| si-FASN#1  si-FASN#2  si-FASN#3 | GCCGAGUACAAUGUCAACA  CUGCUAGGUAUGGAGUUCU  CAUGGAGCGUAUCUGUGAG | UAGCUCCUCAAACAGCUGC  AGAACUCCAUACCUAGCAG  CUCACAGAUACGCUCCAUG |
