# Supplementary material for: CircRNA MBOAT2 promotes intrahepatic cholangiocarcinoma progression and lipid metabolism reprogramming by stabilizing PTBP1 to facilitate FASN mRNA cytoplasmic export
Source: Cell Death Dis. 2023 Jan 12;14(1):20. doi: 10.1038/s41419-022-05540-y (PMC9837196; doi:10.1038/s41419-022-05540-y)
Supplement: Supplementary file 2 — Supplementary Figures [file 41419_2022_5540_MOESM2_ESM.doc]

**Supplementary Figures**

**
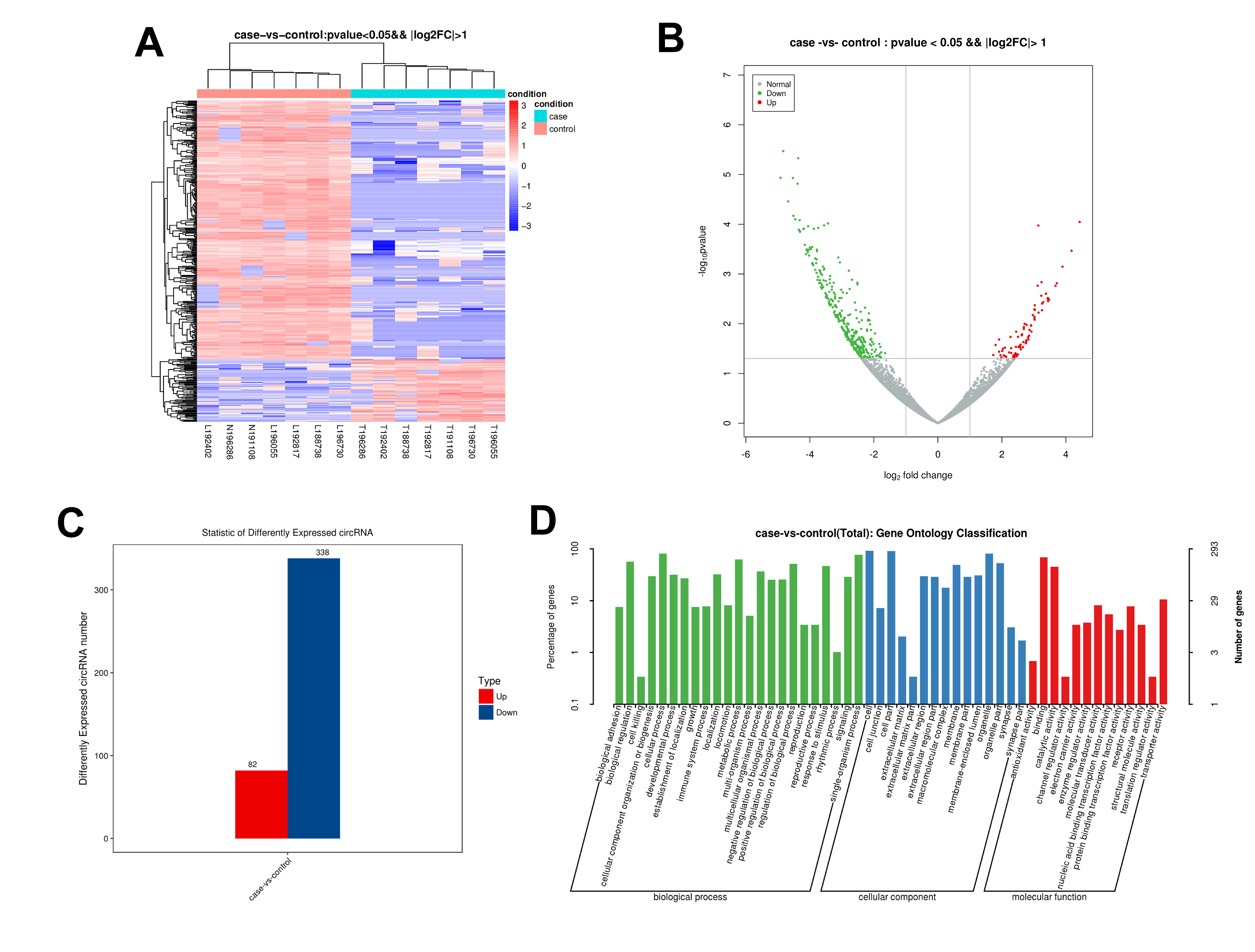
**

**Supplementary Figure 1. RNA-seq of ICC and adjacent non-tumor tissues.**

**a-c** The heat map and volcano plot showed that there were 82 up-regulated and 338 down-regulated circRNAs in ICC tissue compared to adjacent non-tumor tissue by RNA sequencing analysis. **d** GO terms in ICC tissue compared to adjacent non-tumor tissue by RNA sequencing.

**
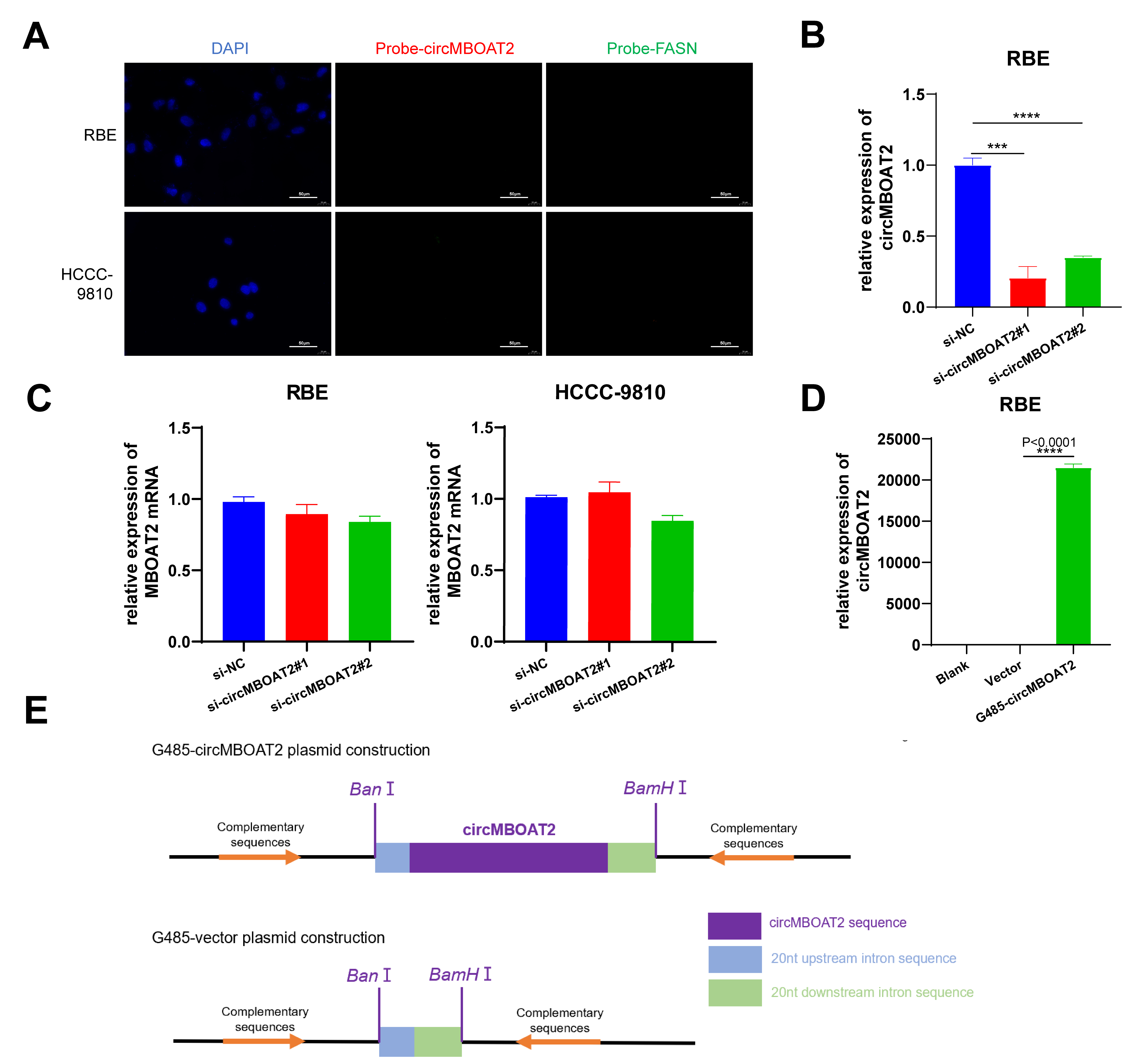
**

**Supplementary Figure 2. Construction of circMBOAT2 knockdown and overexpression model in ICC cells.**

**a** Negative probe as control in the RNA FISH. **b, d** The qRT-PCR method was applied to detect the expression levels of circMBOAT2 after transfected with si-NC, si-circMBOAT2#1, si-circMBOAT2#2 or with G485-vector, G485-circMBOAT2 in RBE cells. The expression of circMBOAT2 was normalized to β-actin. **c** The qRT-PCR method was applied to detect the expression levels of circMBOAT2 after transfected with si-NC, si-circMBOAT2#1, si-circMBOAT2#2 in RBE and HCCC-9810 cells. **e** A sketch map for plasmid construction of circMBOAT2 overexpression. Significant differences between groups were analyzed by t-test. Error bars represent the means ± SEM of three independent experiments.


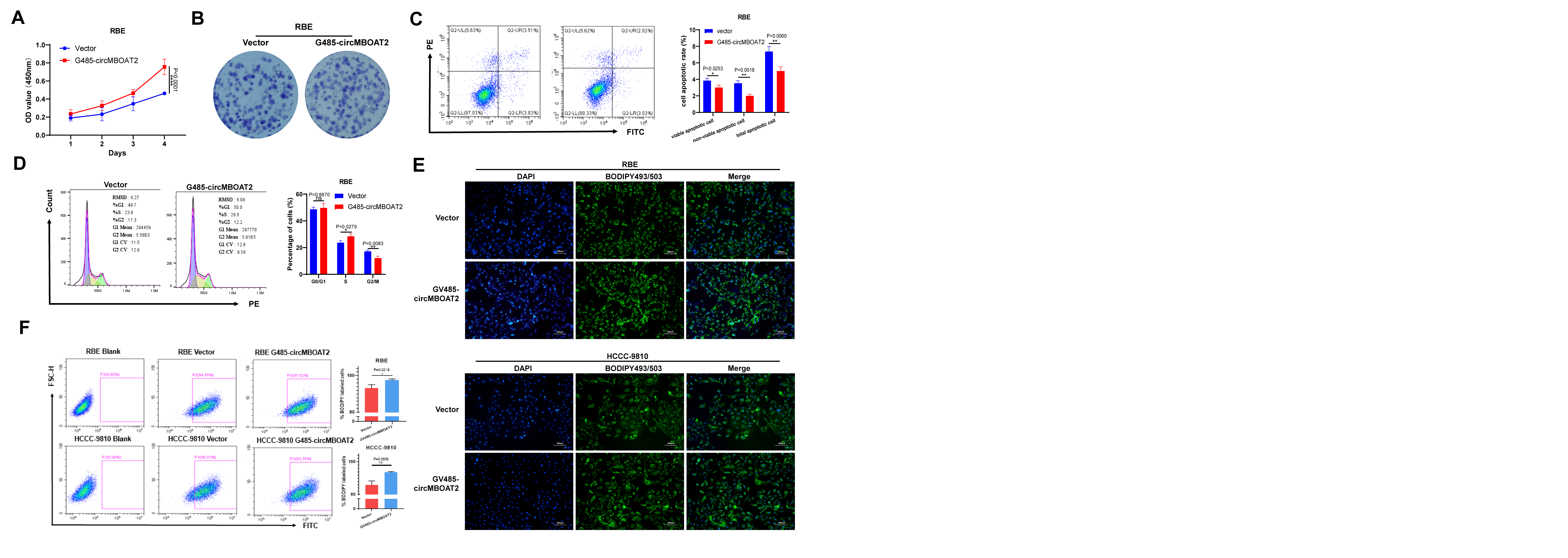


**Supplementary Figure 3. CircMBOAT2 promotes ICC progression in vitro.**

**a** CCK8 assay for cell proliferation capacity. The results showed that overexpression of circMBOAT2 promoted the viability of RBE cells. **b** Clone formation assay. The results showed that overexpressing circMBOAT2 promoted the proliferative capacity of RBE cells. **c** Data are expressed as the early and late stages of apoptosis rate after G485-vector and G485-circMBOAT2 transfection of RBE cells. **d** The percentage cell phase distribution including G0/G1, S and G2/M phases after transfection of cells G485-vector and G485-circMBOAT2. **e** The neutral lipid droplets were detected by staining with BODIPY 493/503 in RBE and HCCC-9810 cells transfected by vector and GV485-circMBOAT2. Scale bar = 50 μm. **f** Flow cytometry to detect the neutral lipid droplets stained with BODIPY 493/503 in RBE and HCCC-9810 cells. FITC-A was used to detect BODIPY 493/503.

Significant differences between groups were analyzed by t-test. Error bars represent the means ± SEM of three independent experiments.


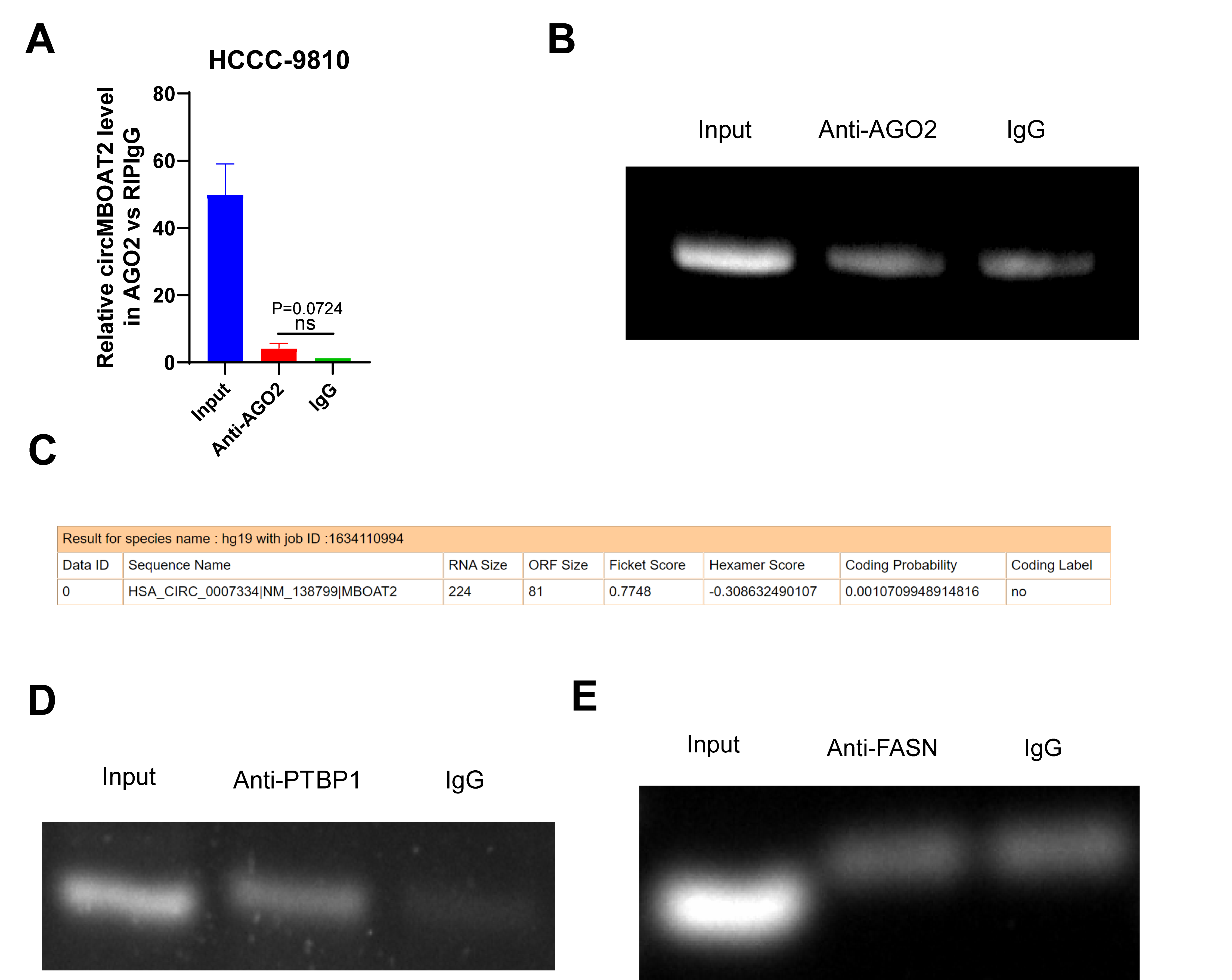


**Supplementary Figure 4. CircMBOAT2 performed functions by interacting with PTBP1 in ICC cells.**

**a, b** RIP assay. CircMBOAT2 was precipitated by an anti-AGO2 antibody, and detected by qRT-PCR or RT-PCR in HCCC-9810 cells. IgG was used as a negative control. **c** Prediction of translation potential of circMBOAT2 by an online database (<http://lilab.research.bcm.edu/>). **d, e** CircMBOAT2 was precipitated by an anti-PTBP1 antibody specifically but an anti-FASN antibody nonspecifically then detected by RT-PCR in HCCC-9810 cells. IgG was used as a negative control. Significant differences between groups were analyzed by t-test. Error bars represent the means ± SEM of three independent experiments.


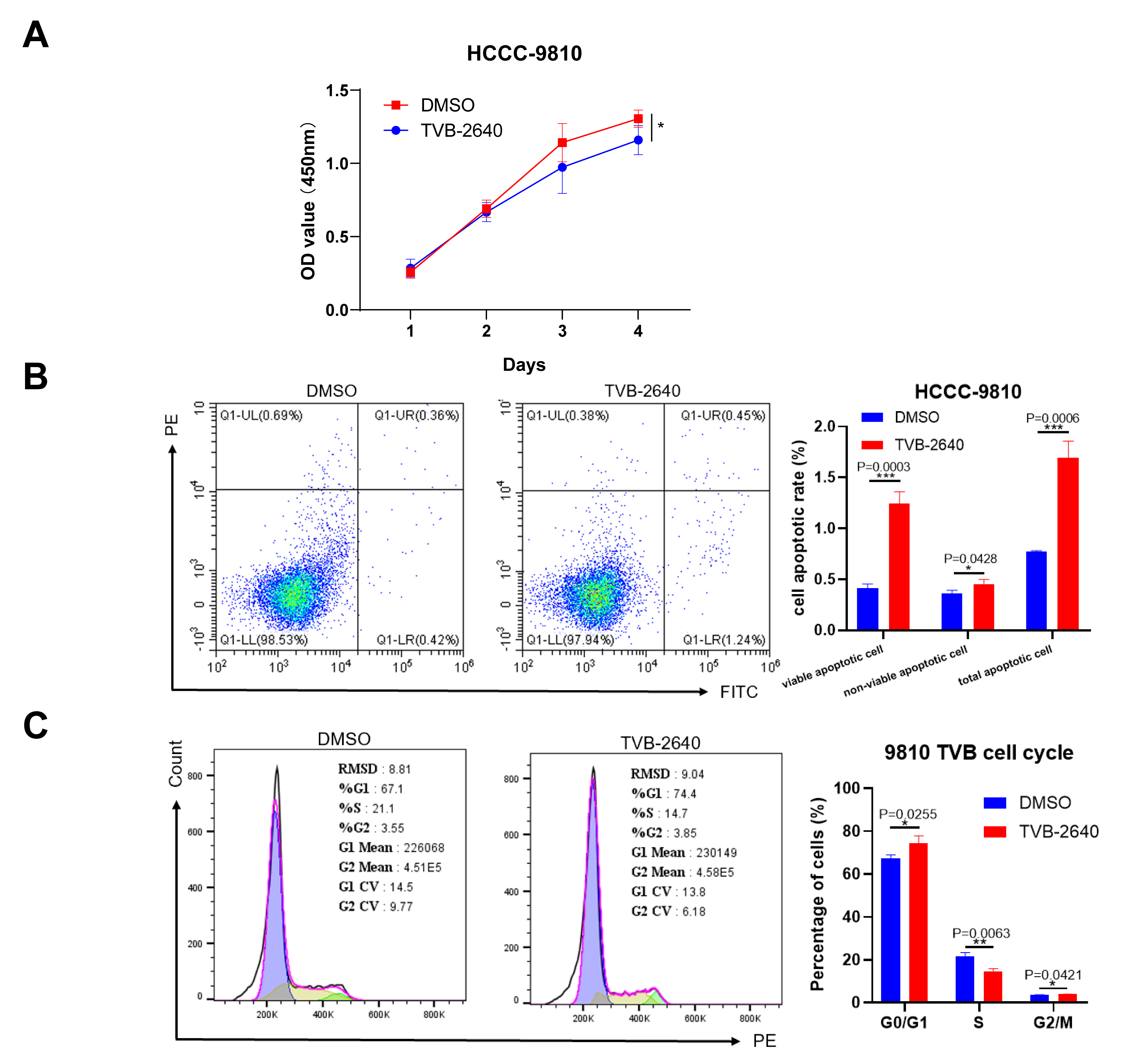


**Supplementary Figure 5. FASN promotes ICC progression in vitro.**

**a** CCK8 assay for cell proliferation capacity after treated by DMSO and TVB-2640 (0.1 μM) in HCCC-9810 cells. **b** Flow Cytometry was applied to determine the early and late stages of apoptosis rate after DMSO and TVB-2640 (0.1 μM) treatment in HCCC-9810 cells. **c** Flow Cytometry was applied to determine the percentage cell phase distribution including G0/G1, S and G2/M phases after treatment of HCCC-9810 cells with DMSO and TVB-2640 (0.1 μM). Significant differences between groups were analyzed by t-test. Error bars represent the means ± SEM of three independent experiments.


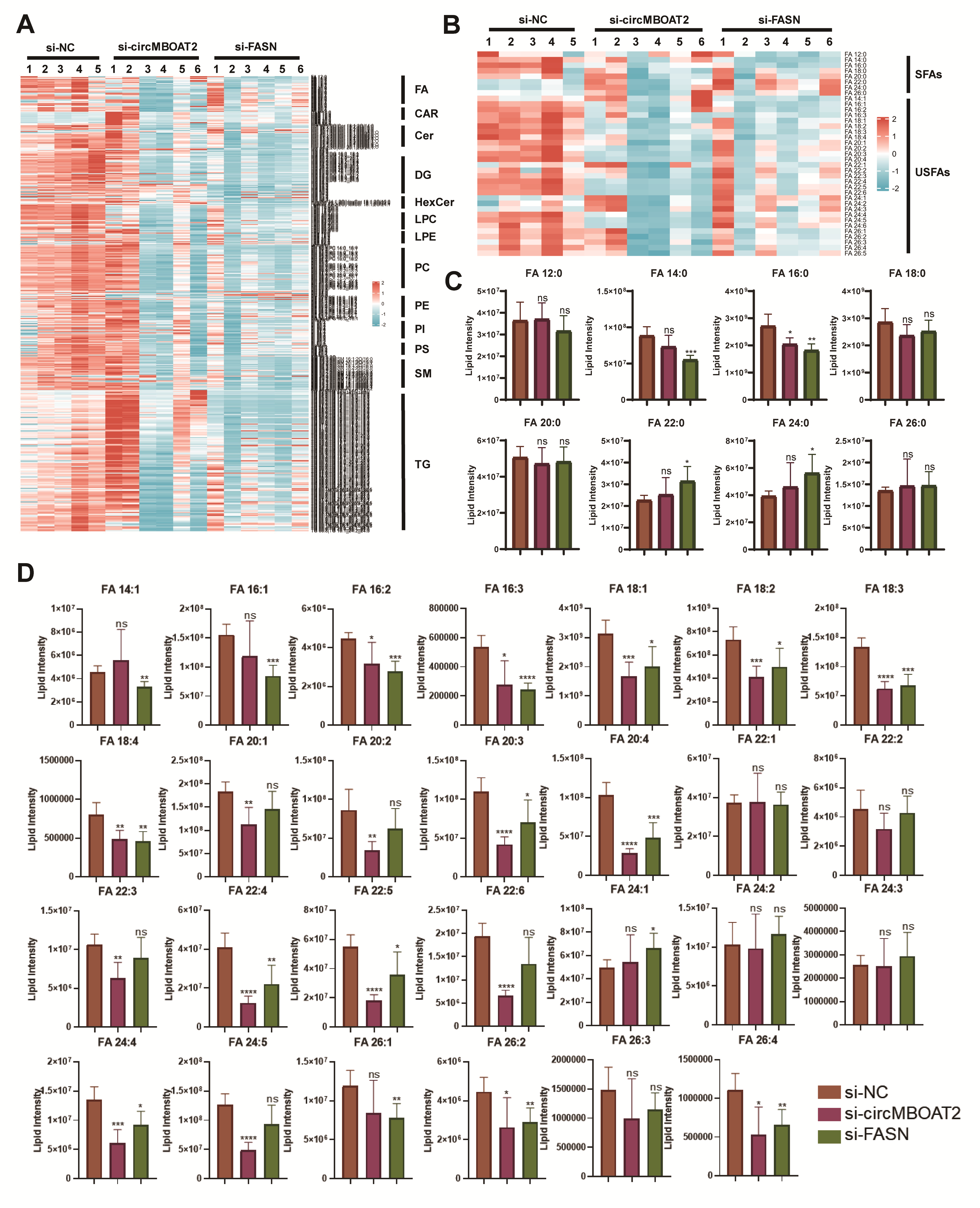


**Supplementary Figure 6. CircMBOAT2 and FASN regulate the lipid synthesis in ICC cells.**

**a** Lipid species identified in lipidomics analysis, HCCC-9810 cells transfected with si-NC, si-circMBOAT2 and si-FASN. **b** Fatty acids identified in lipidomics analysis, HCCC-9810 cells transfected with si-NC, si-circMBOAT2 and si-FASN. **c, d** All identified fatty acids were quantified by untargeted lipidomics analysis on HCCC-9810 cells transfected si-NC, si-circMBOAT2 and si-FASN. Data is shown as means ± SEM. Significant differences between groups were analyzed by by two-tailed Student’s t-test. Significant differences between groups were analyzed by t-test. Error bars represent the means ± SEM of three independent experiments.
