# Supplementary figures and images for: CircRNA MBOAT2 promotes intrahepatic cholangiocarcinoma progression and lipid metabolism reprogramming by stabilizing PTBP1 to facilitate FASN mRNA cytoplasmic export
Source: Cell Death Dis. 2023 Jan 12;14(1):20. doi: 10.1038/s41419-022-05540-y (PMC9837196; doi:10.1038/s41419-022-05540-y)

**Fig. 2F**

**
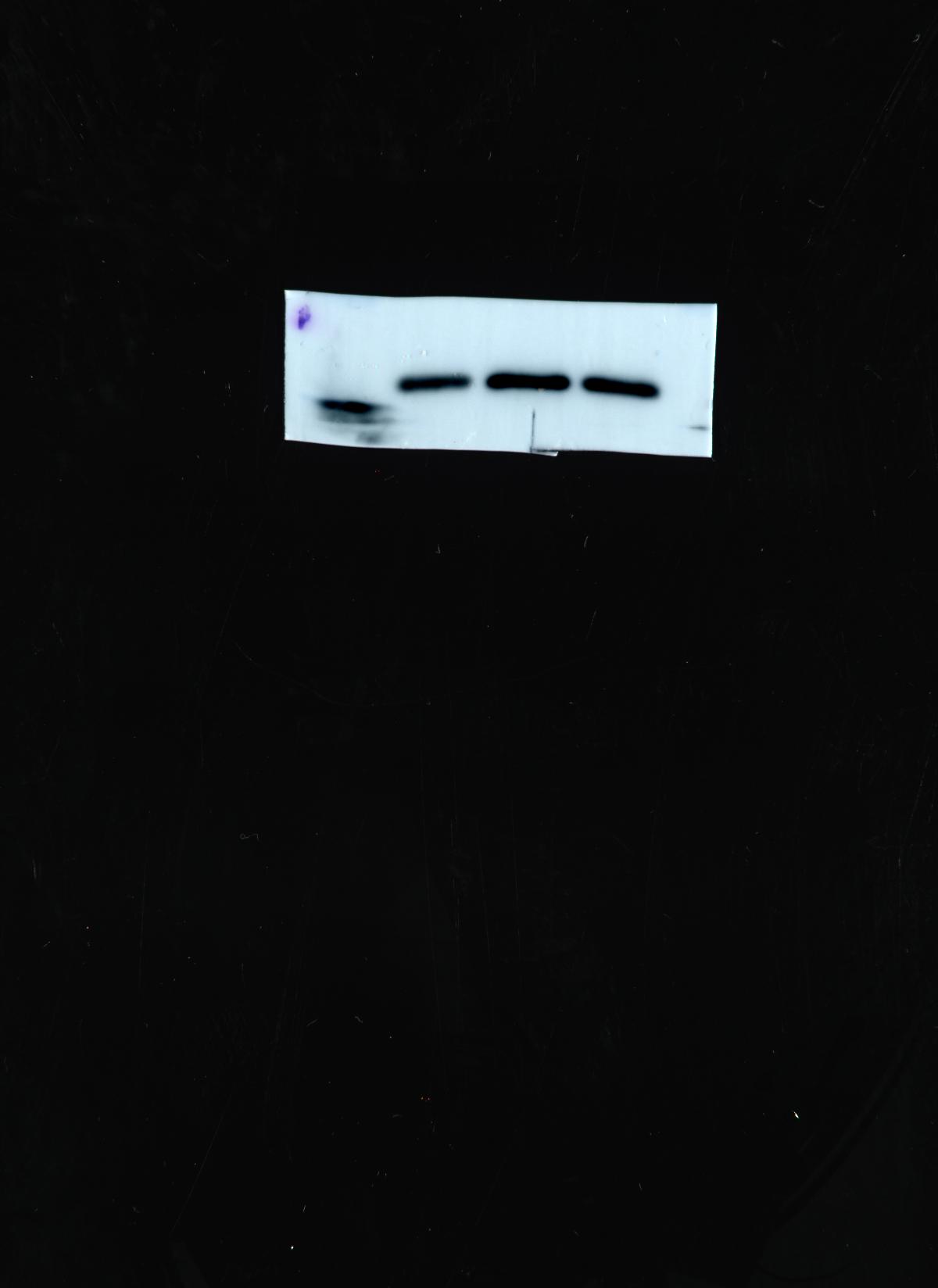
**

**
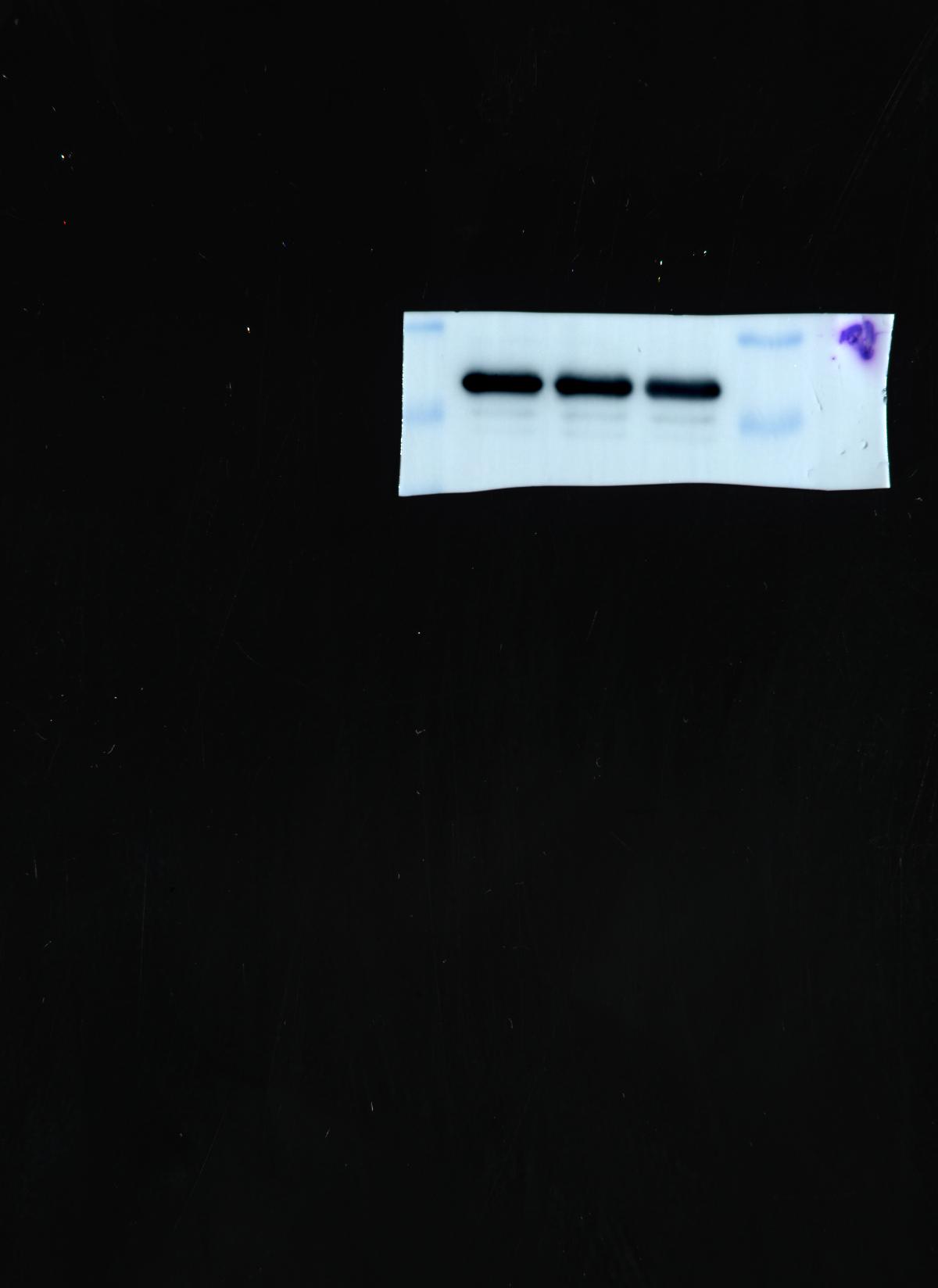
**

**
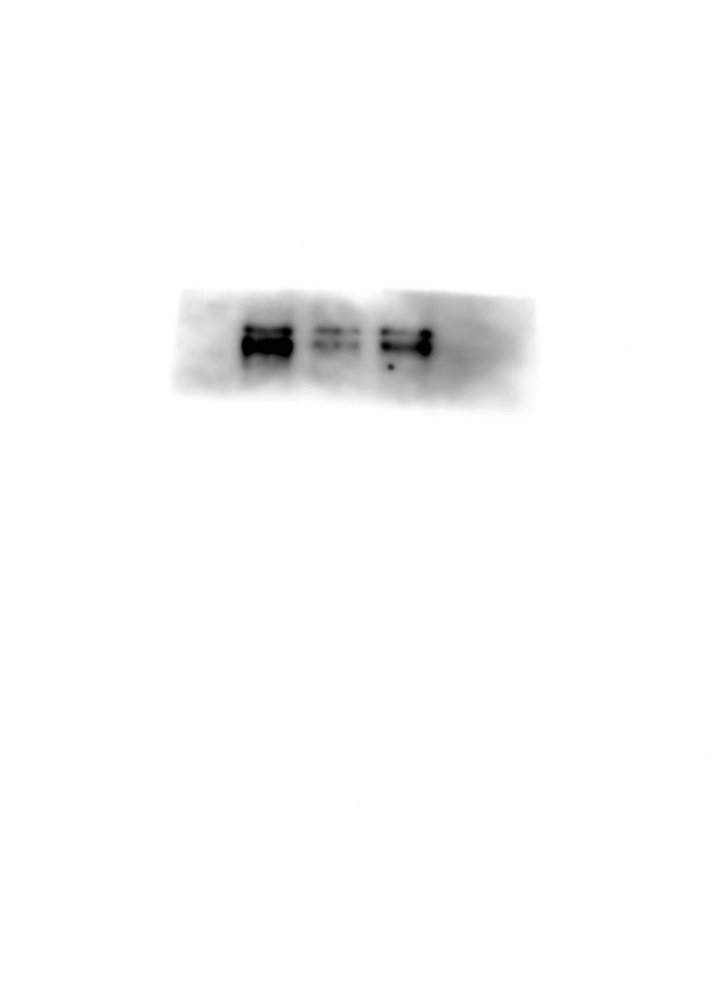
**

**
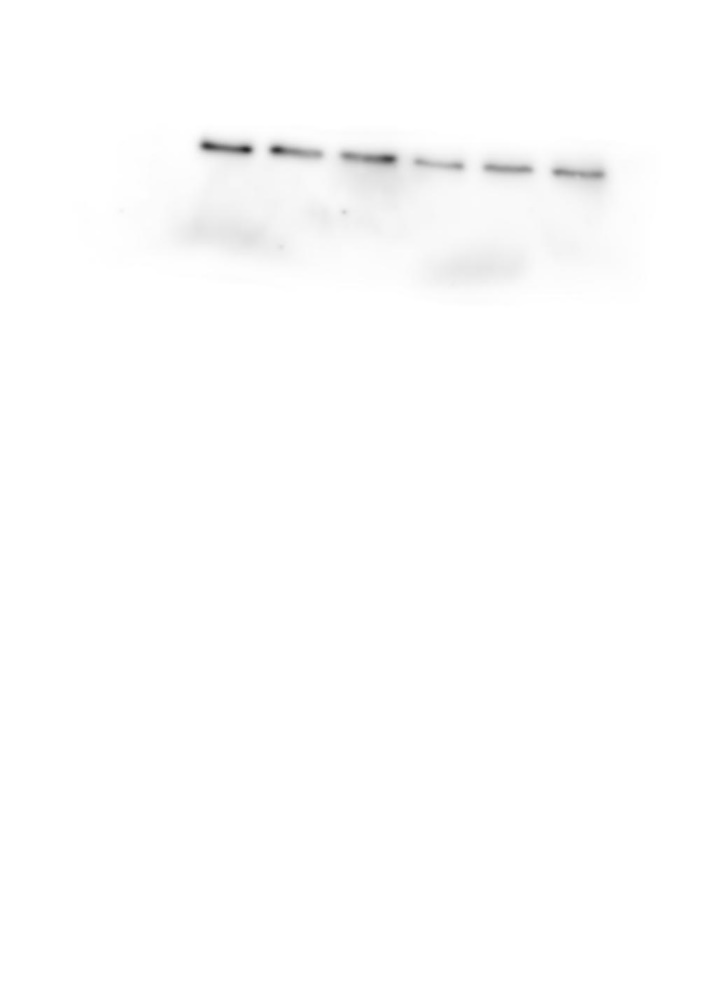
**

**
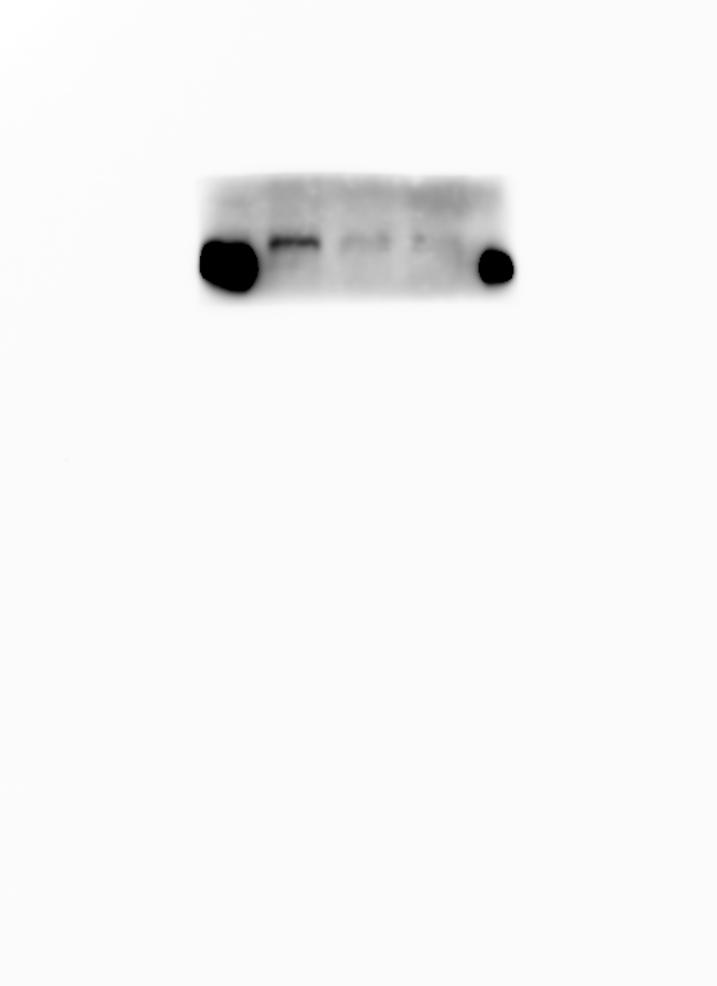
**

**
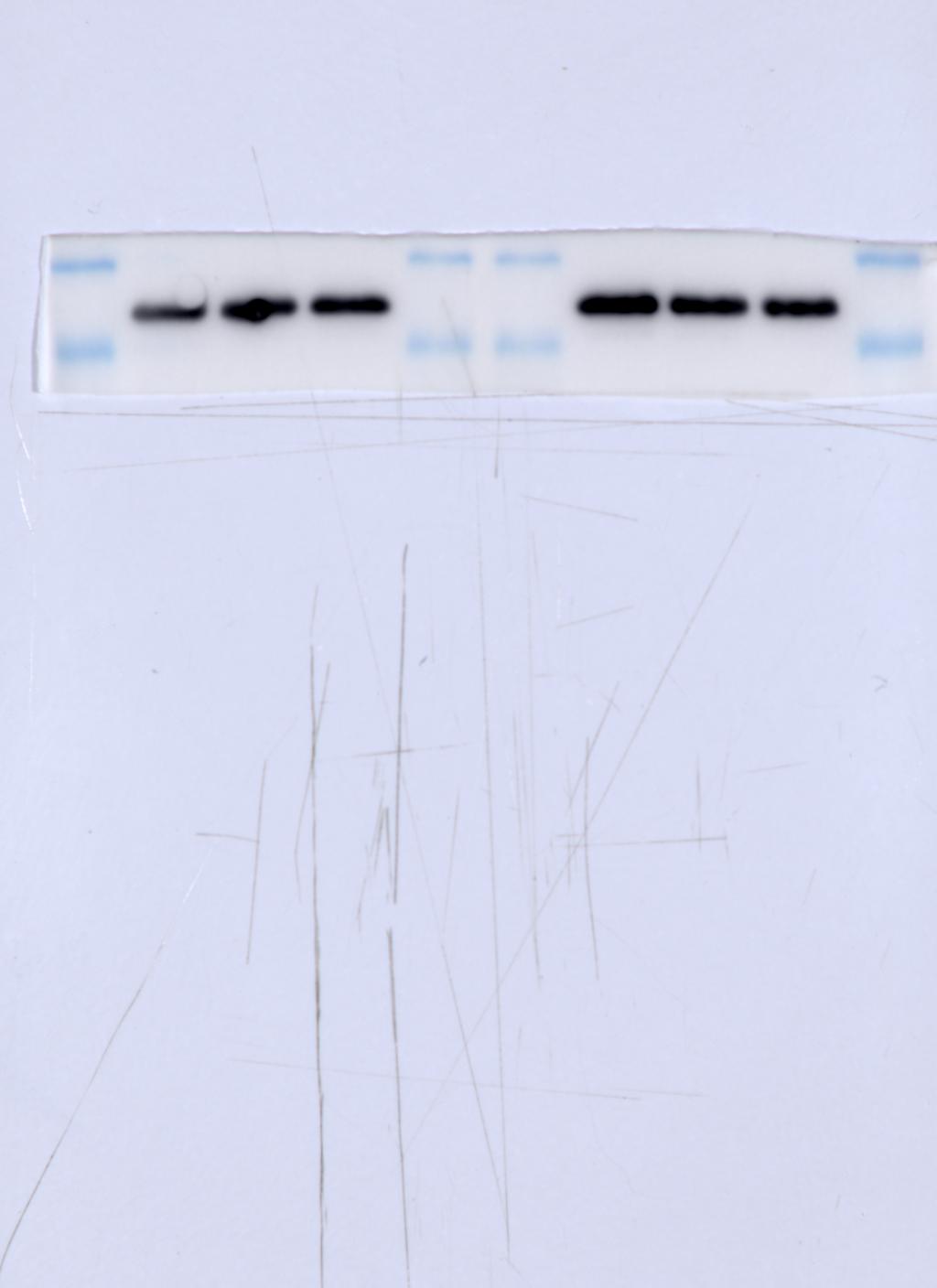
**

**
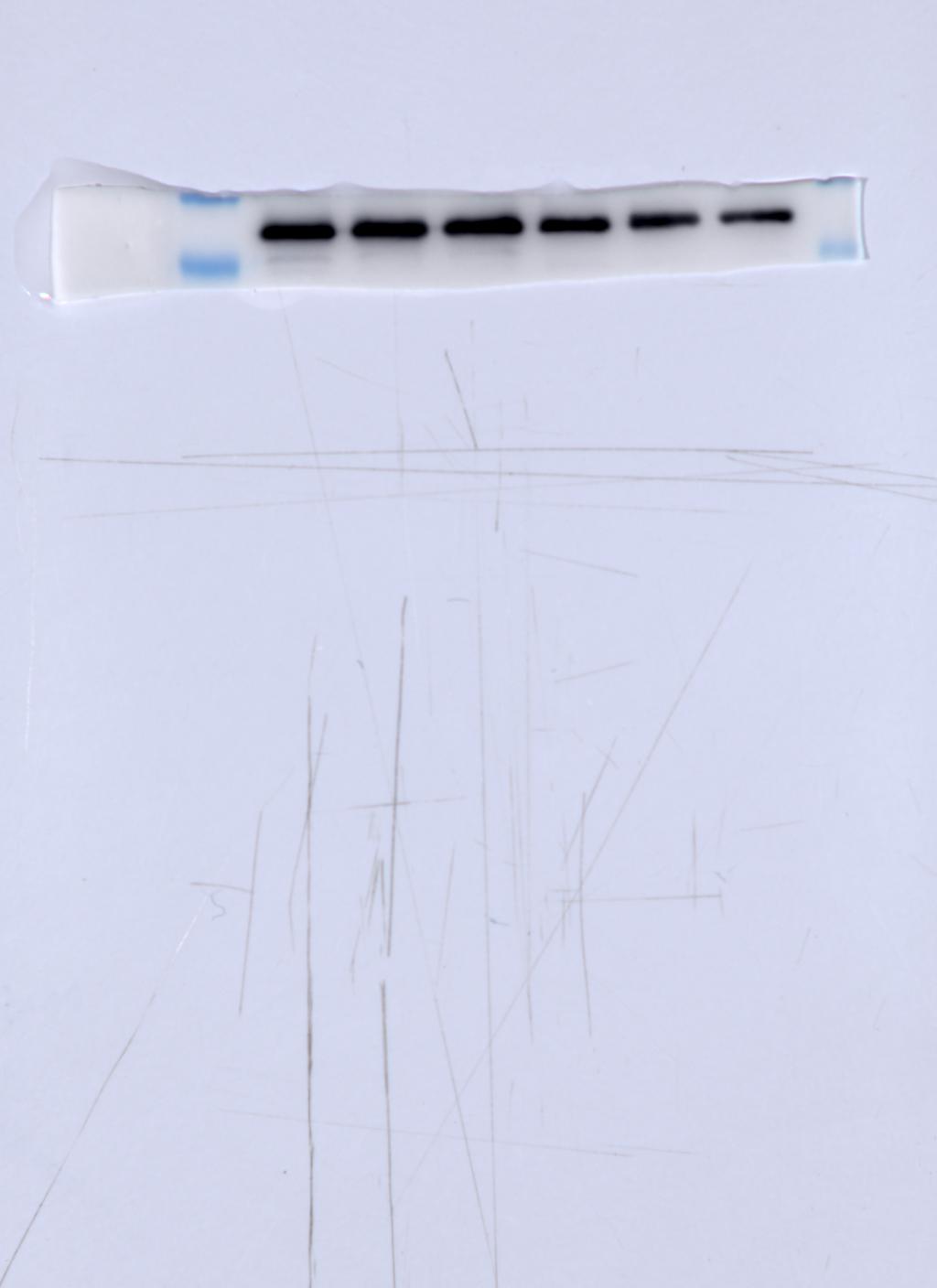
**

**Fig. 4C**


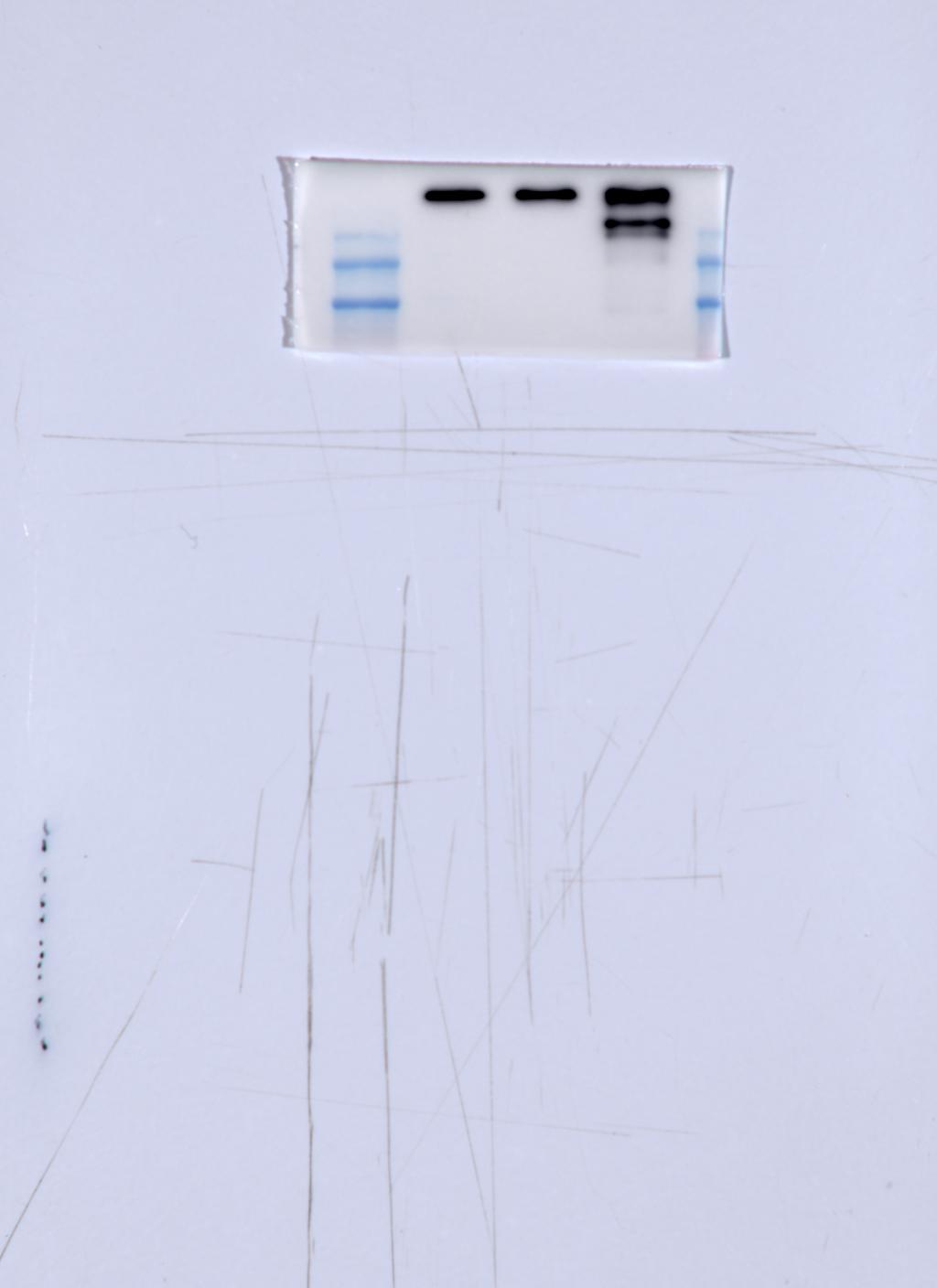

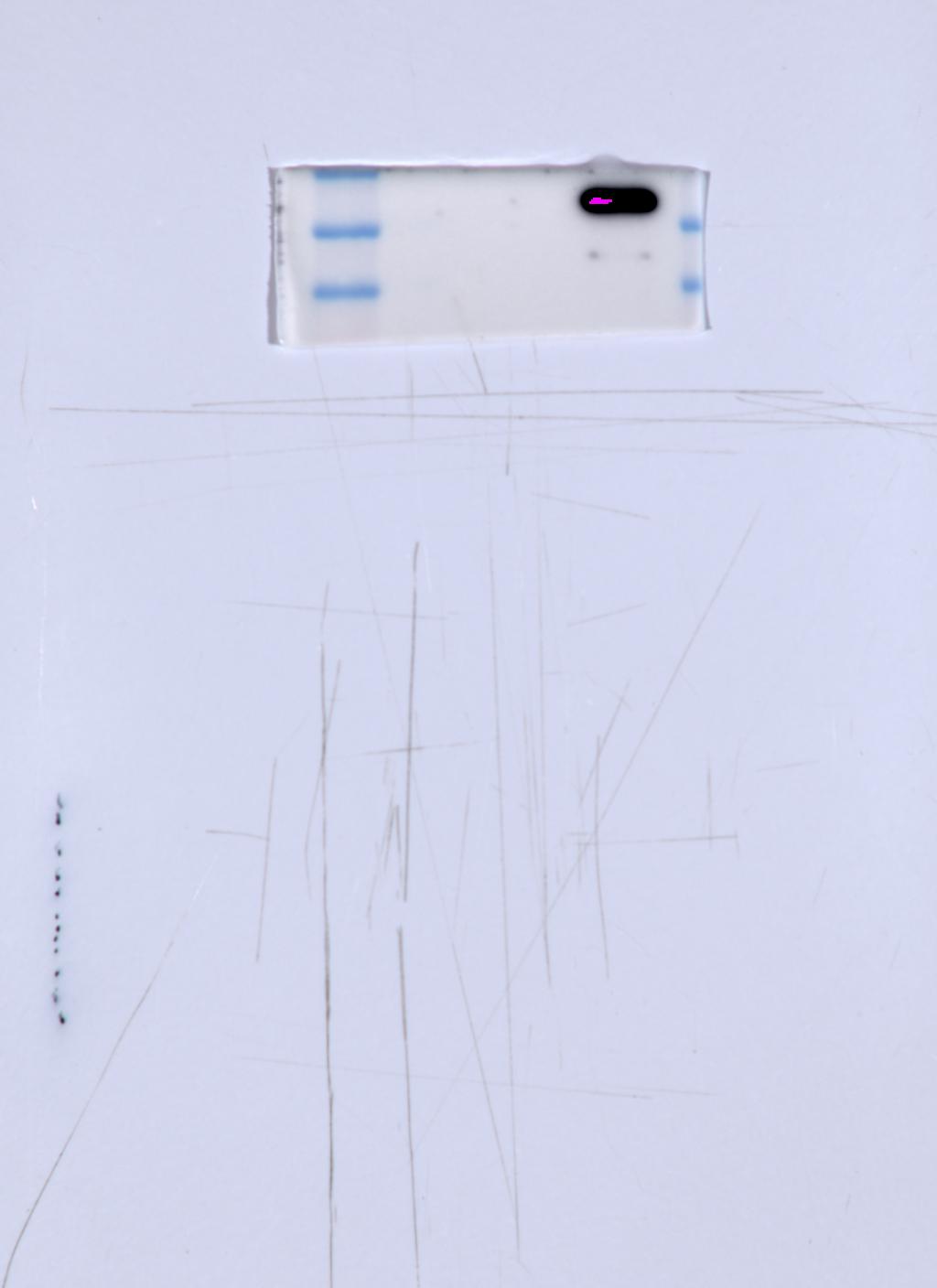

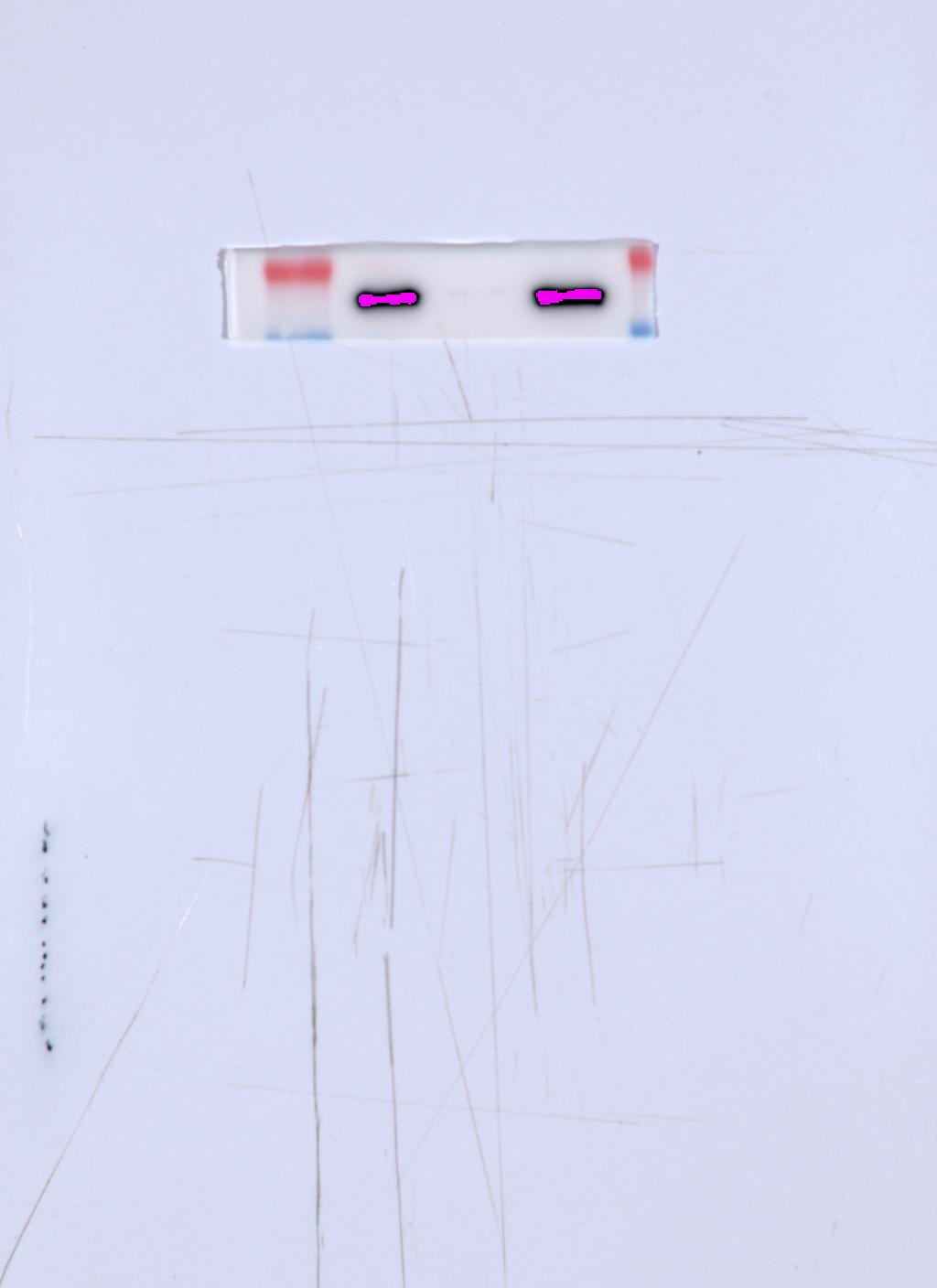


**Fig. 4G**


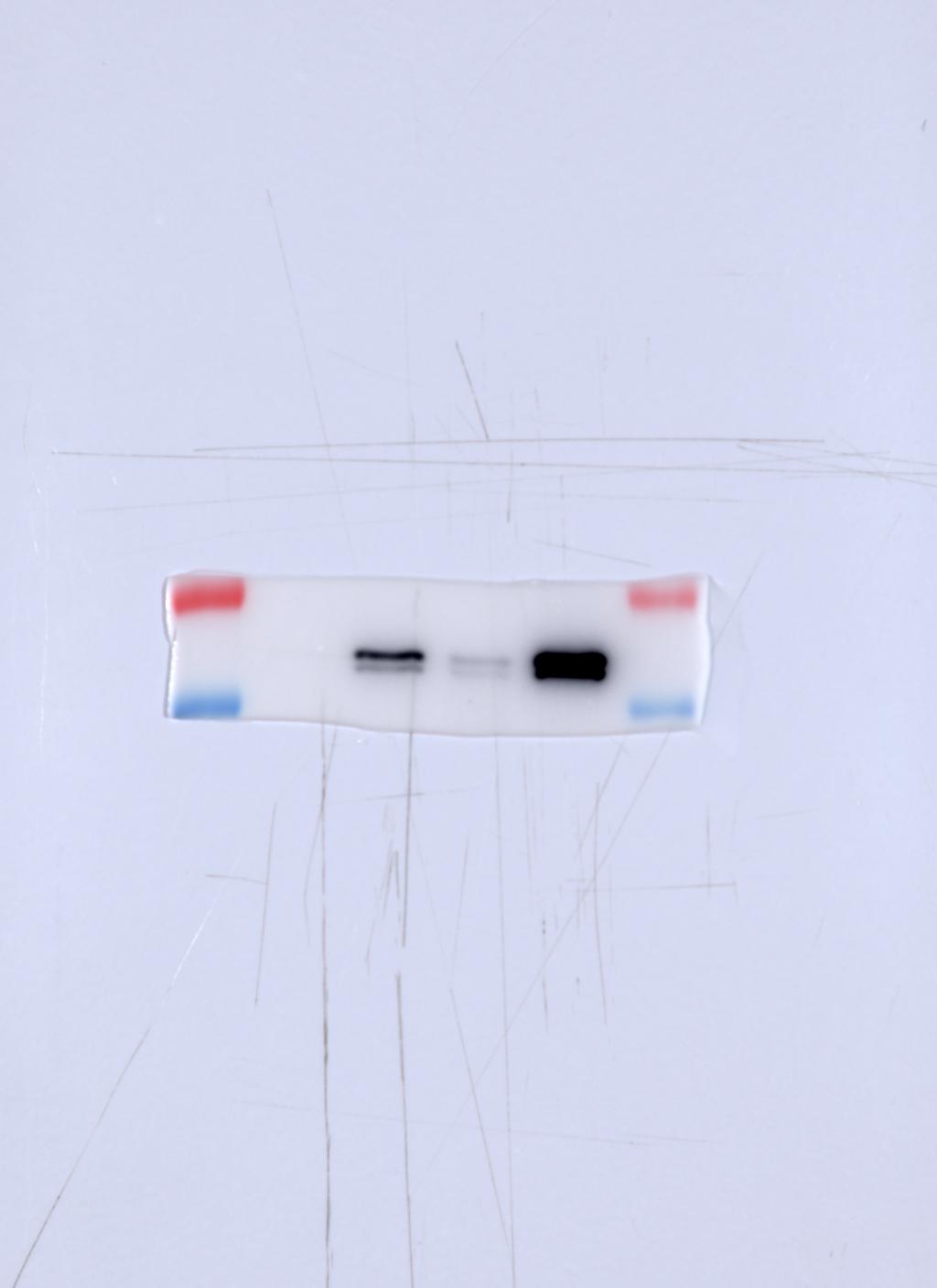

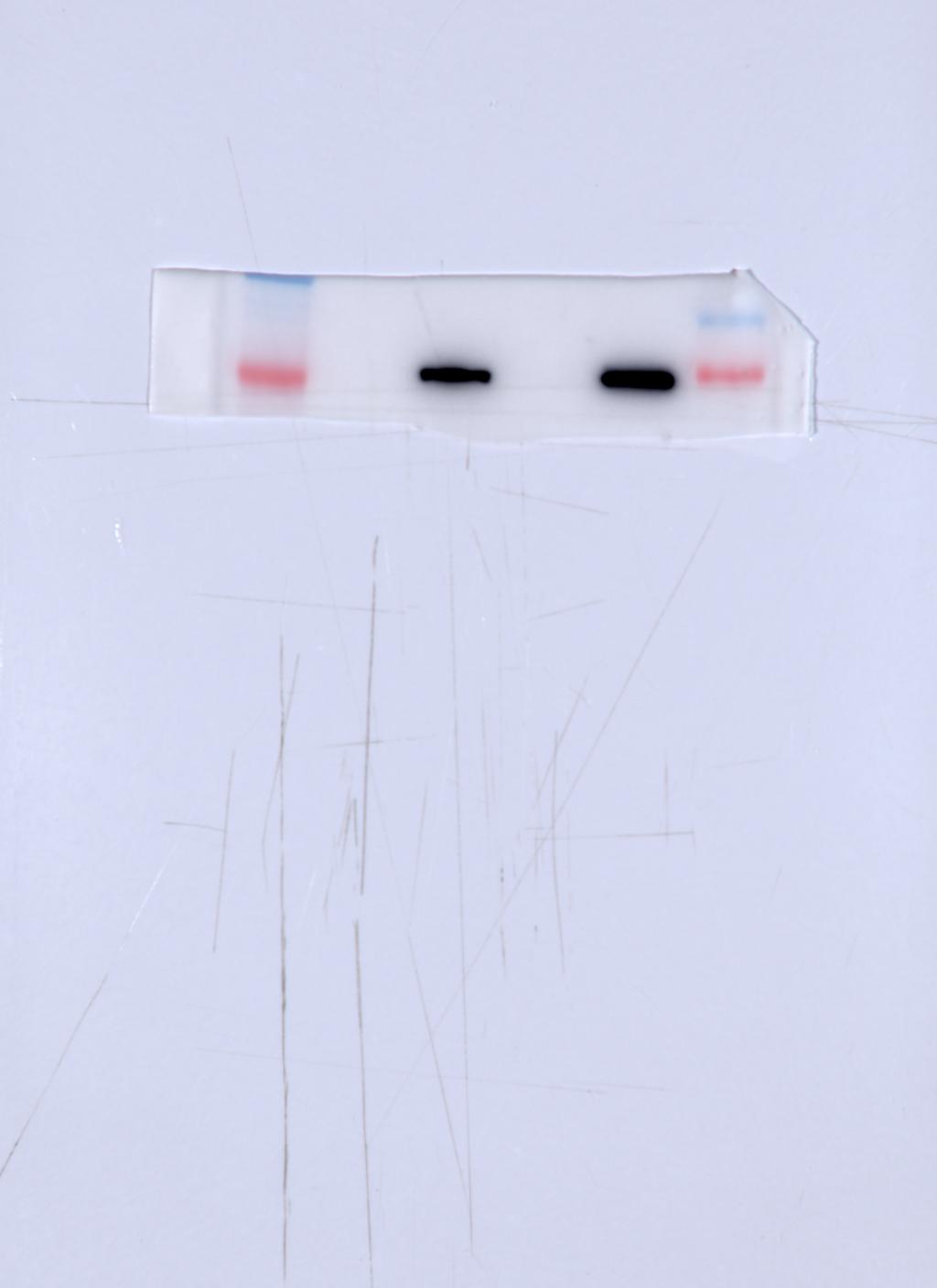

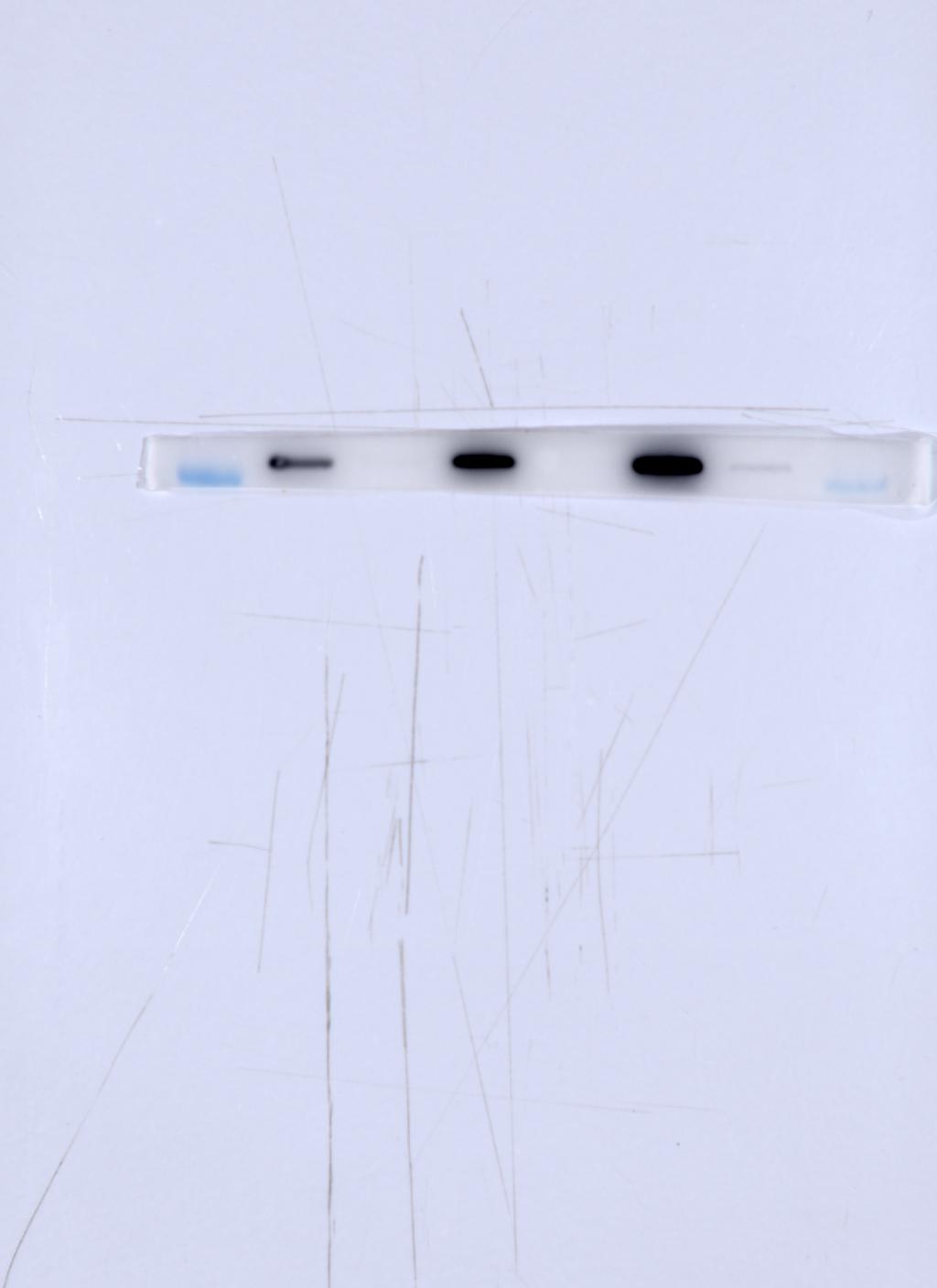


**Fig. 5A**


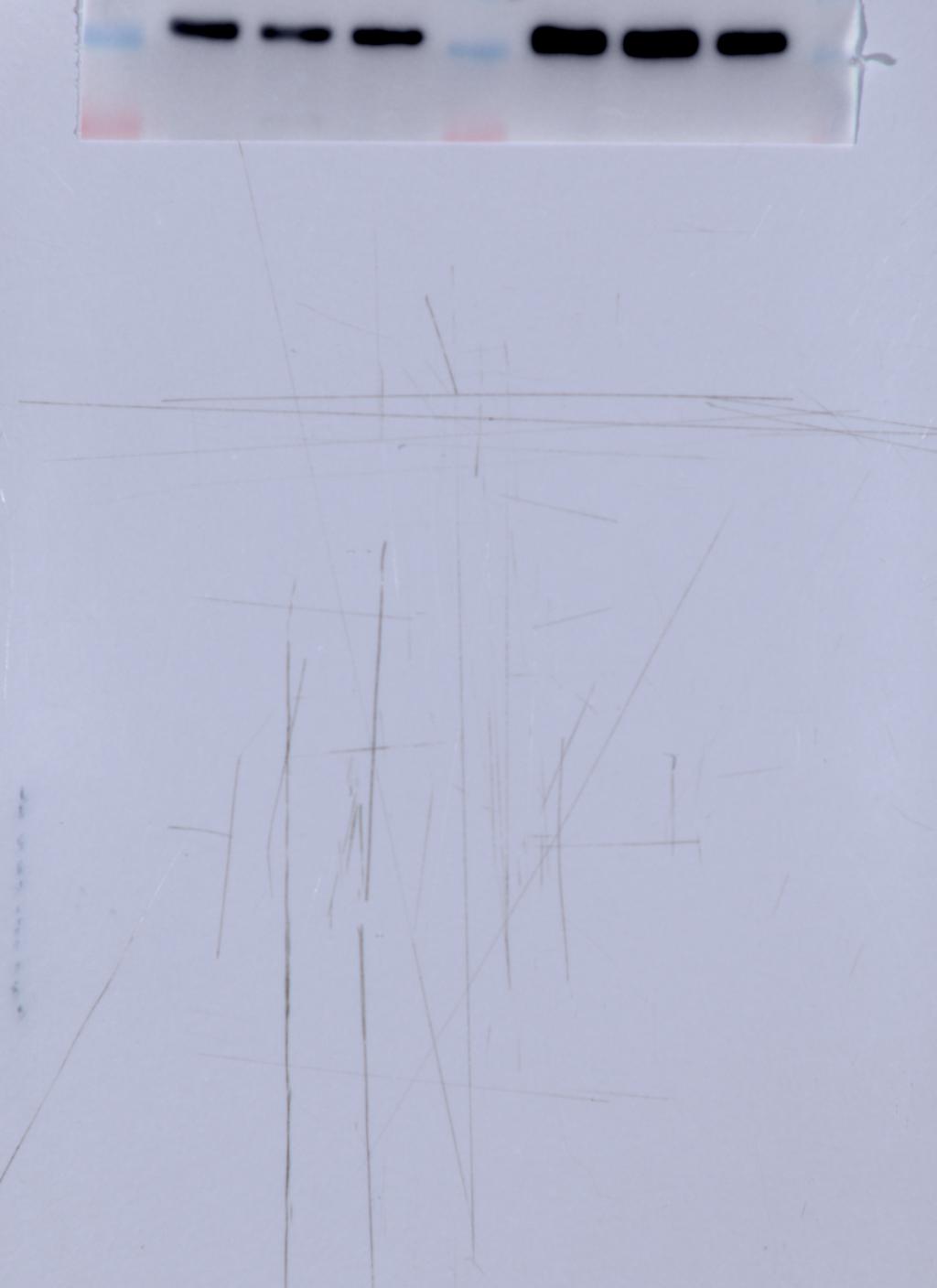

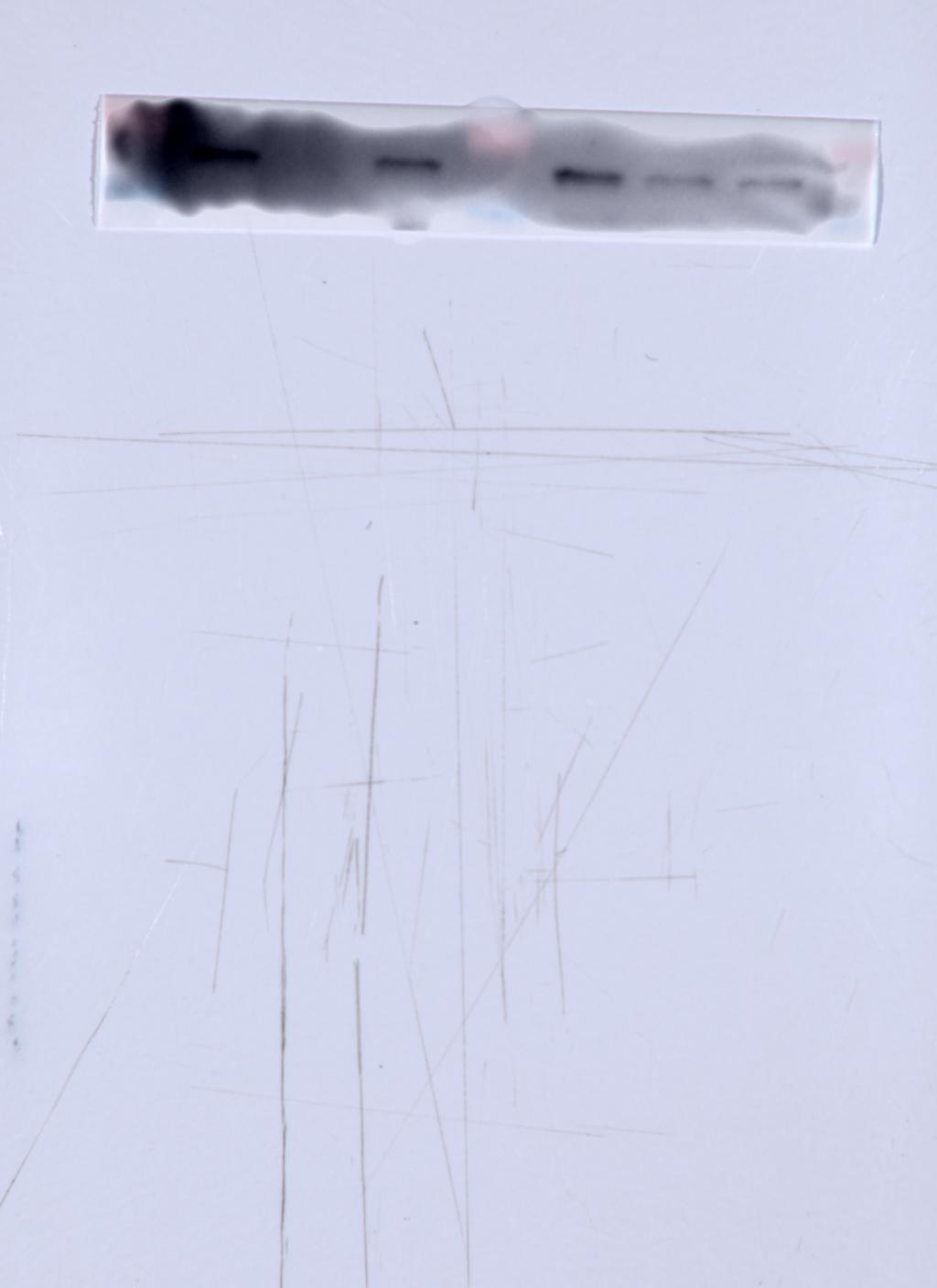


**Fig. 5B**


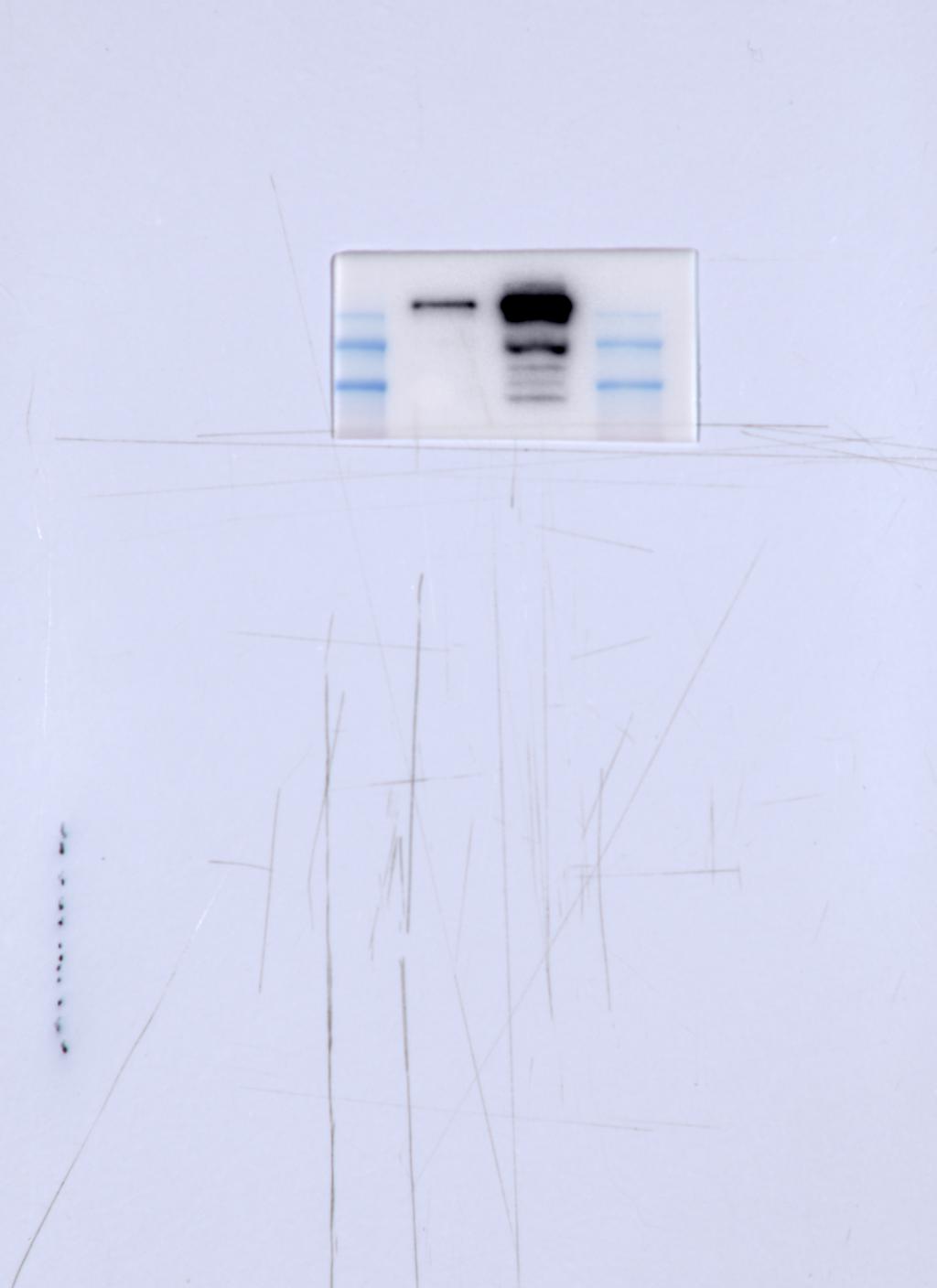

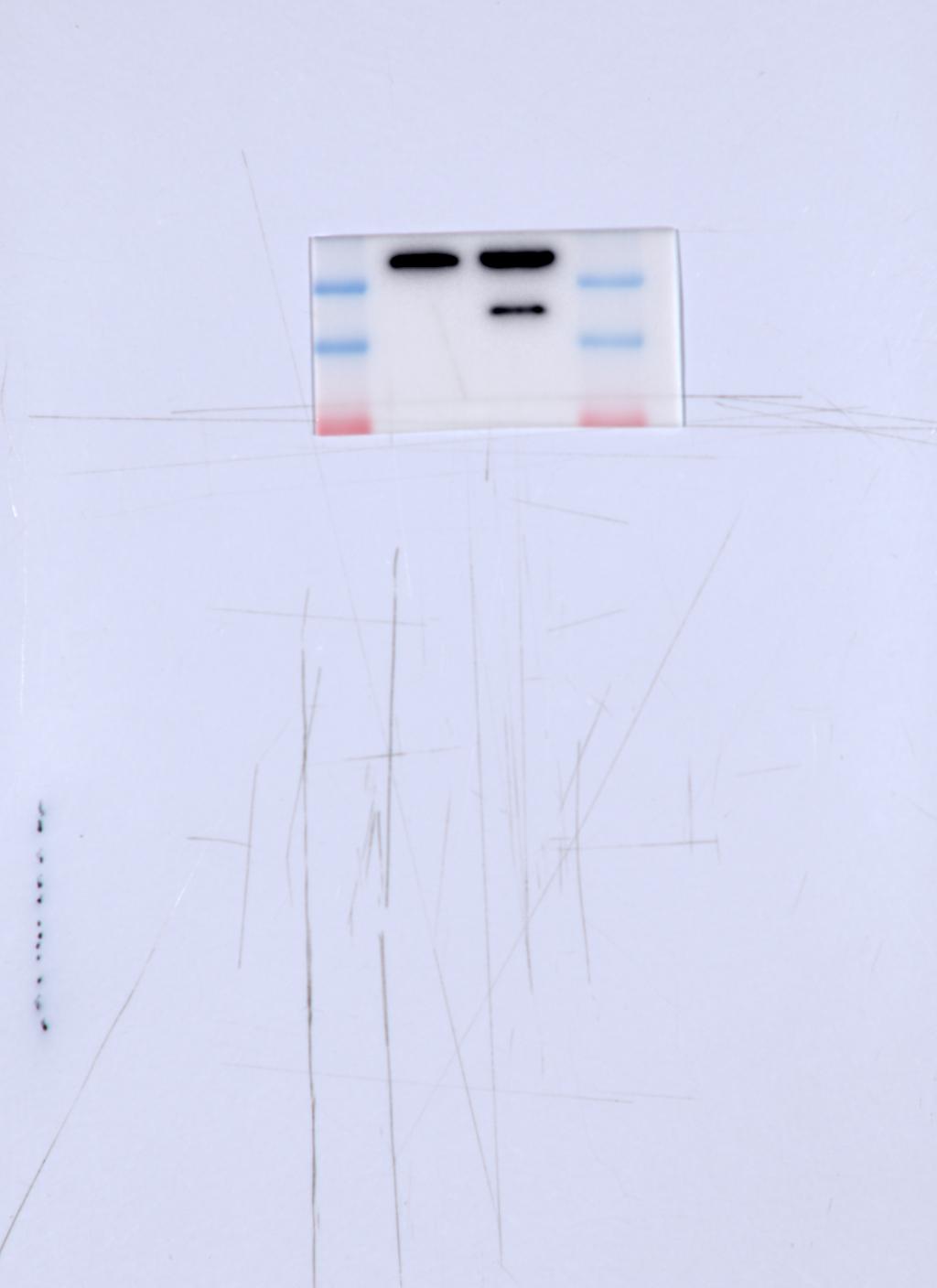

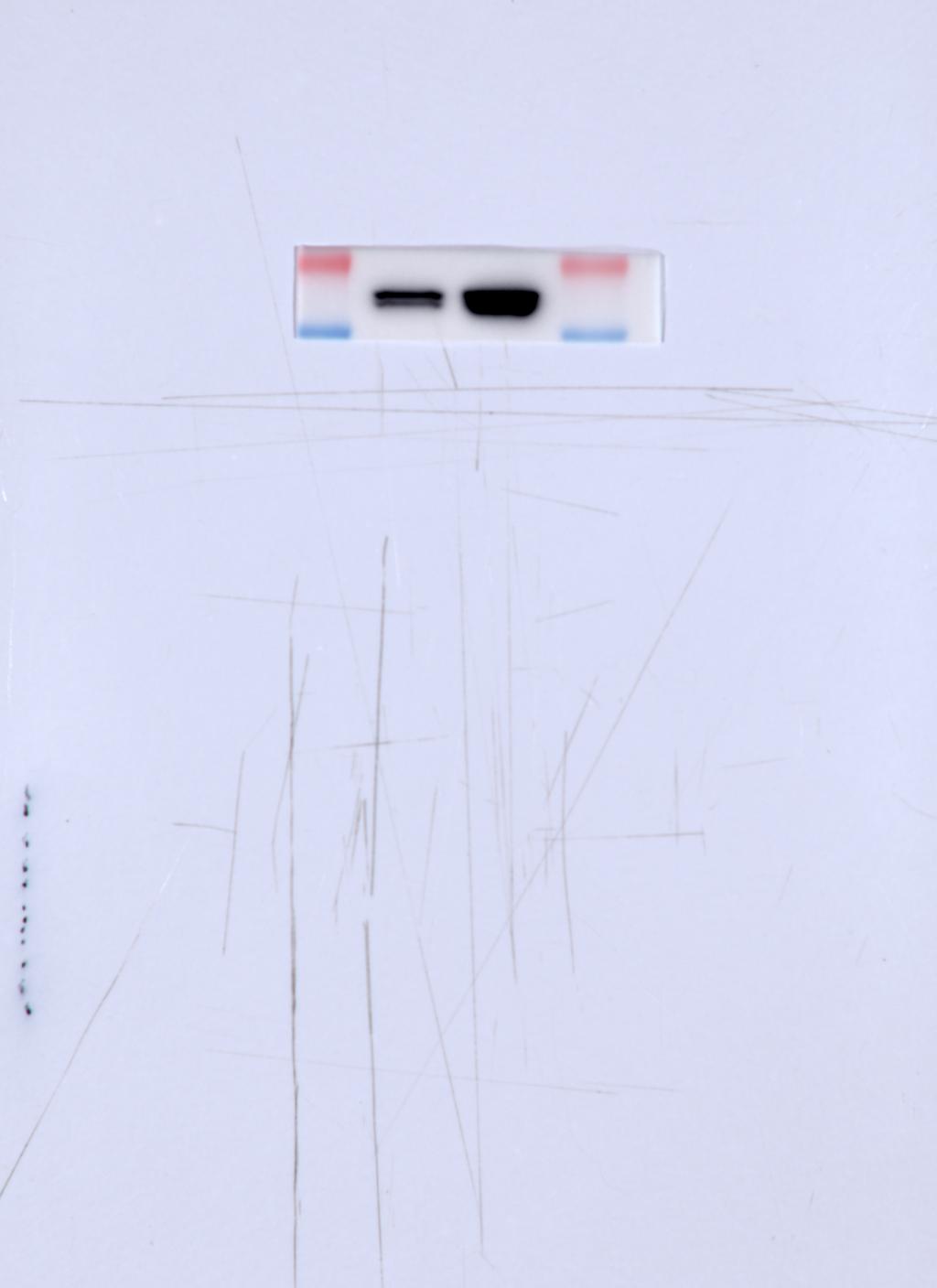


**Fig. 5E**


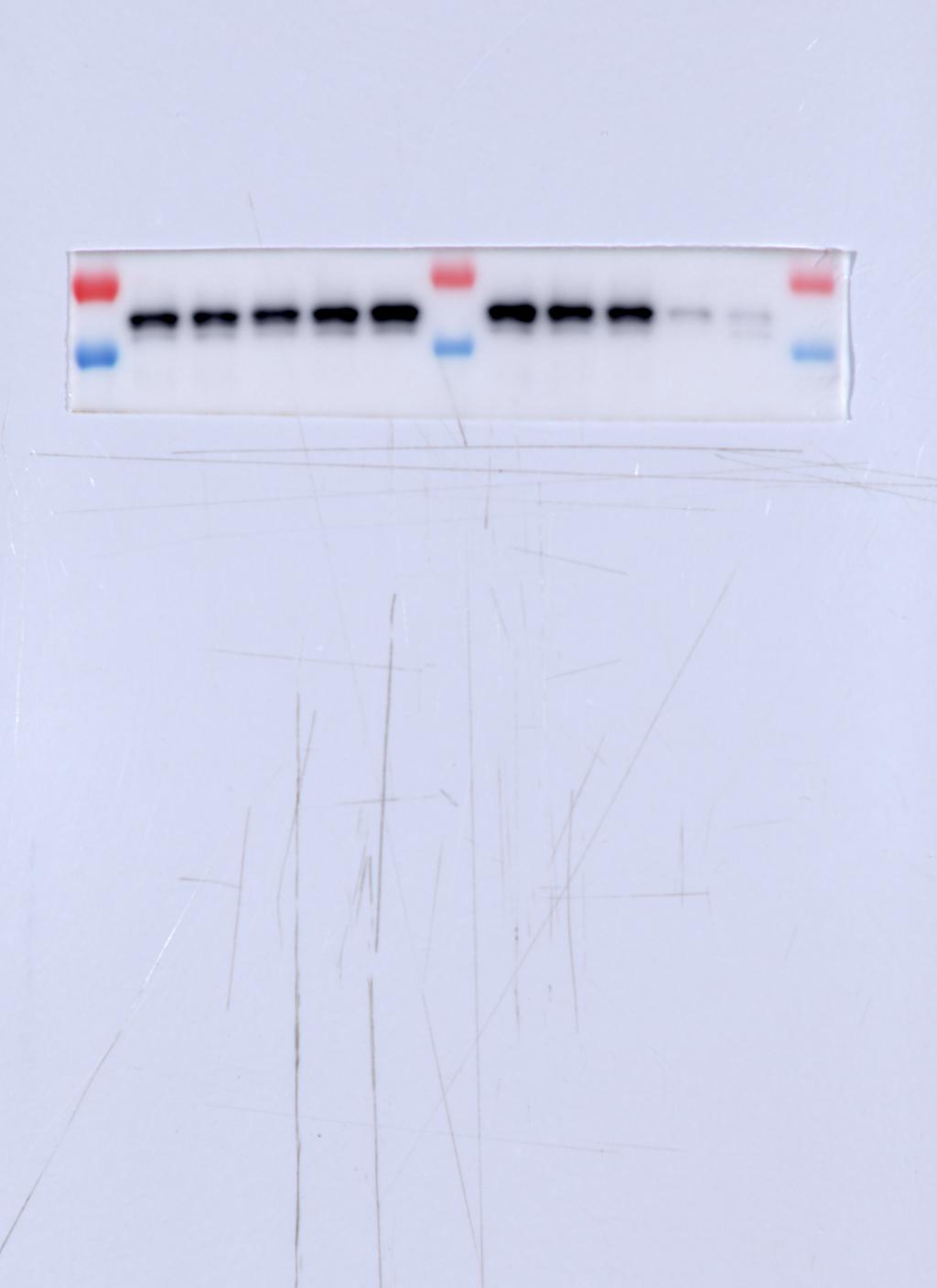


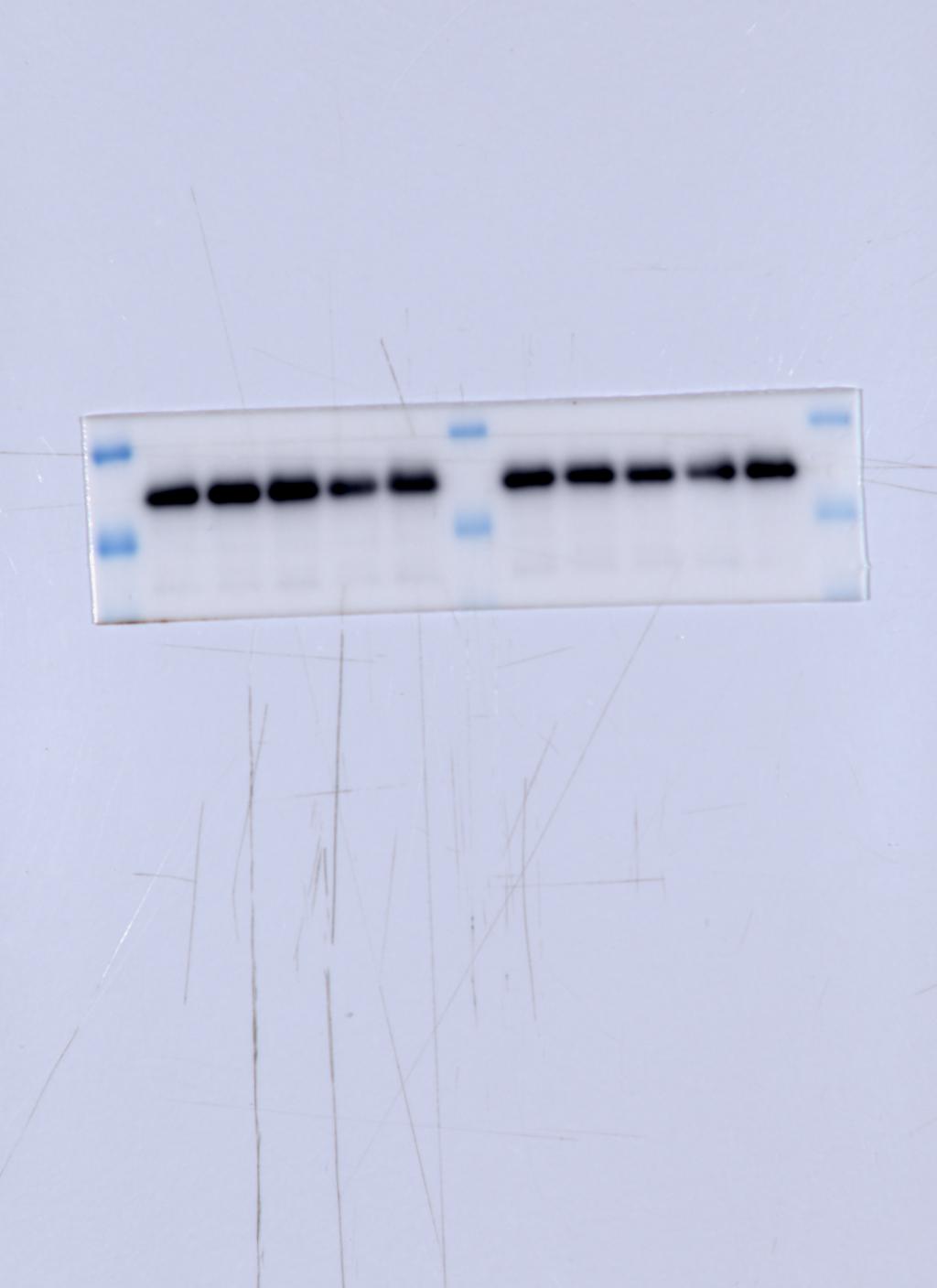


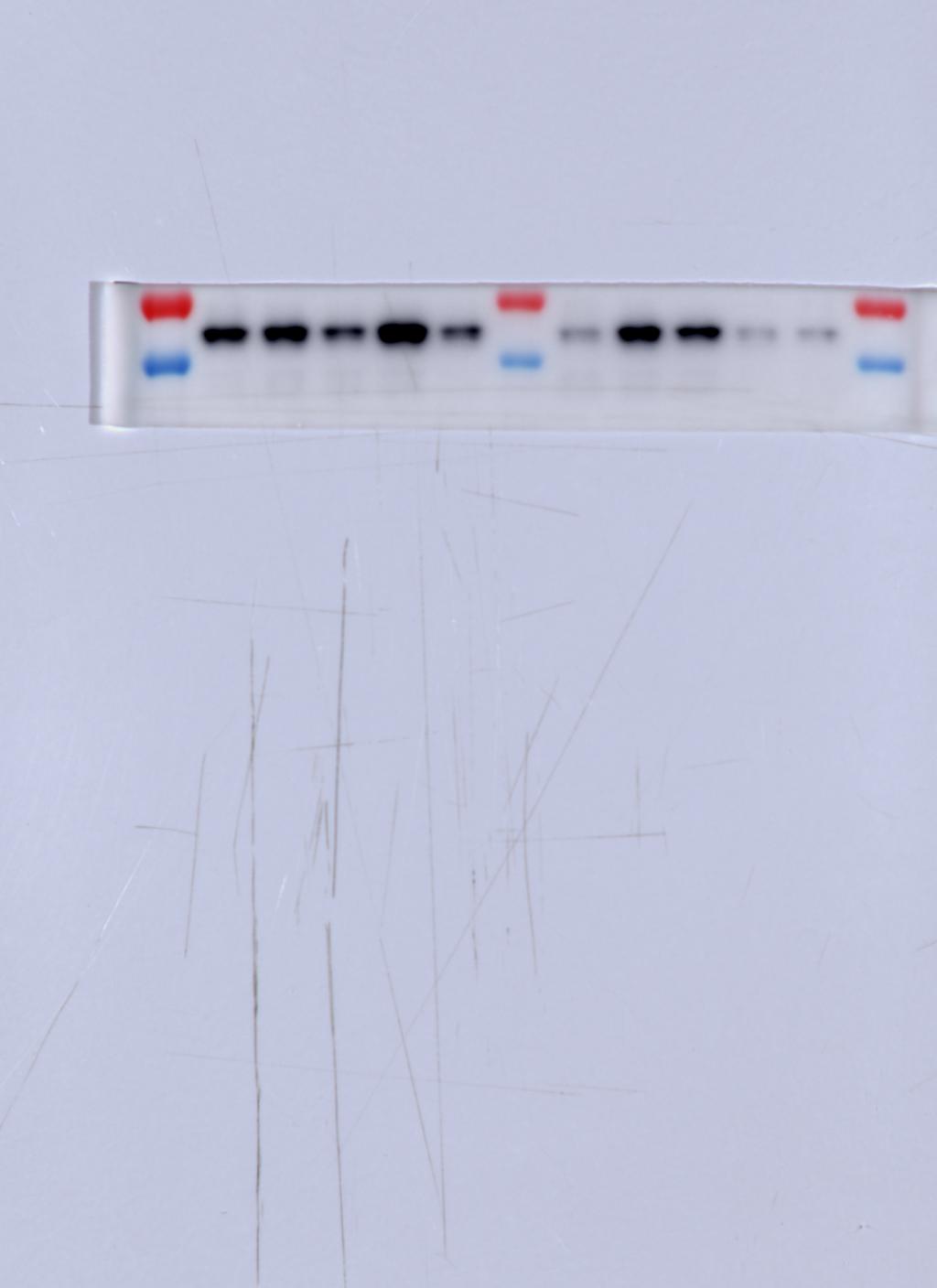


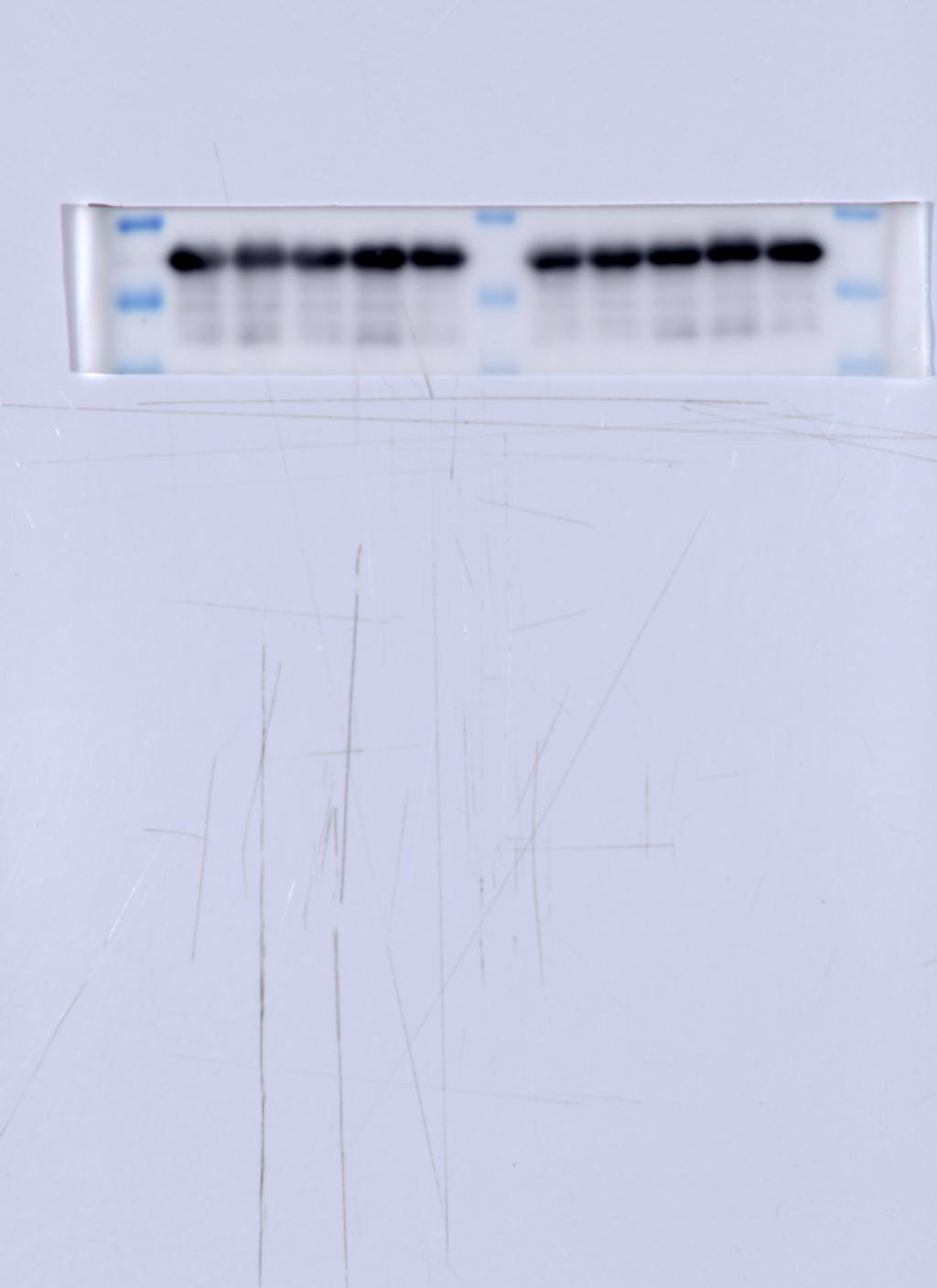


**Fig. 5F**


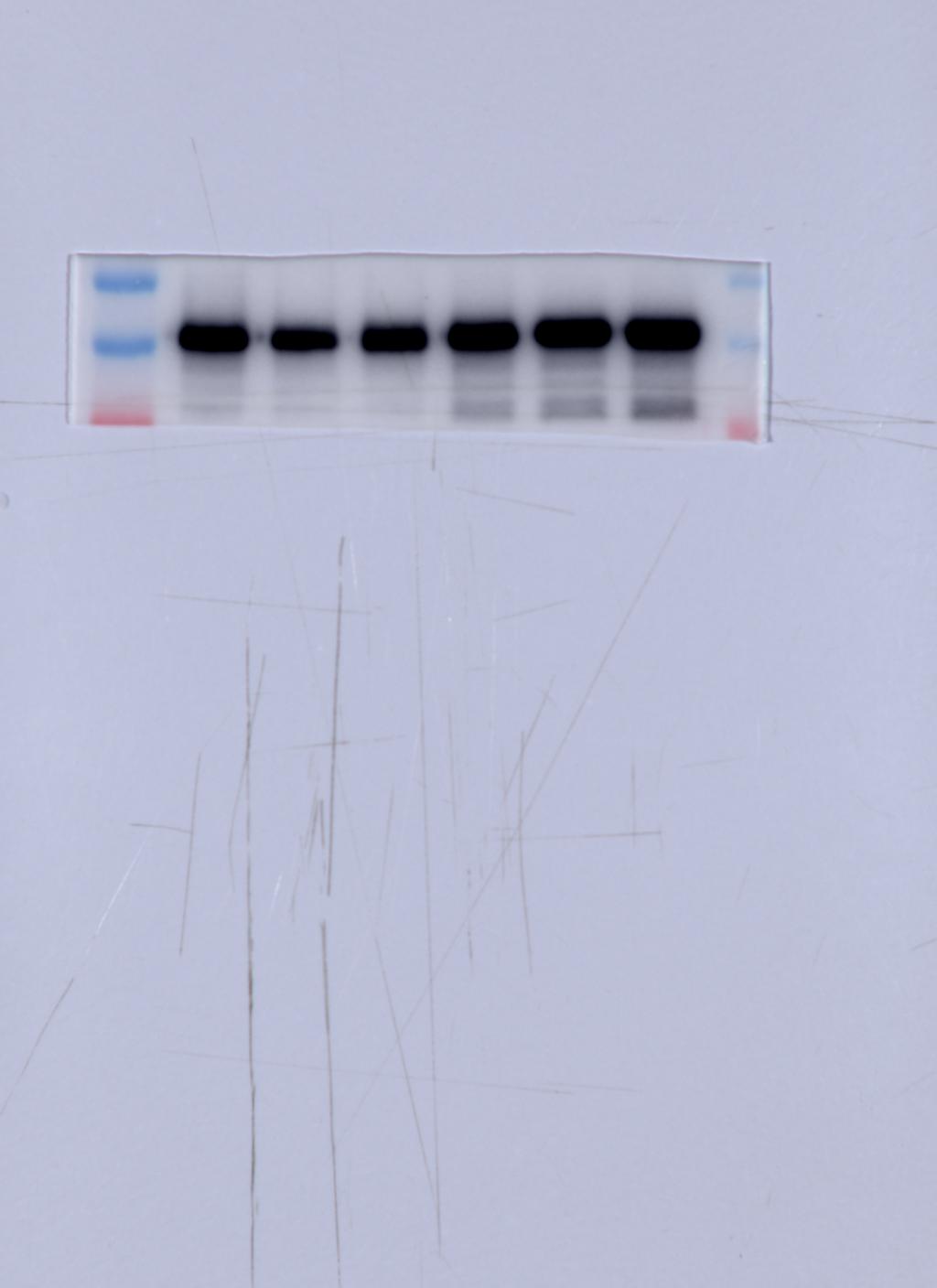

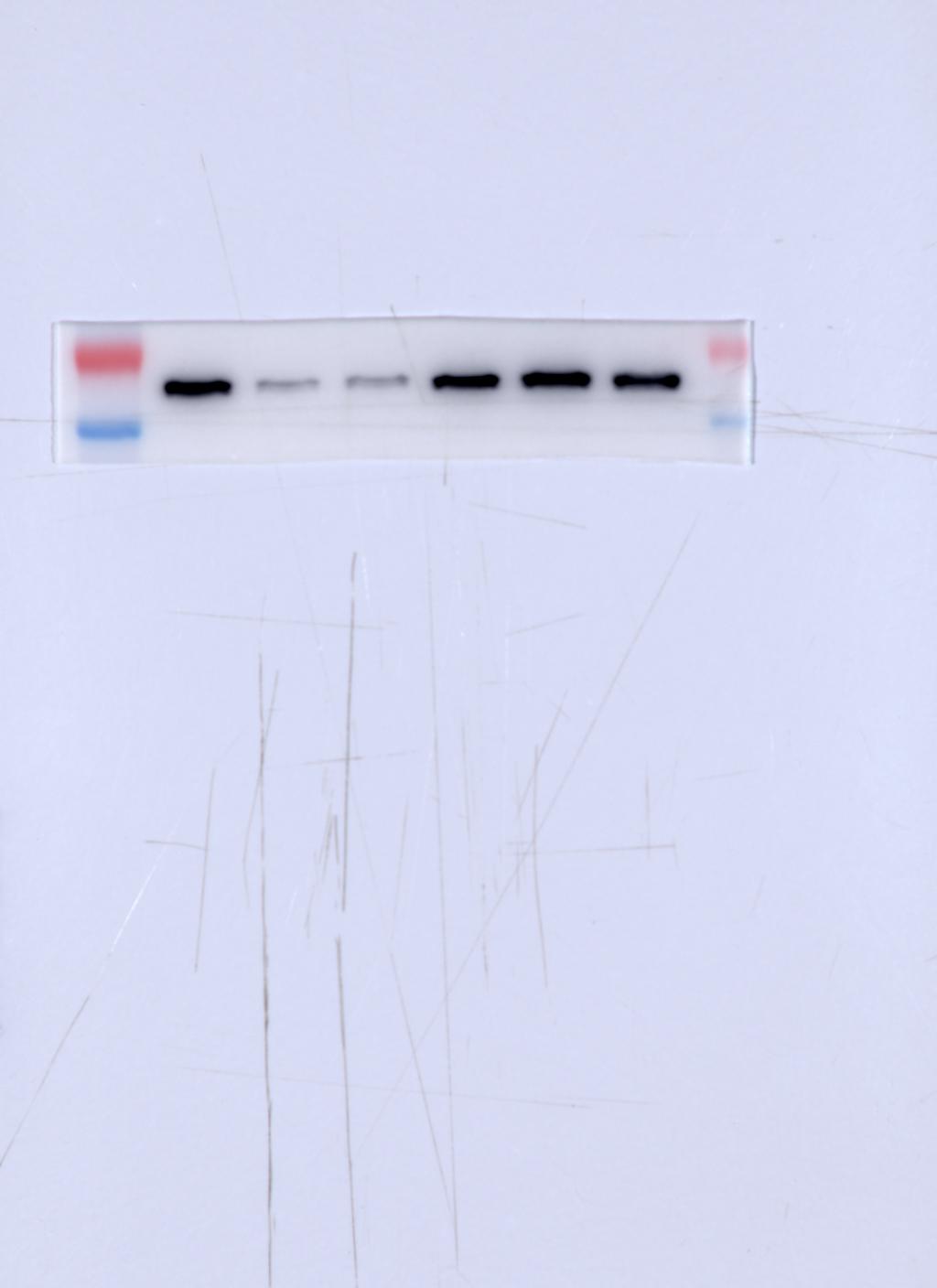

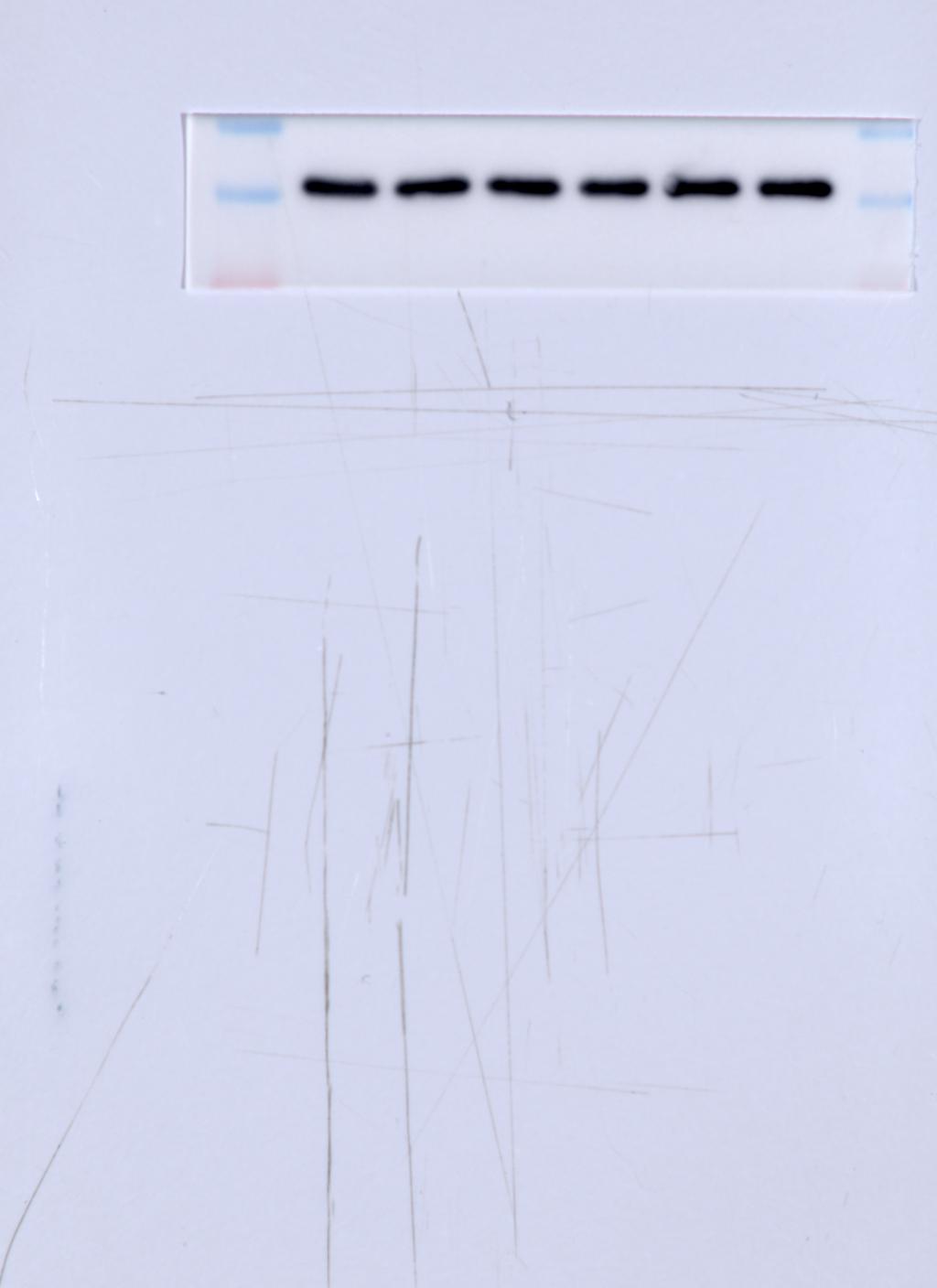

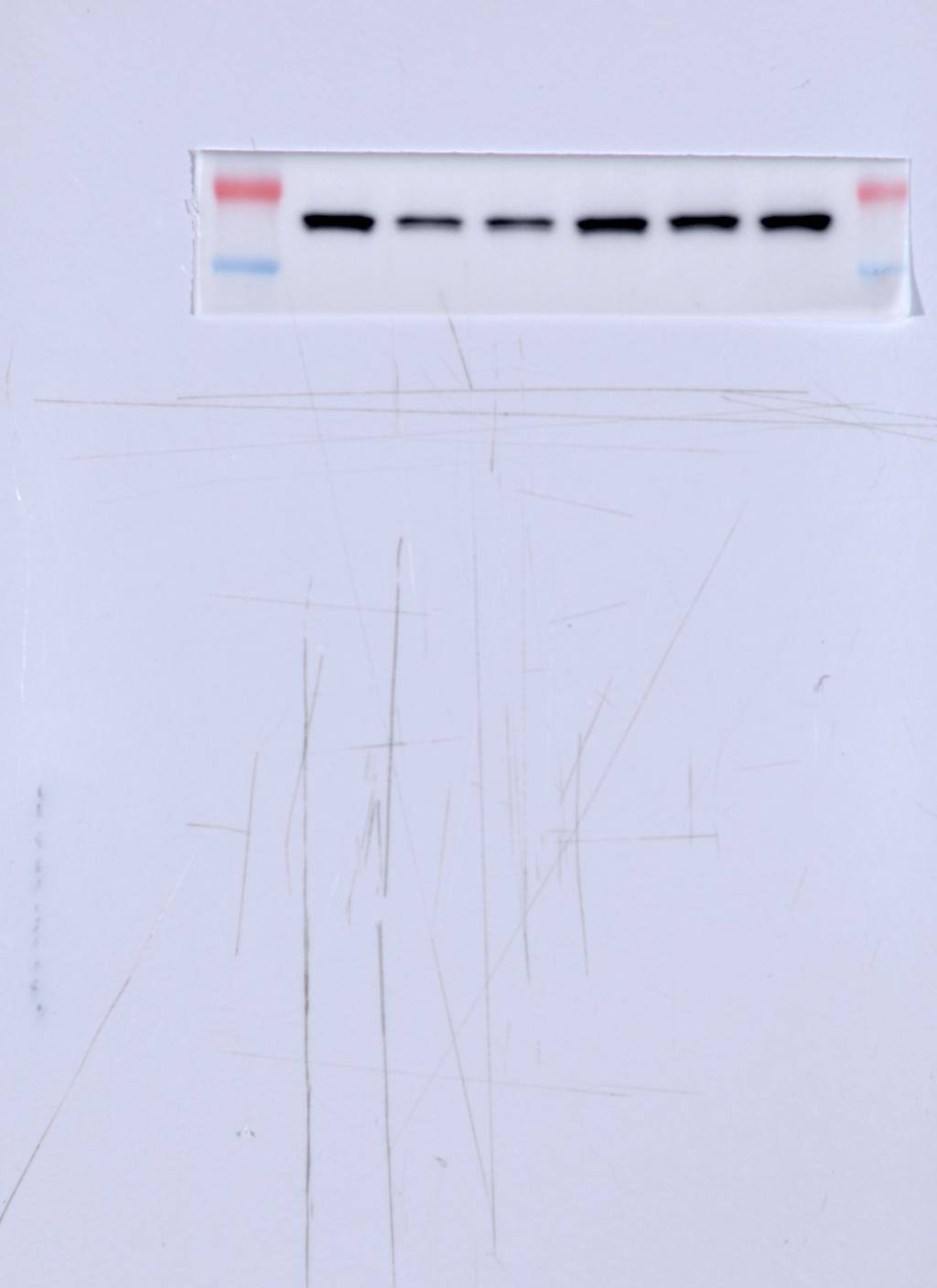


**Fig. 5G and H**


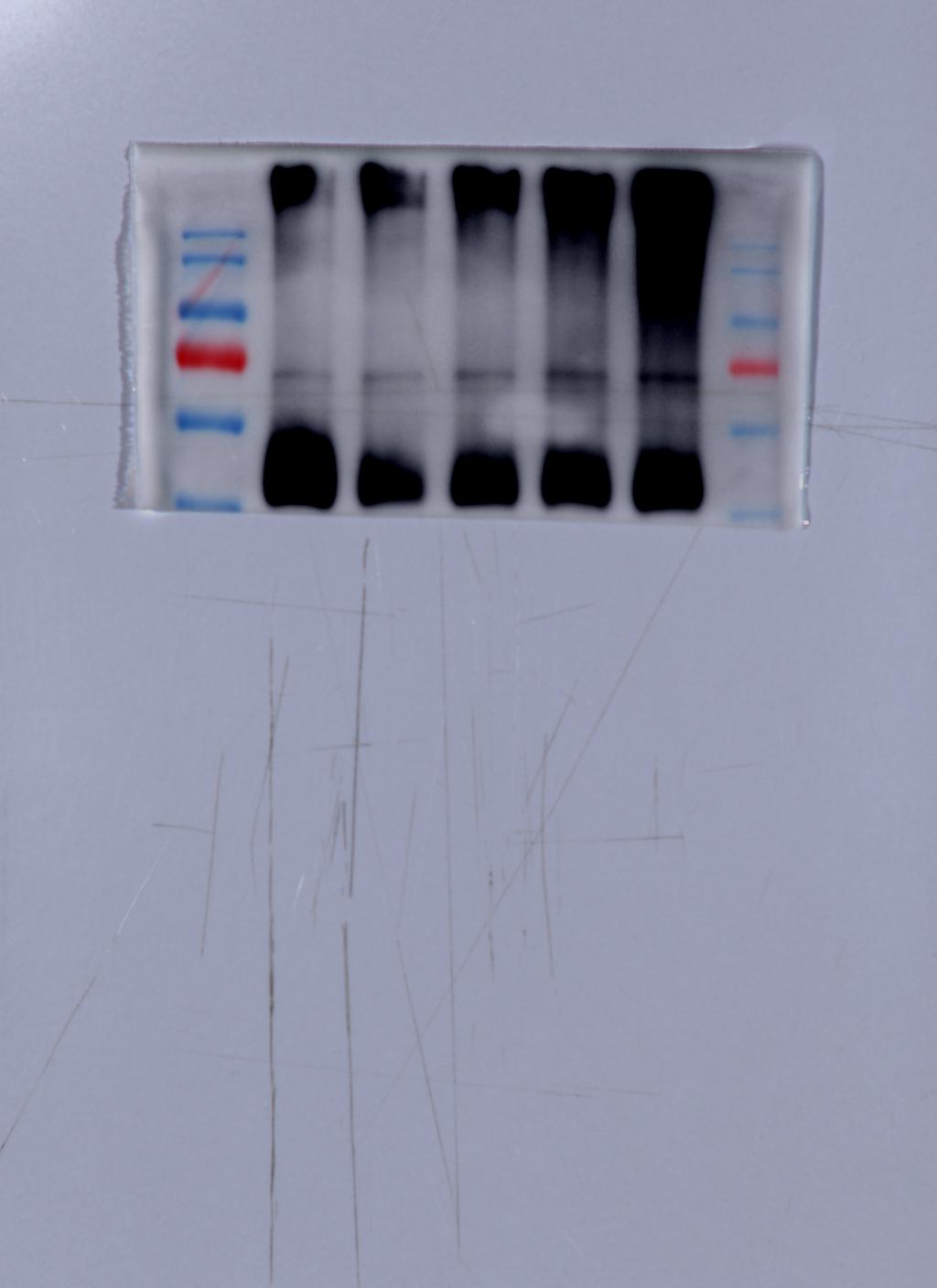


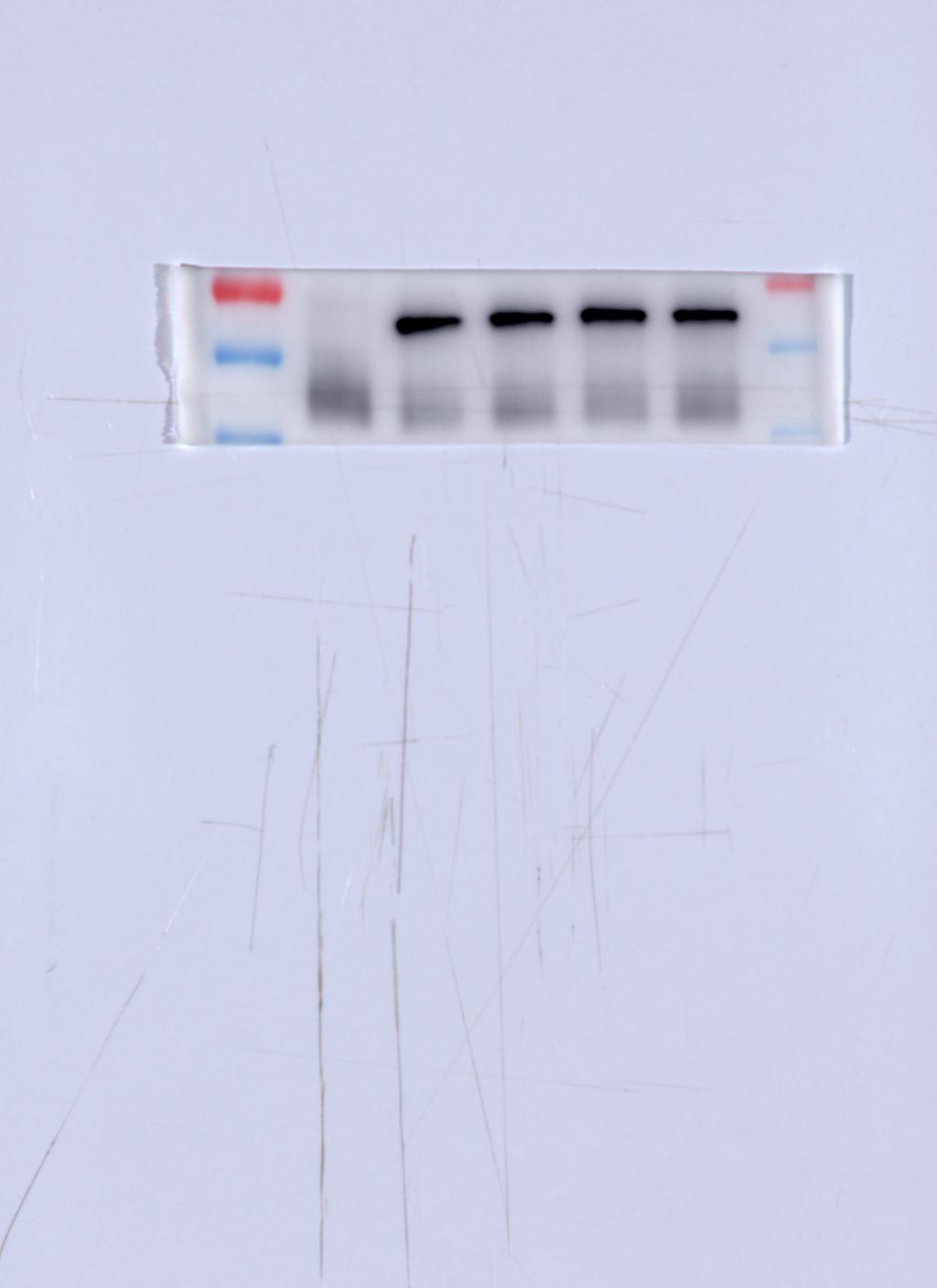


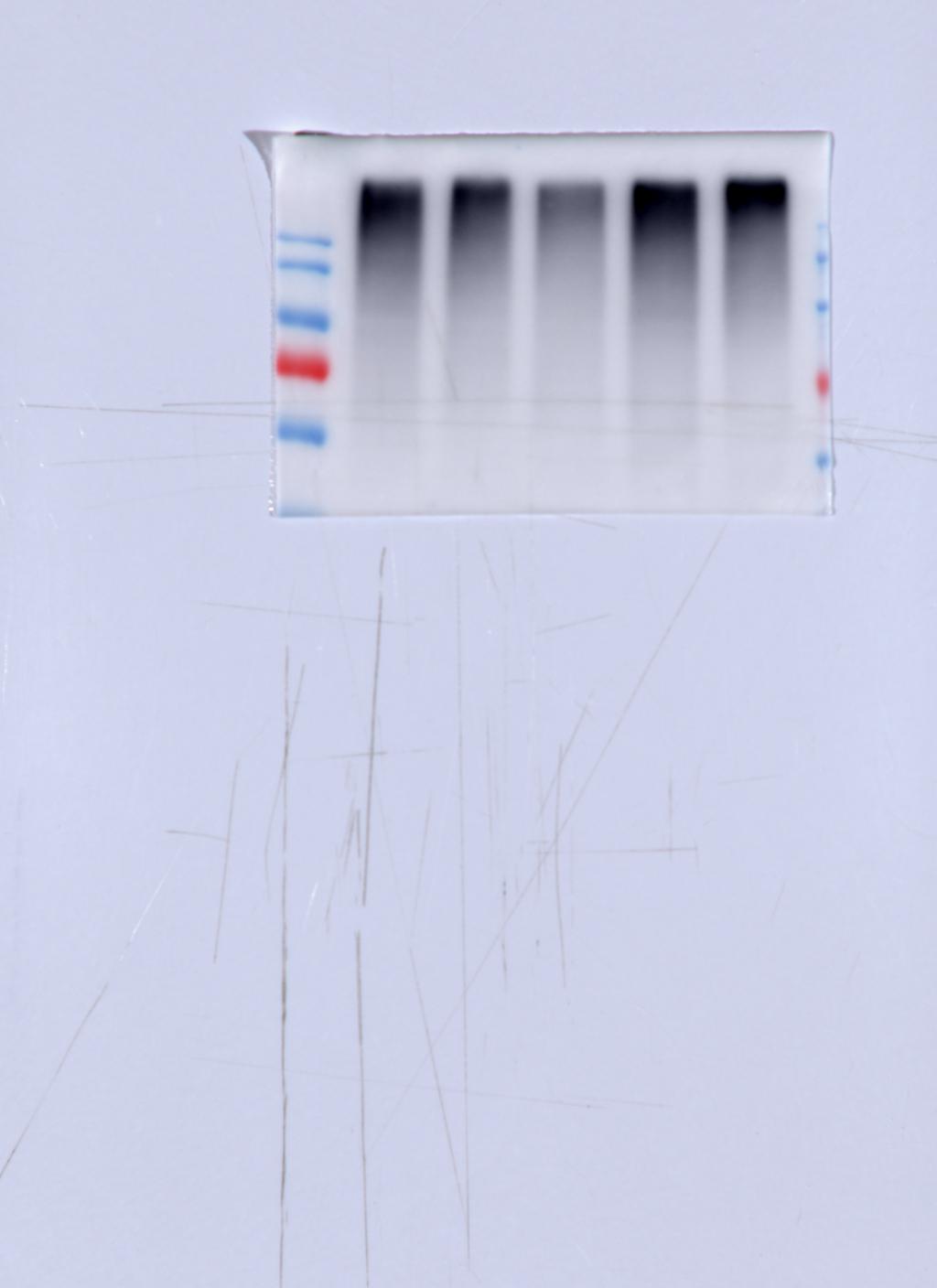


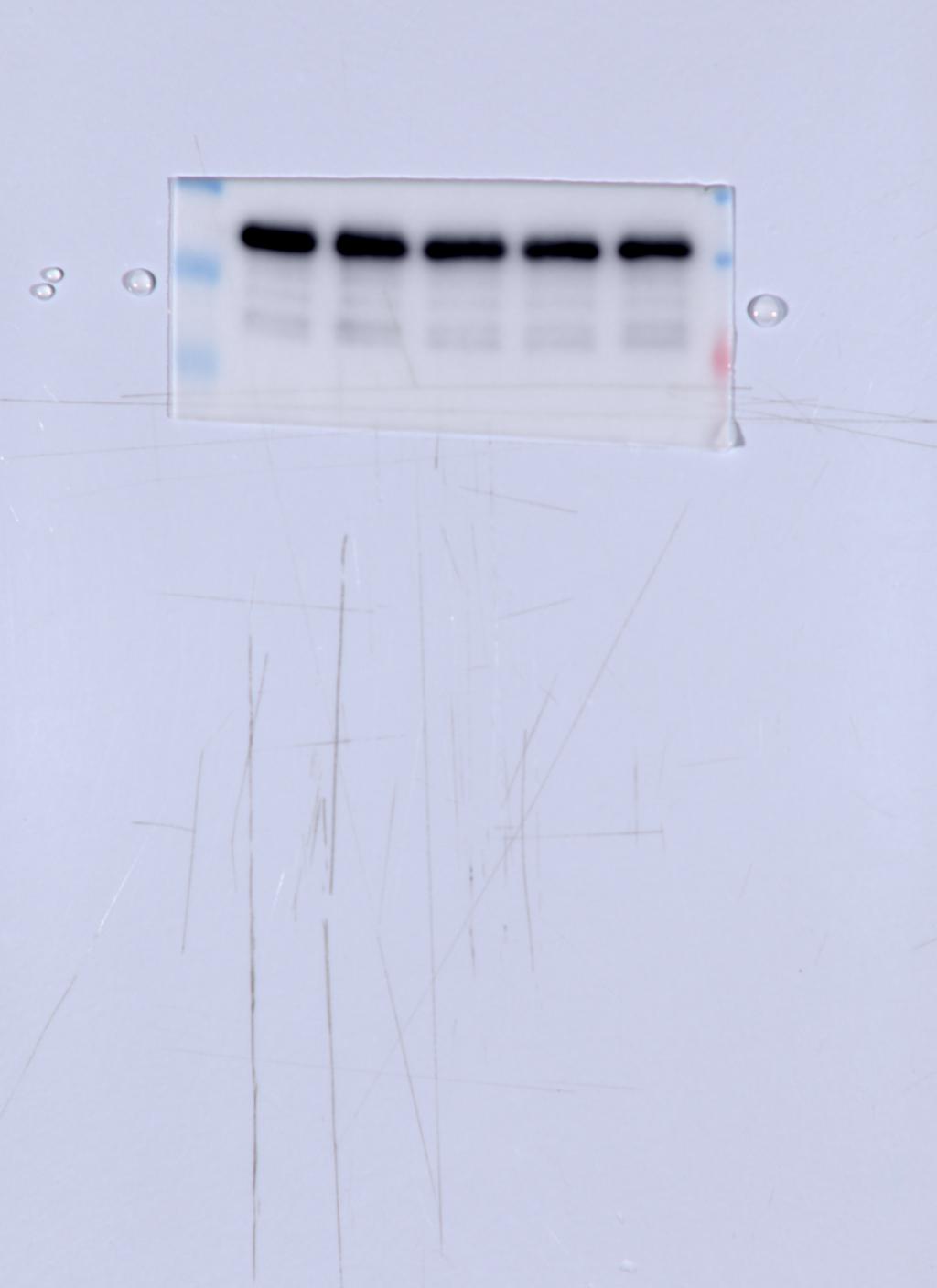

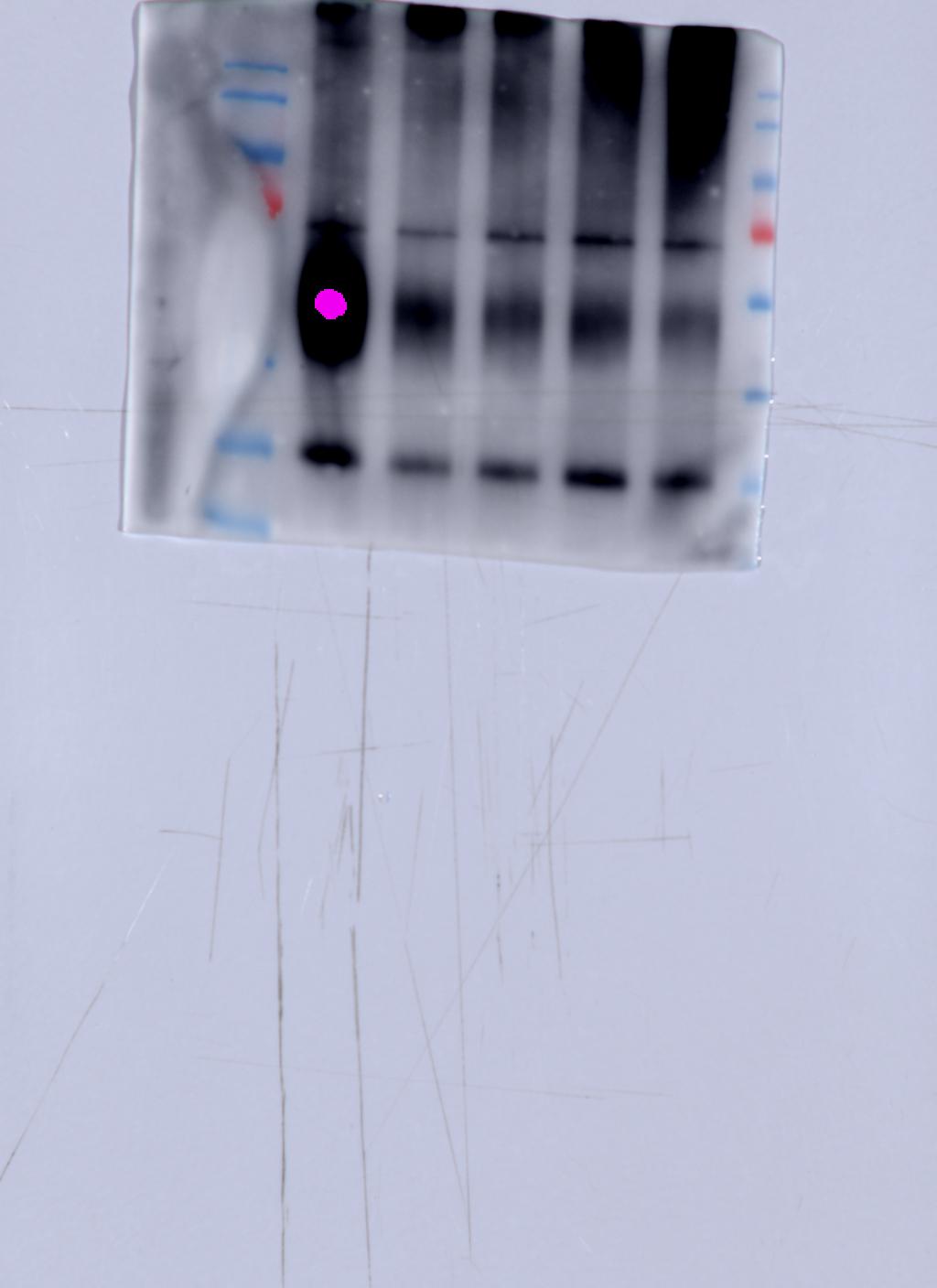


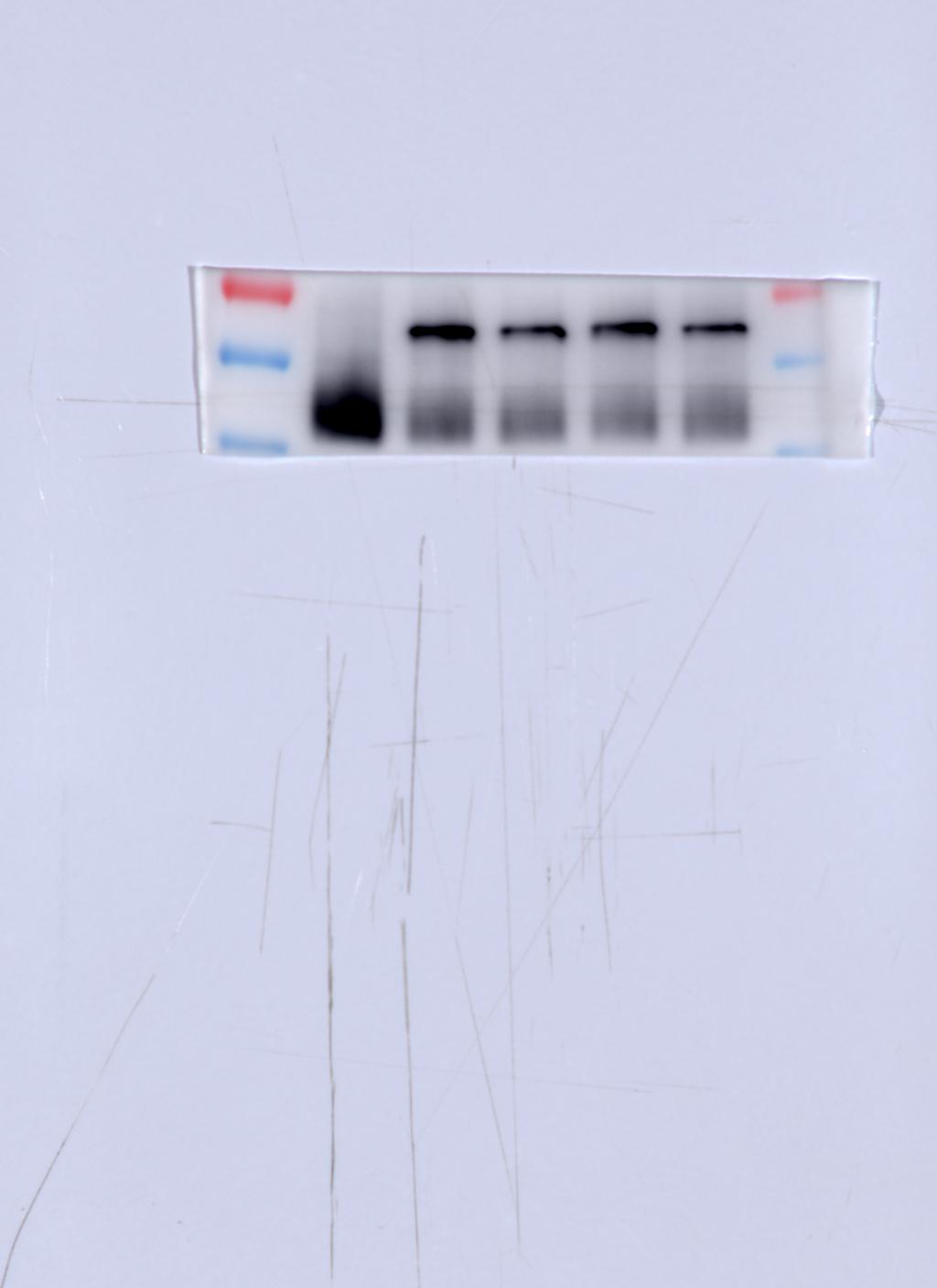


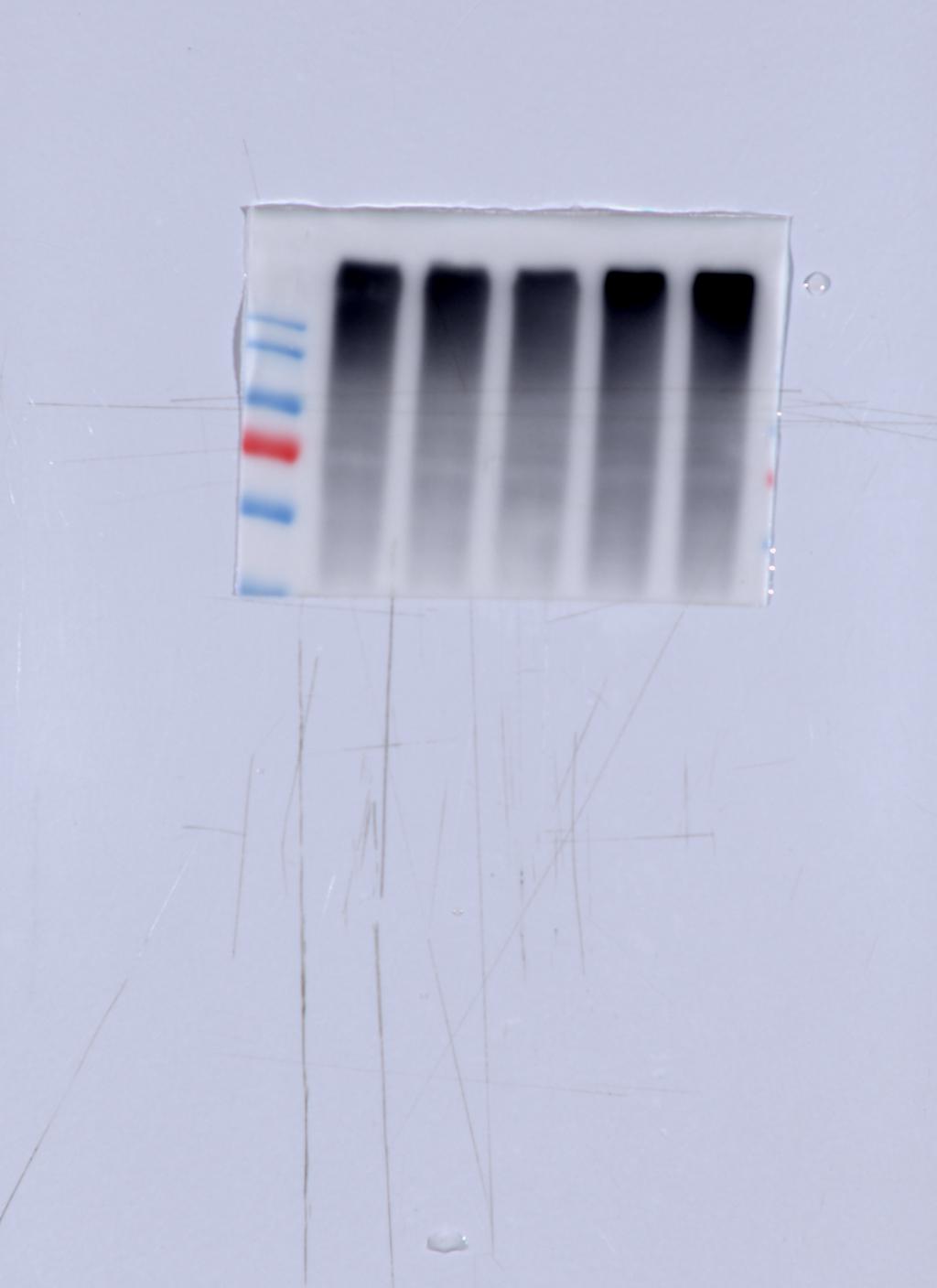


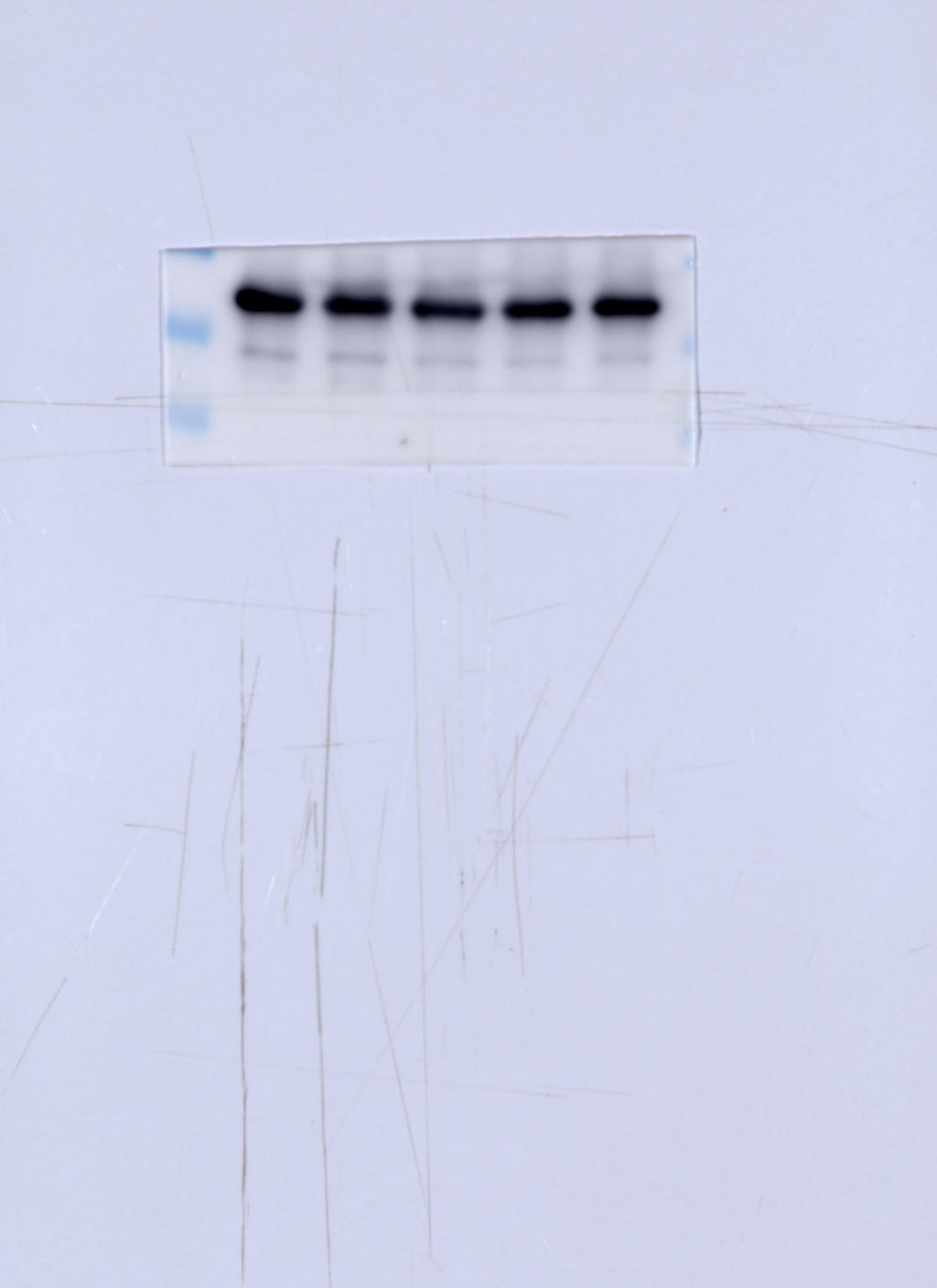


**Fig. 6E**


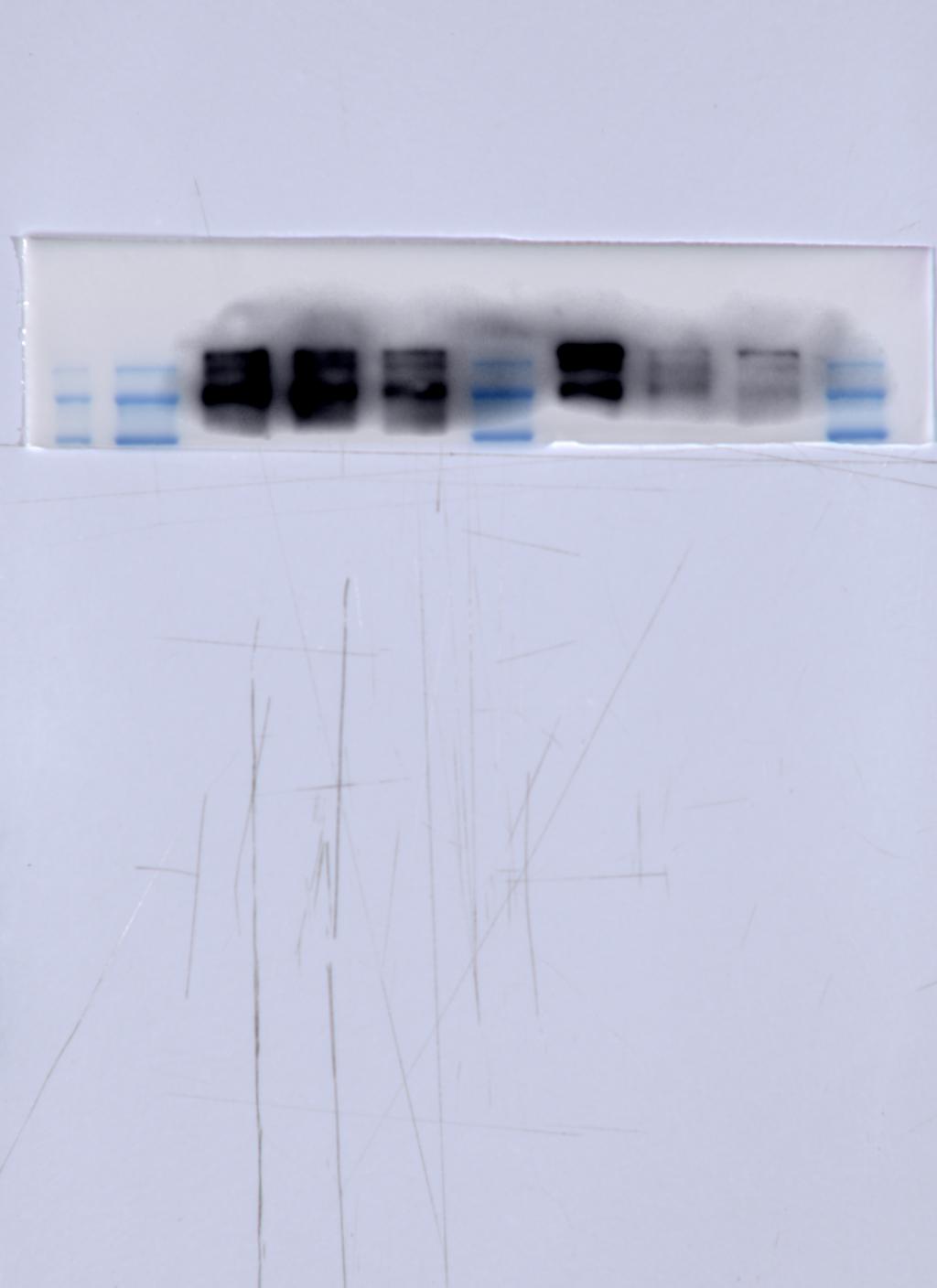

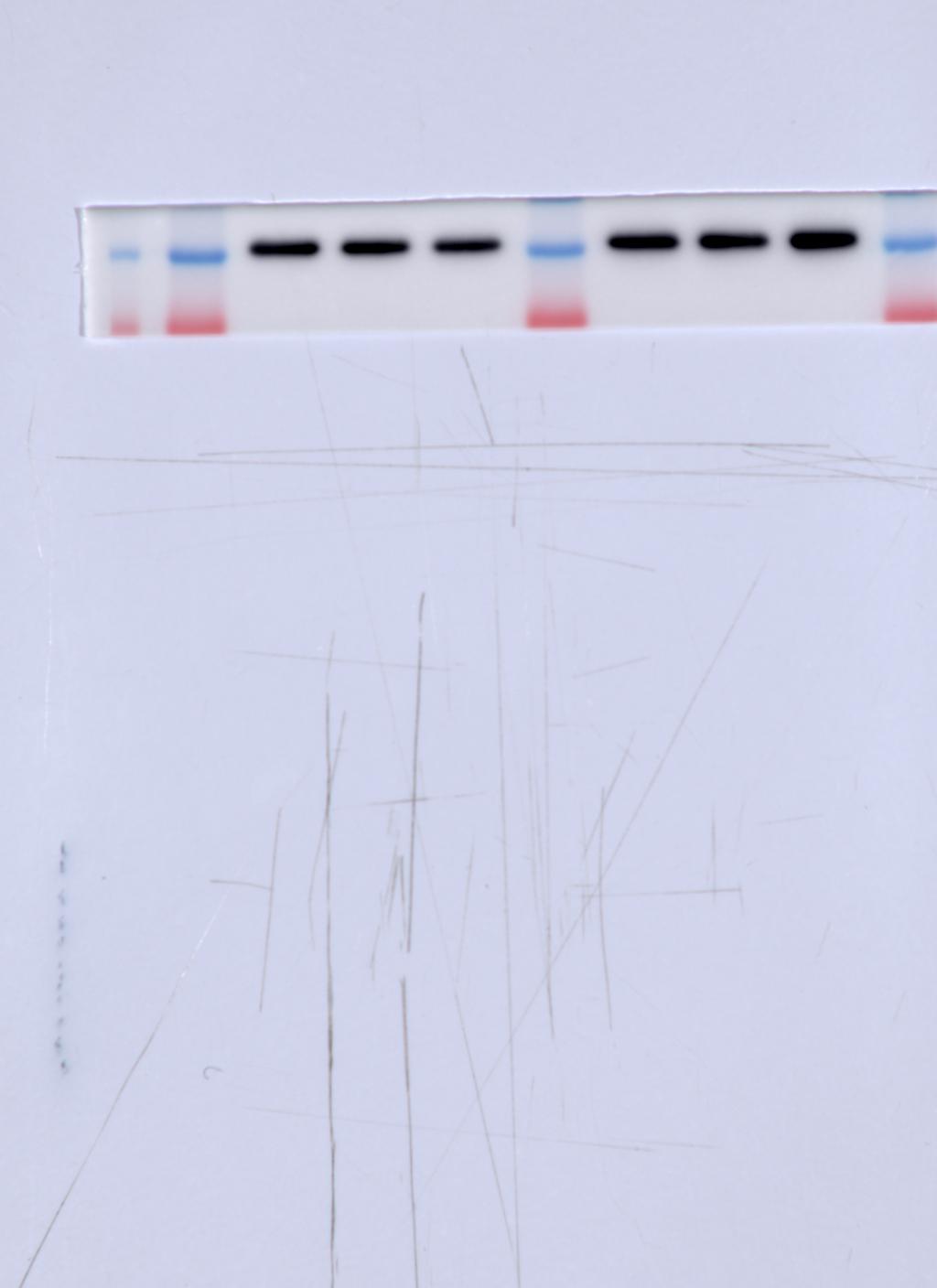

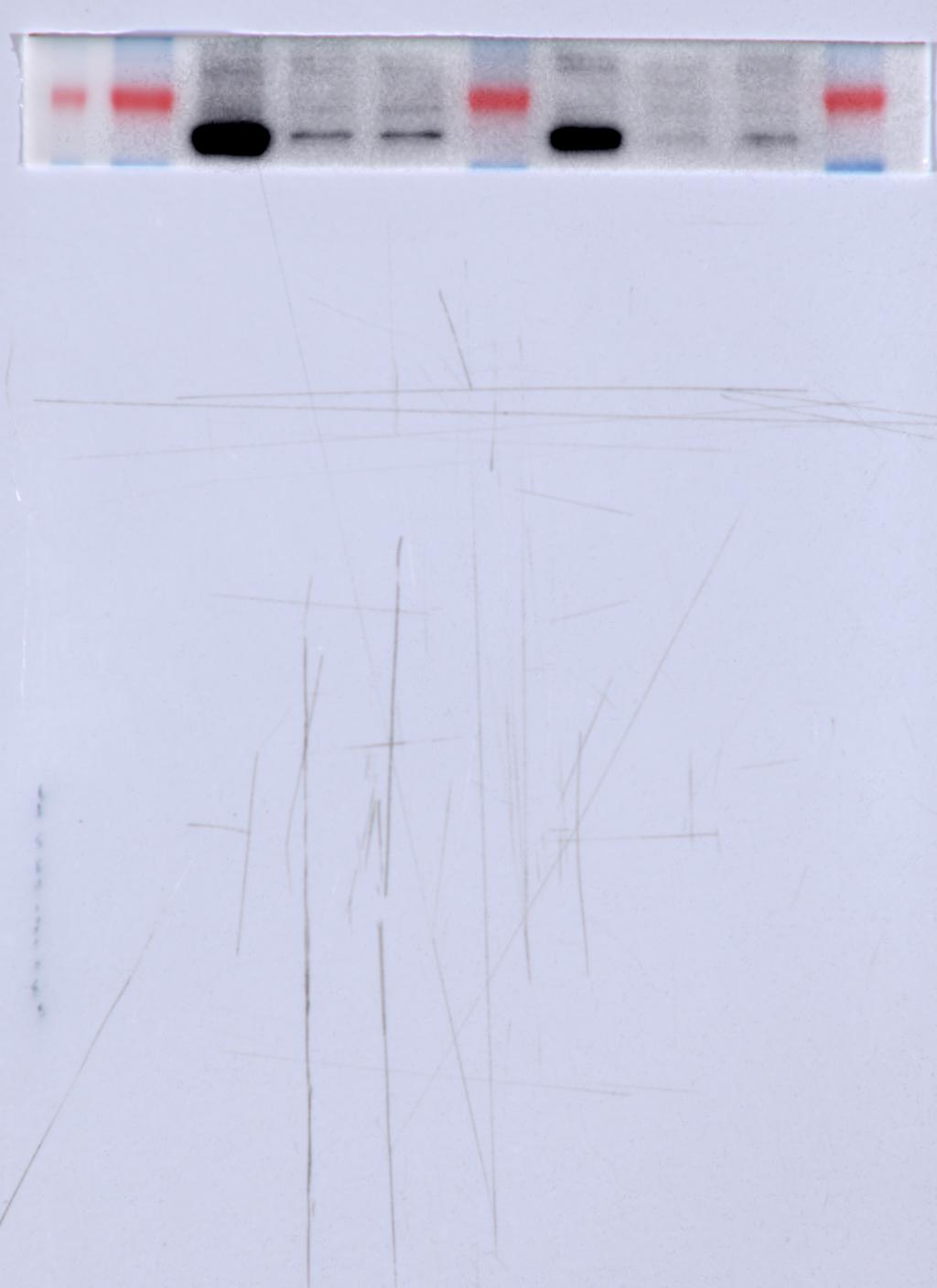


**Fig. 6J**


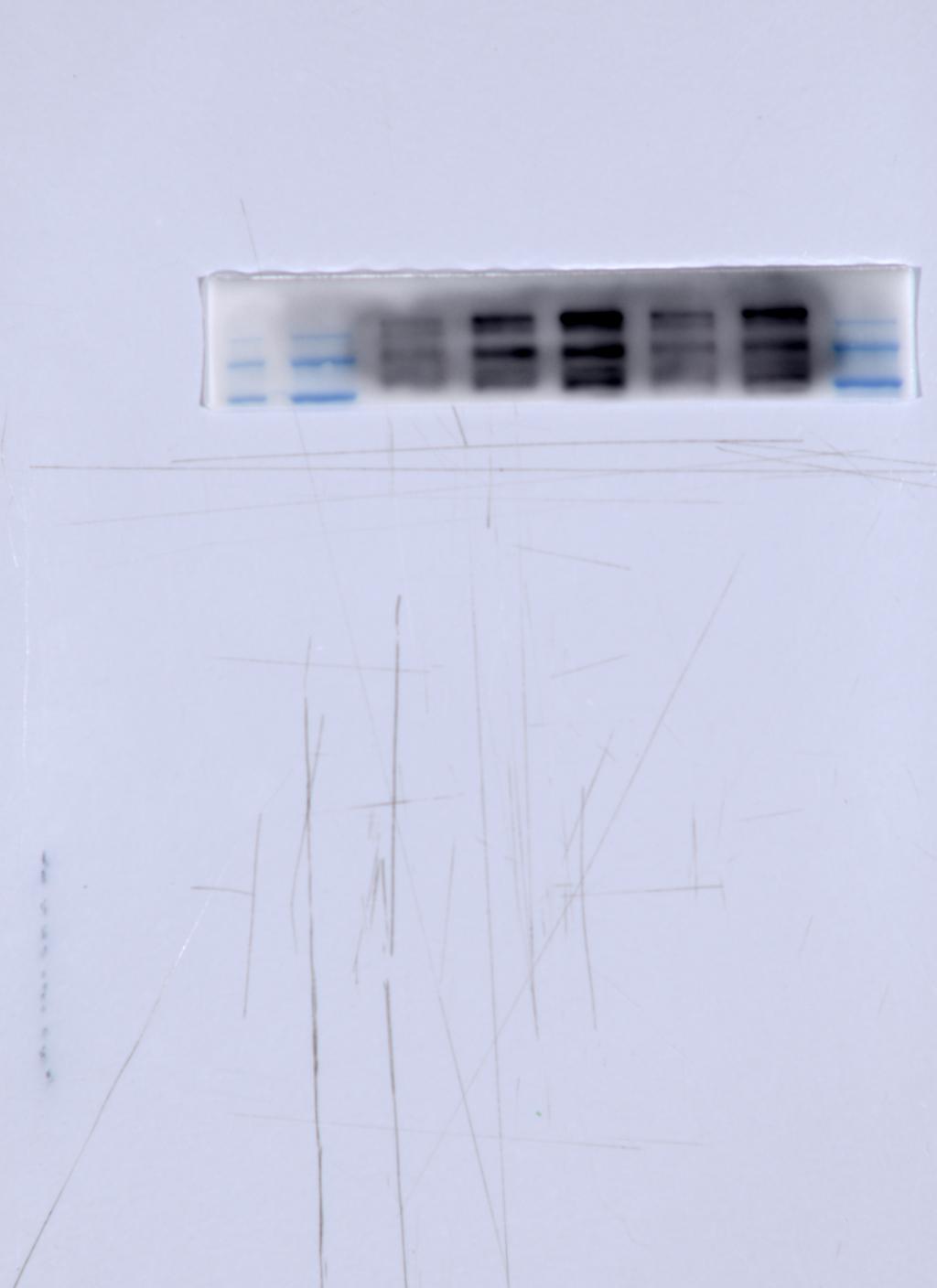

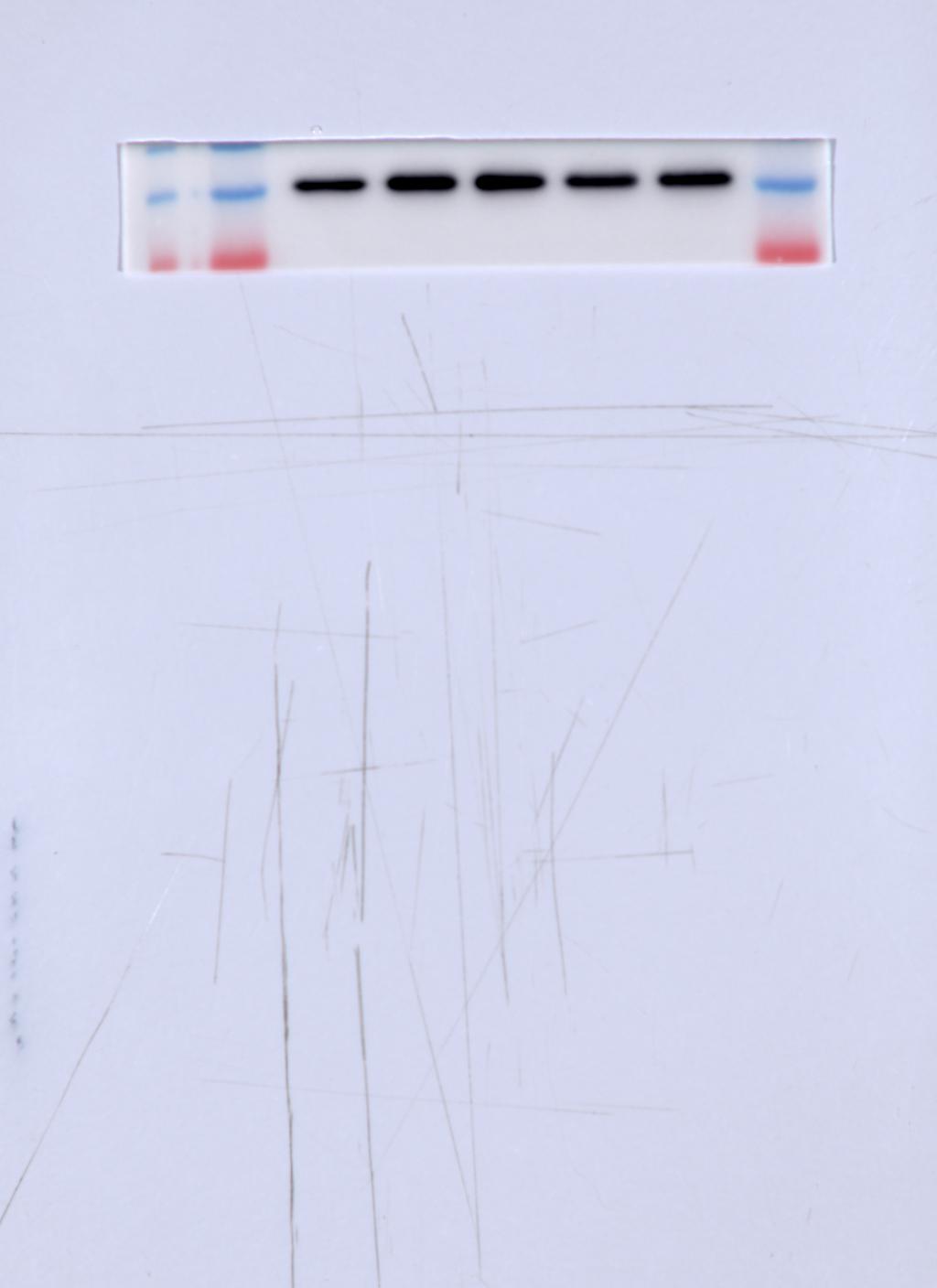

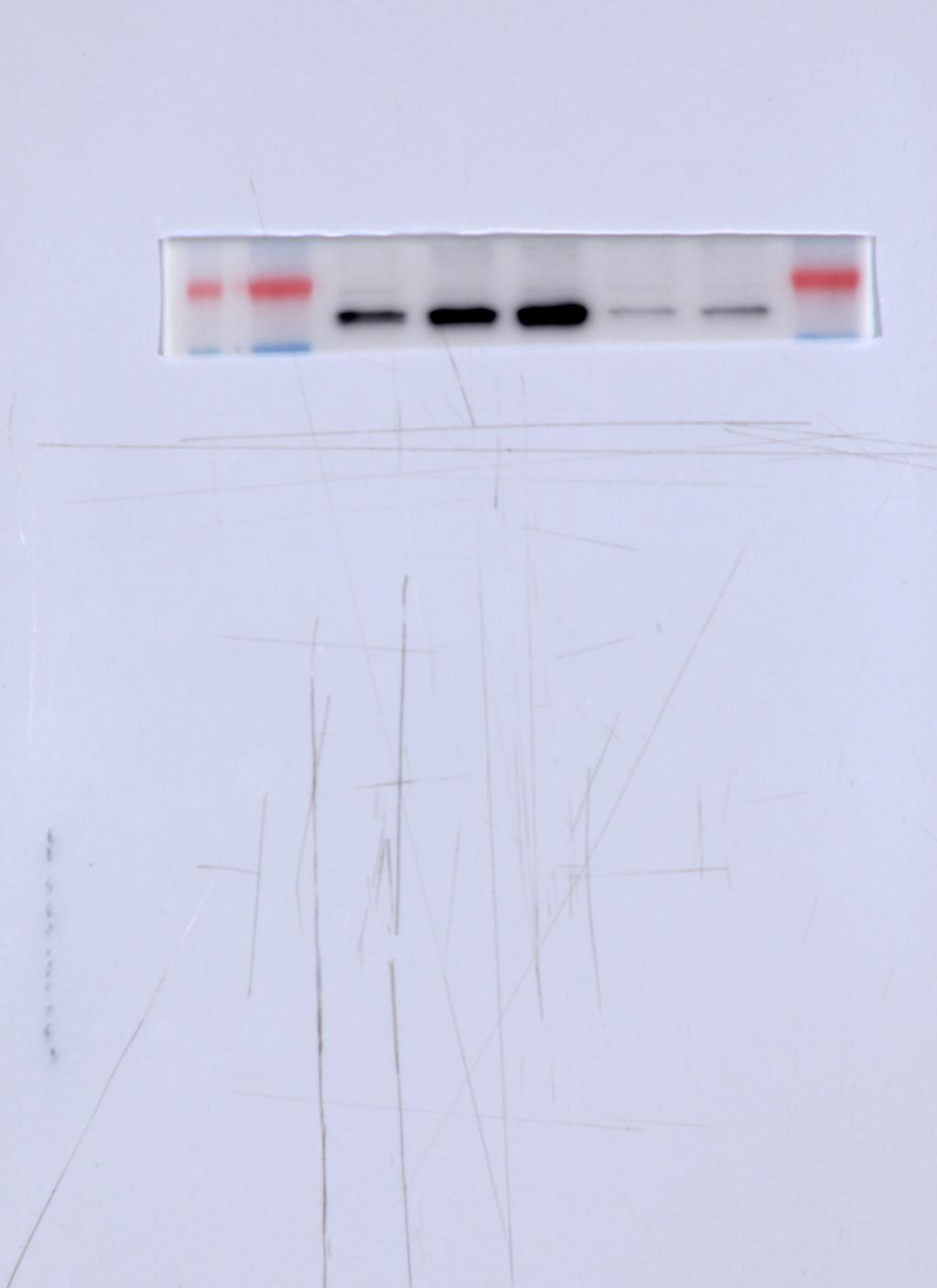


**Fig. 8H**


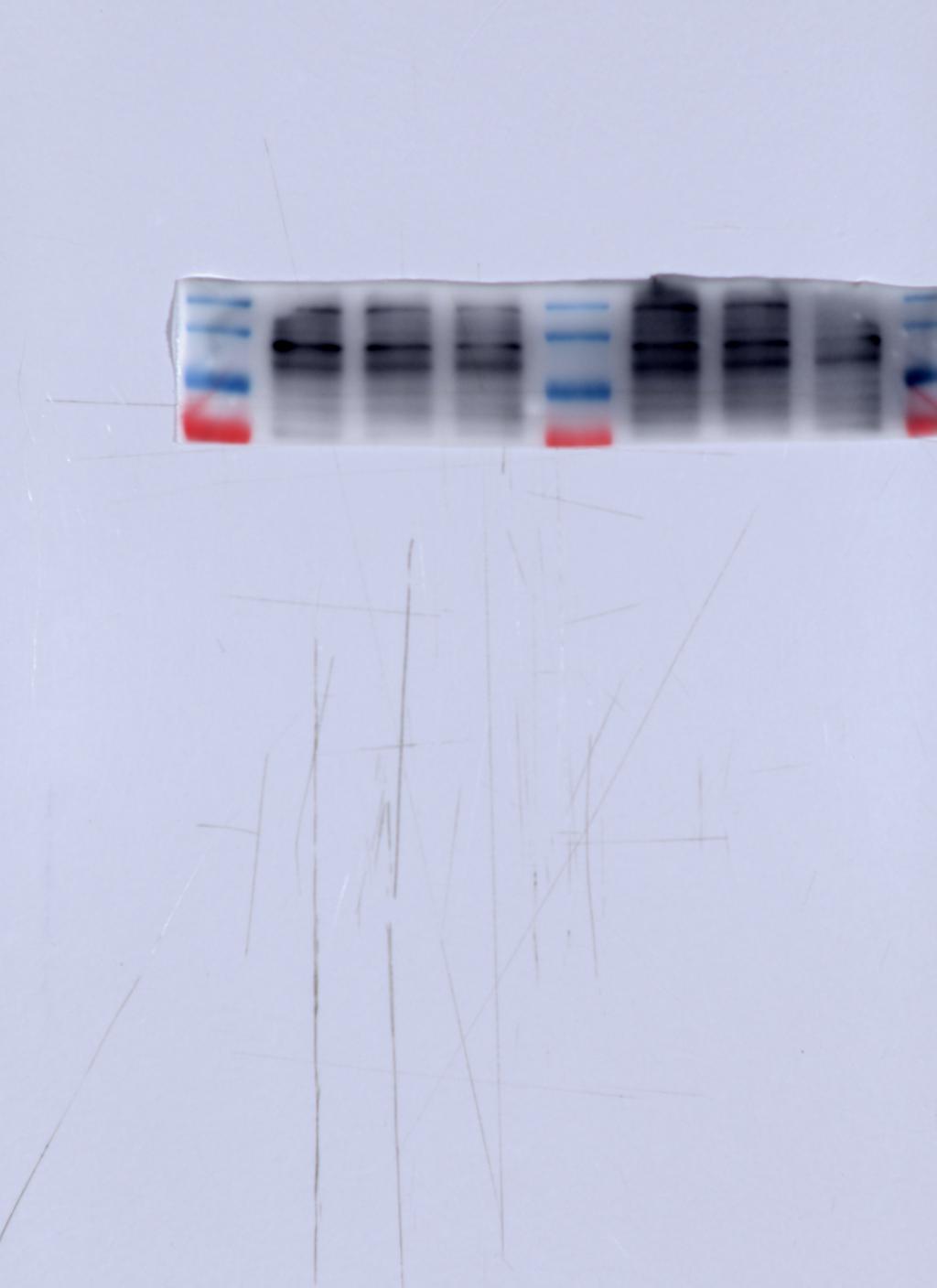


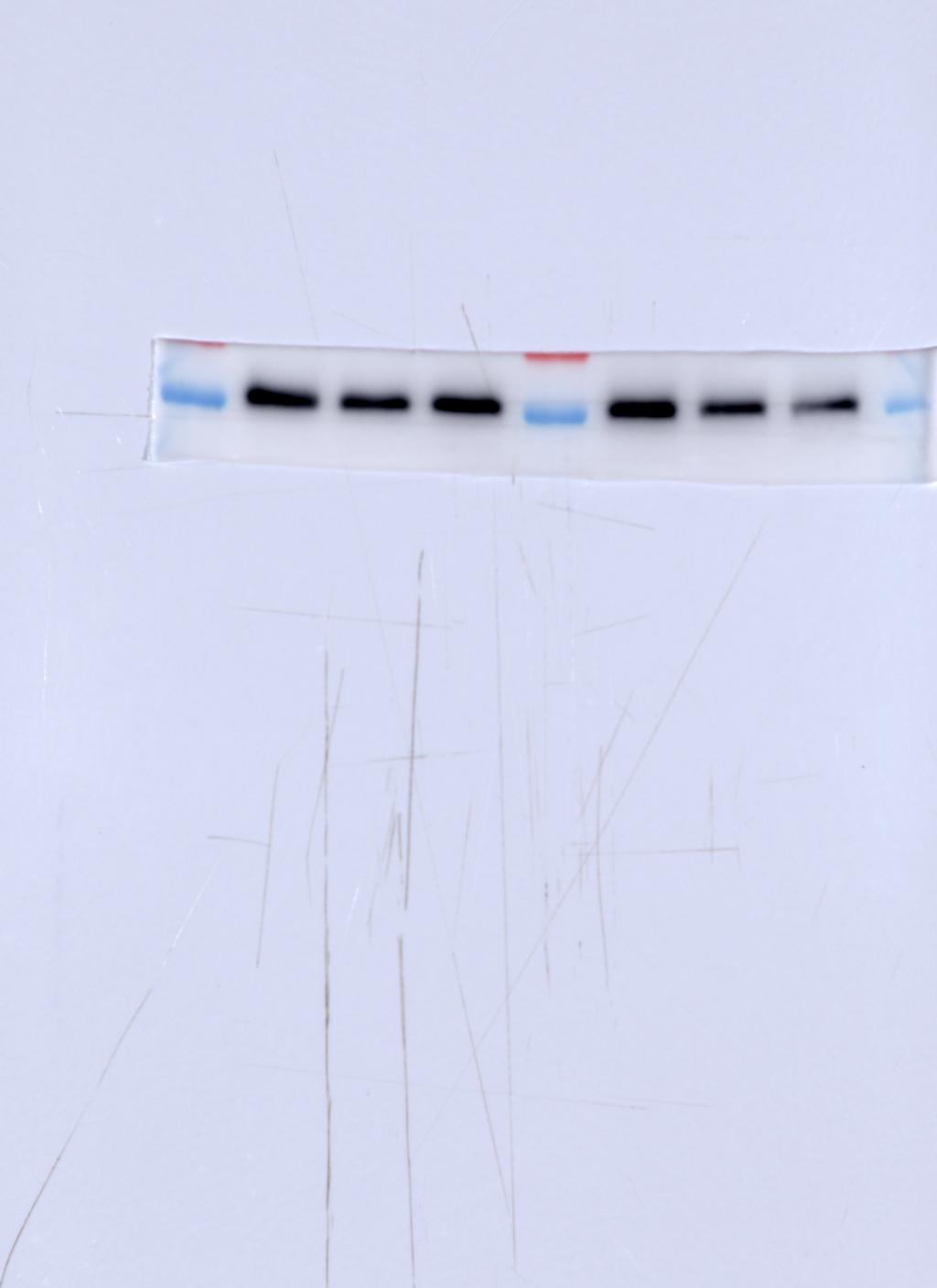

Supplement: Supplementary file 6 — raw data [file 41419_2022_5540_MOESM6_ESM.docx]
